# Supplementary figures and images for: Enhanced human pose estimation using YOLOv8 with Integrated SimDLKA attention mechanism and DCIOU loss function: Analysis of human body behavior and posture (part 2 of 2)
Source: PLoS One. 2025 May 7;20(5):e0318578. doi: 10.1371/journal.pone.0318578 (PMC12057905; doi:10.1371/journal.pone.0318578)

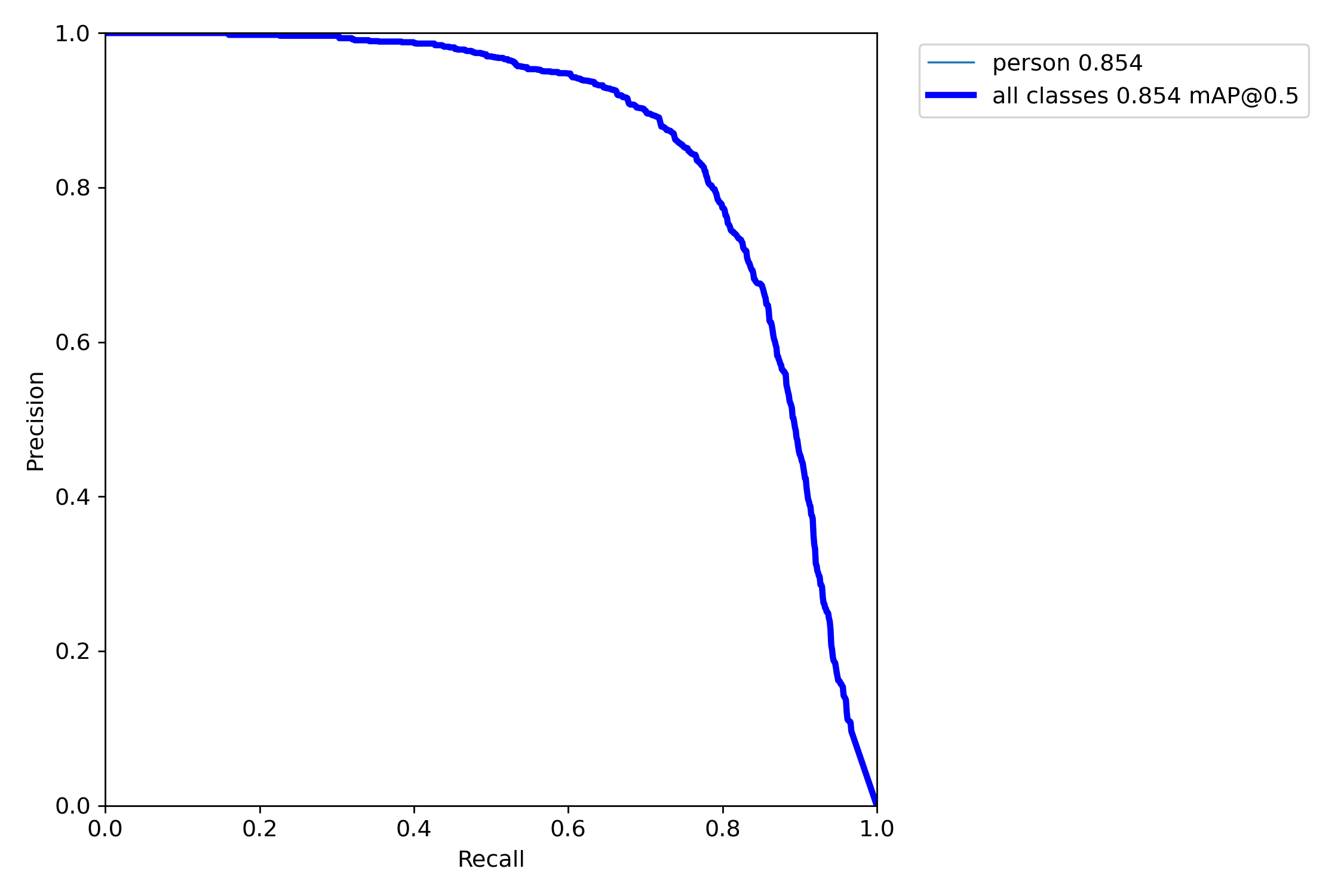

Supplement: S1 File — (ZIP) [file pone.0318578.s002.zip › suooprt information/pose/train34/BoxPR_curve.png]

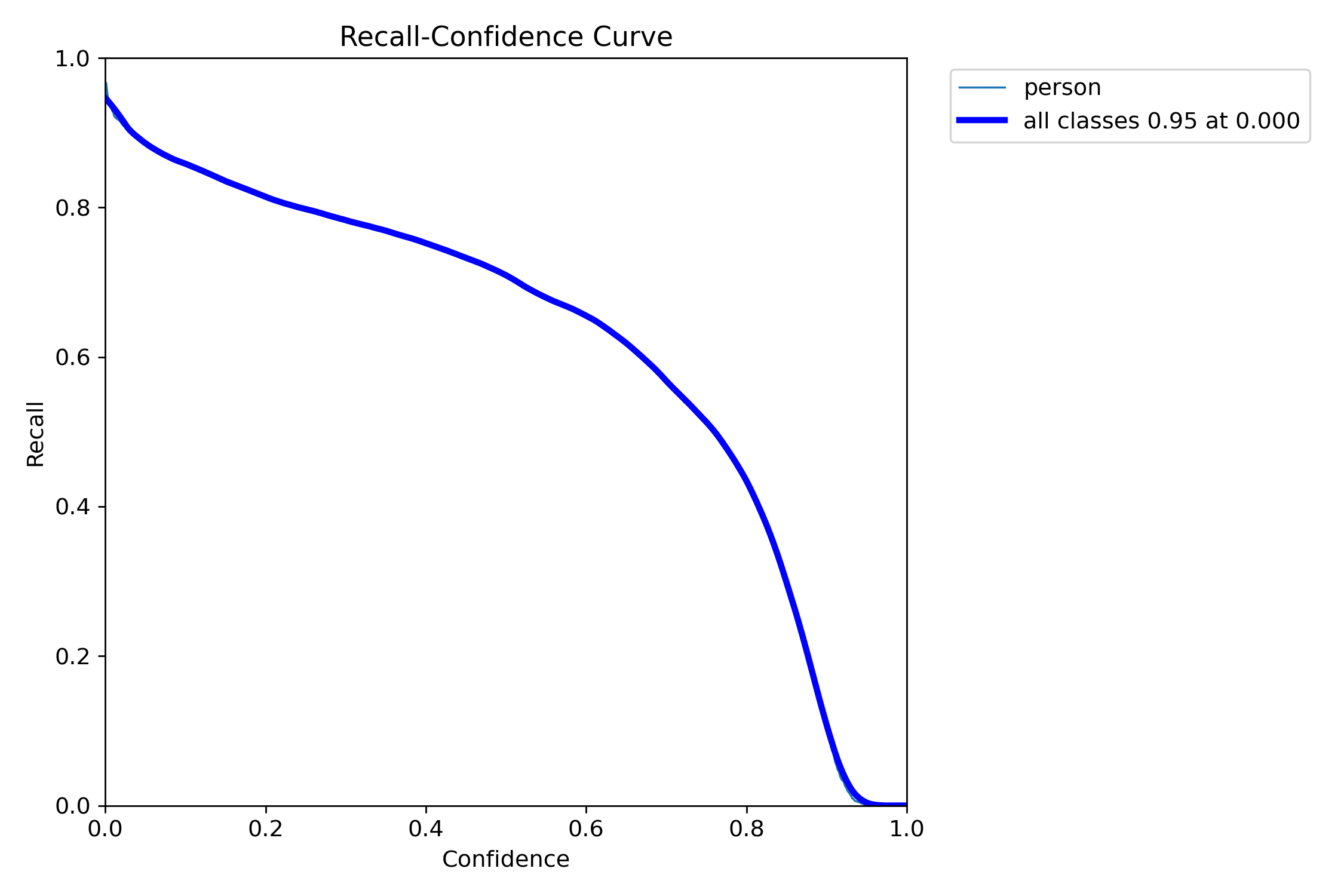

Supplement: S1 File — (ZIP) [file pone.0318578.s002.zip › suooprt information/pose/train34/BoxR_curve.png]

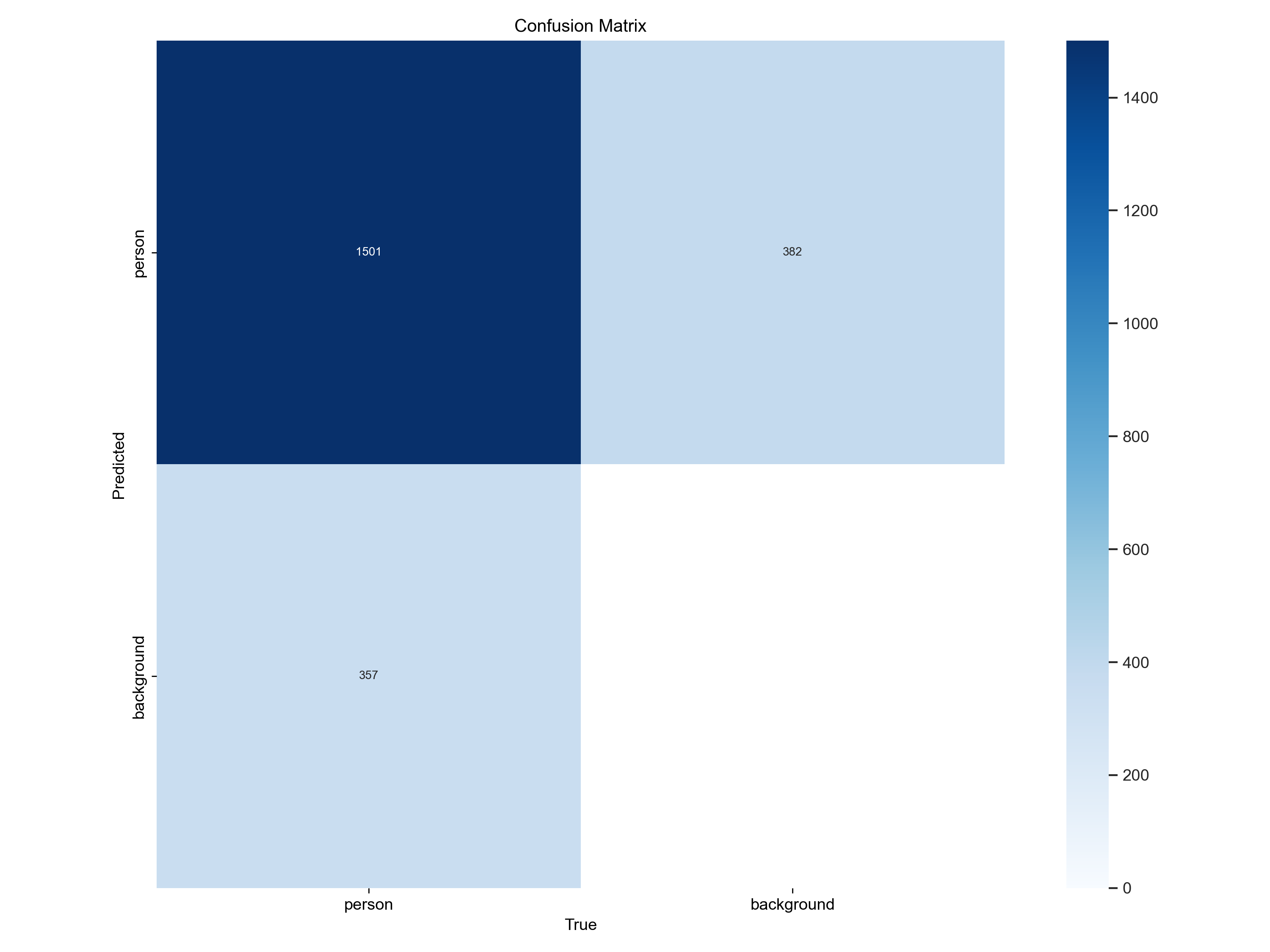

Supplement: S1 File — (ZIP) [file pone.0318578.s002.zip › suooprt information/pose/train34/confusion_matrix.png]

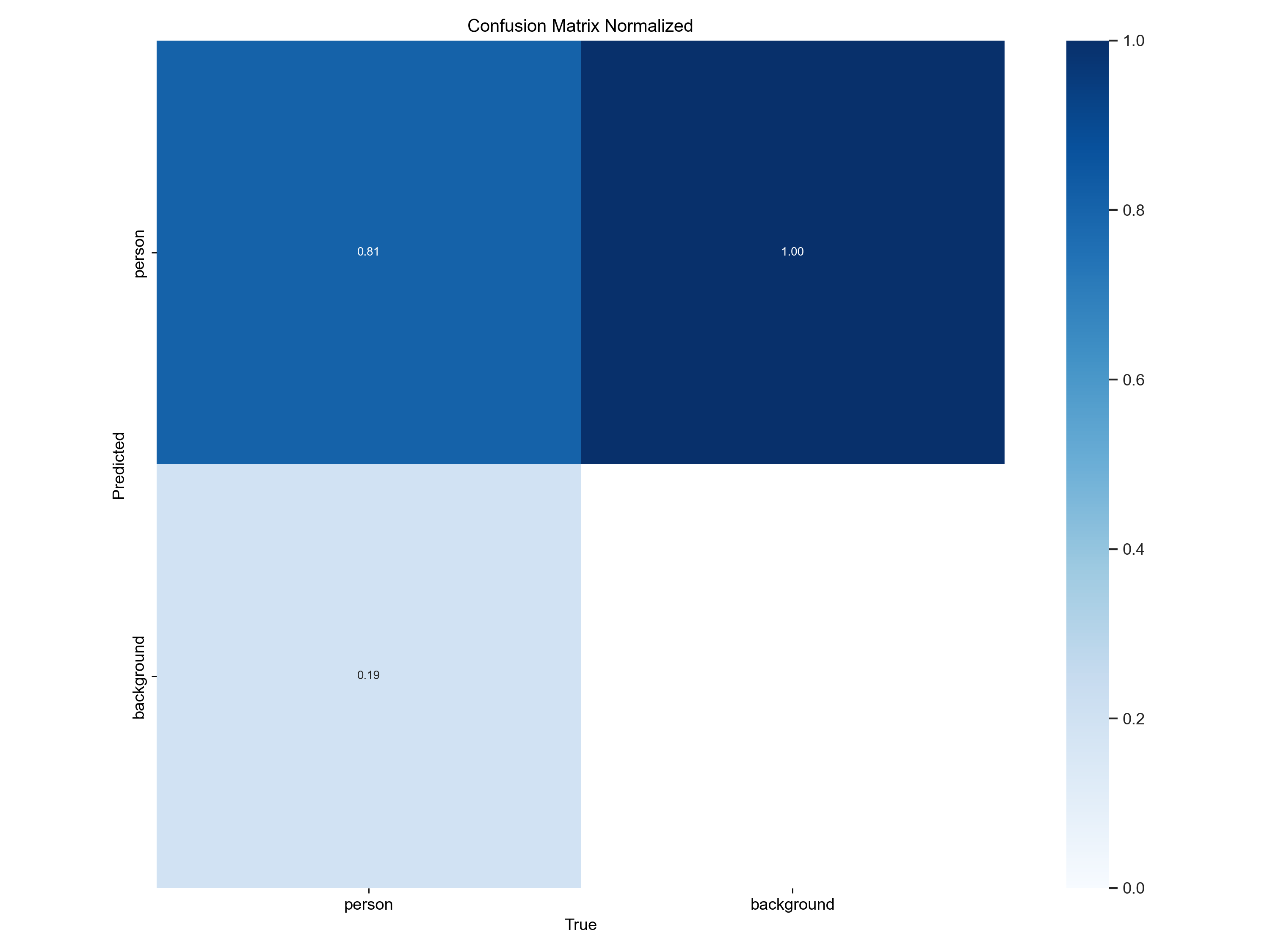

Supplement: S1 File — (ZIP) [file pone.0318578.s002.zip › suooprt information/pose/train34/confusion_matrix_normalized.png]

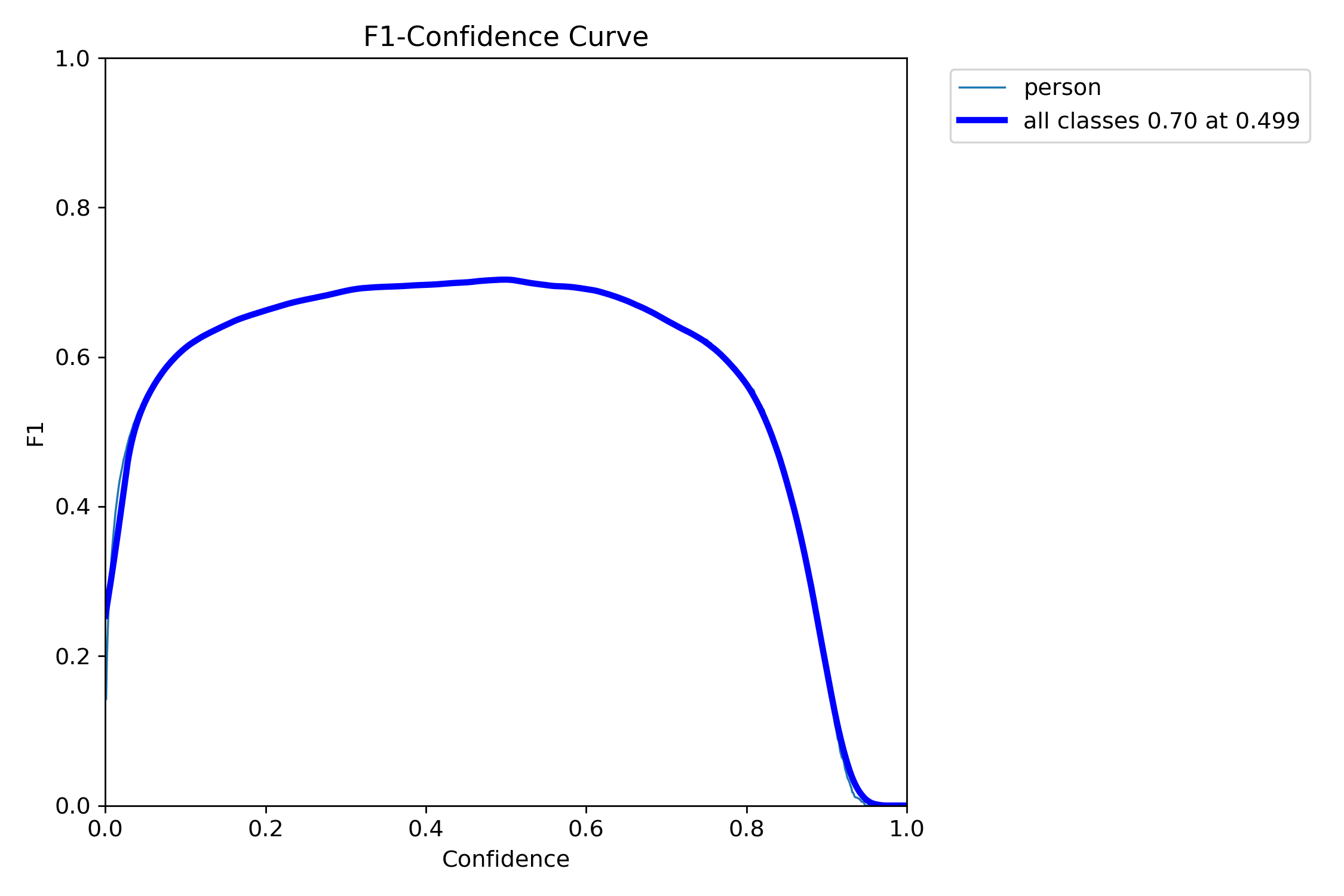

Supplement: S1 File — (ZIP) [file pone.0318578.s002.zip › suooprt information/pose/train34/PoseF1_curve.png]

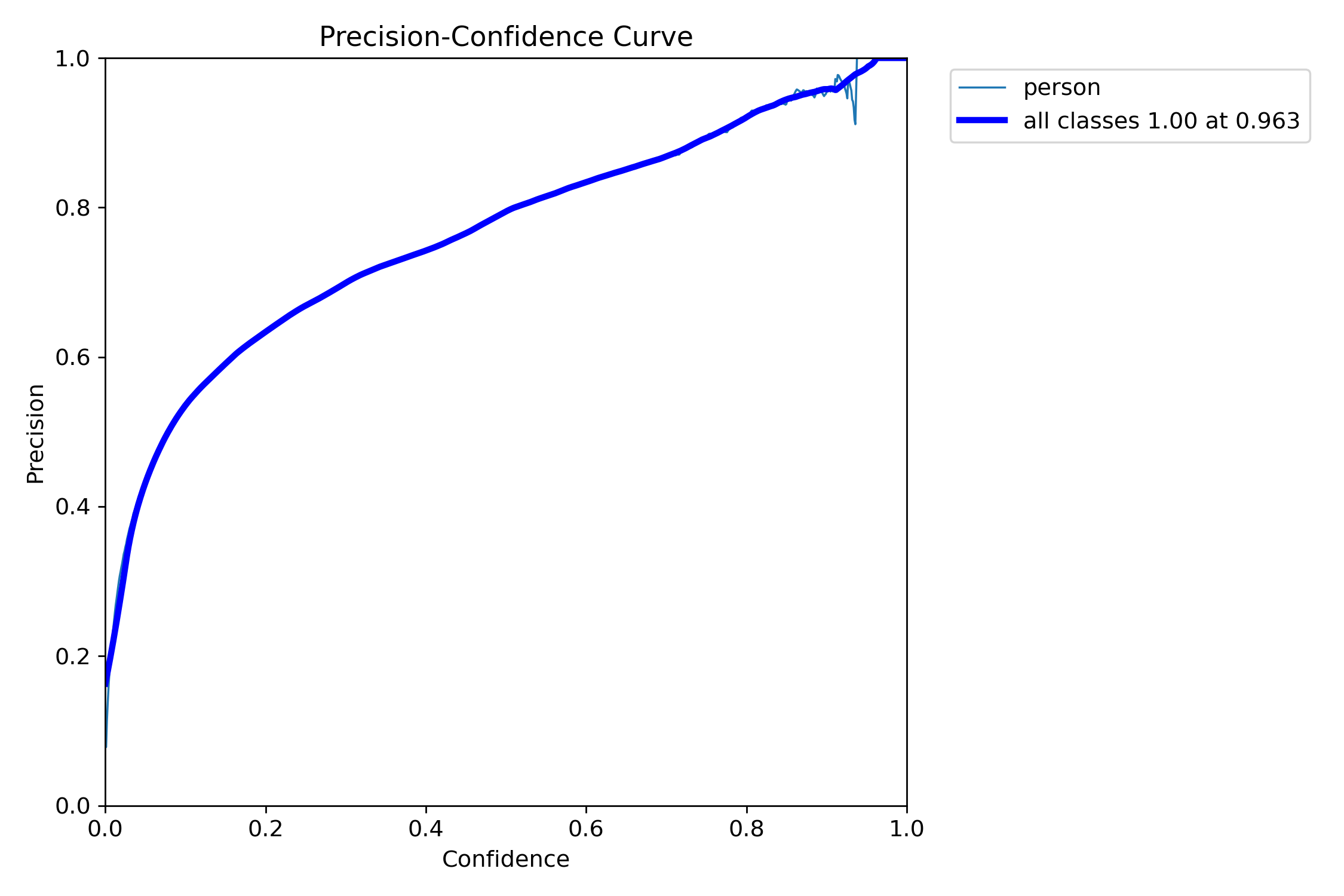

Supplement: S1 File — (ZIP) [file pone.0318578.s002.zip › suooprt information/pose/train34/PoseP_curve.png]

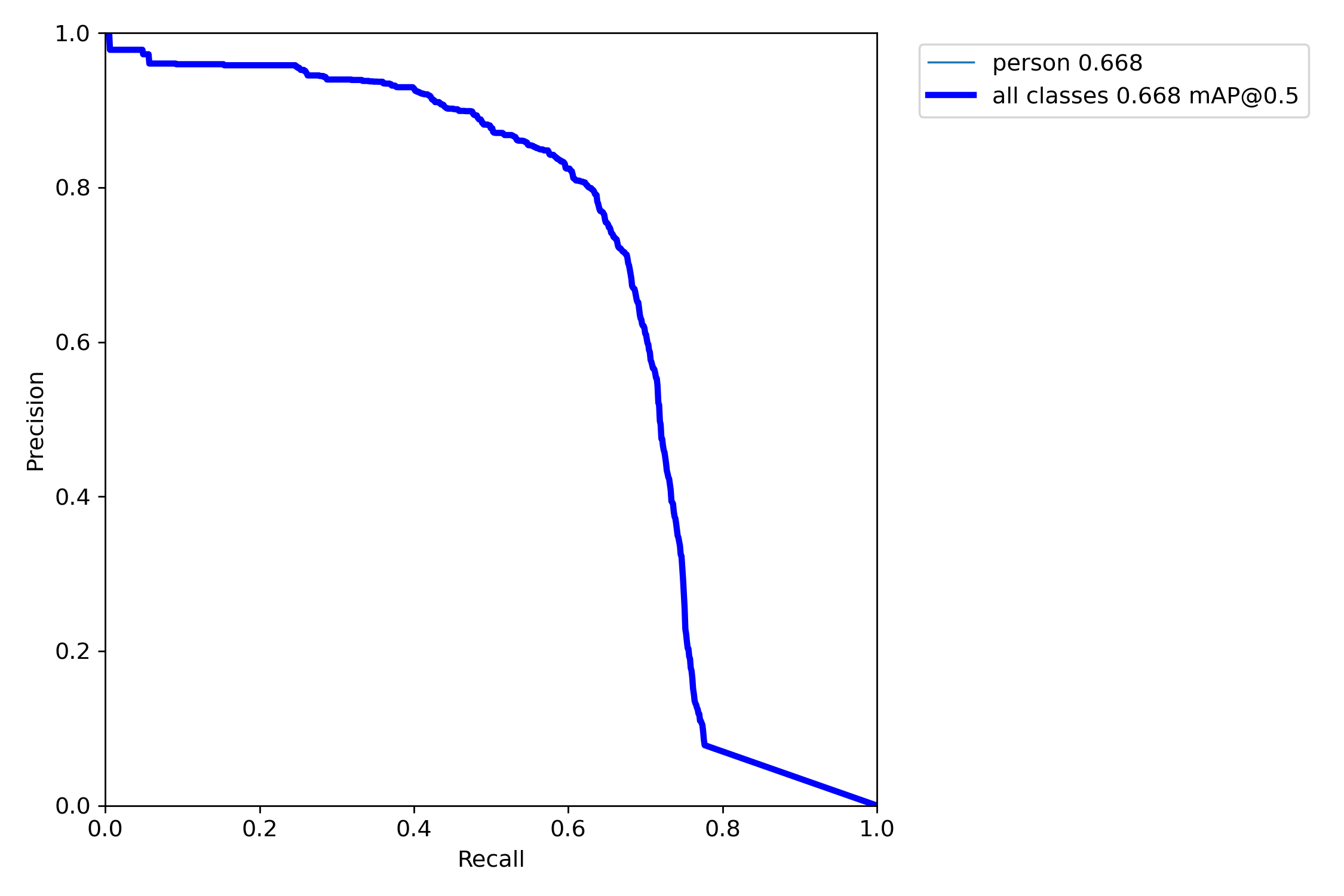

Supplement: S1 File — (ZIP) [file pone.0318578.s002.zip › suooprt information/pose/train34/PosePR_curve.png]

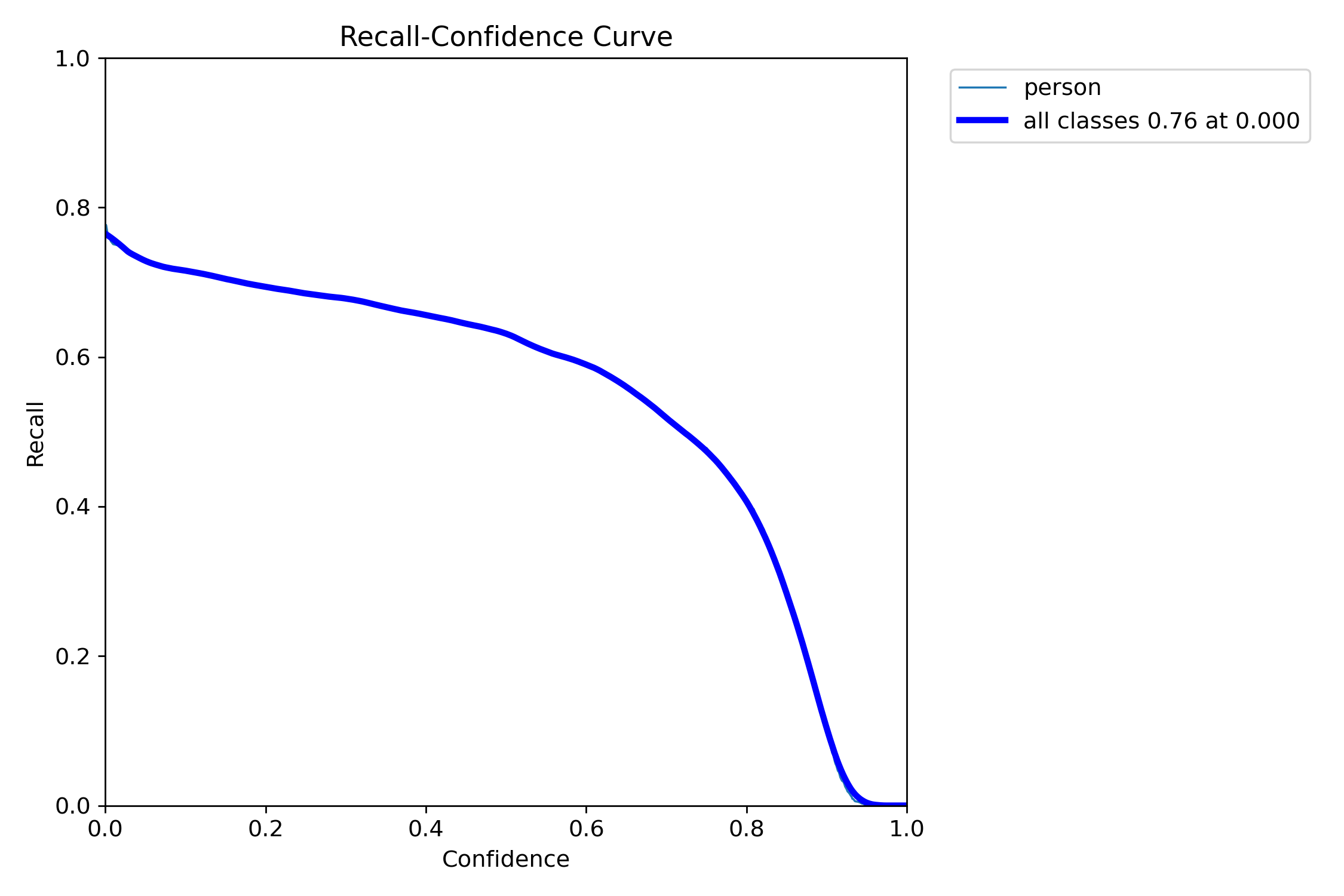

Supplement: S1 File — (ZIP) [file pone.0318578.s002.zip › suooprt information/pose/train34/PoseR_curve.png]

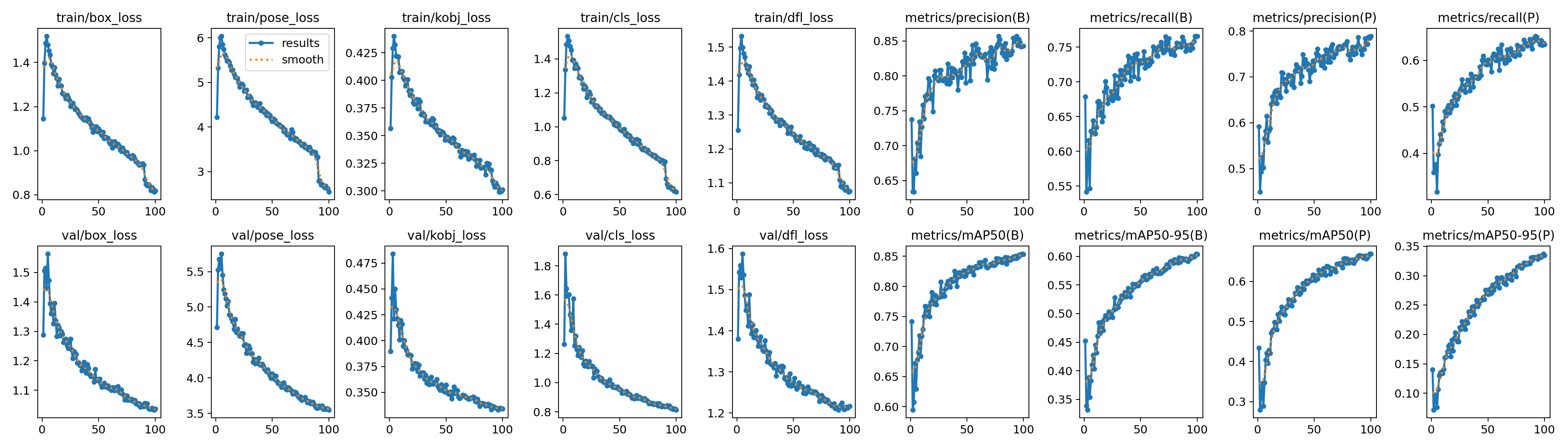

Supplement: S1 File — (ZIP) [file pone.0318578.s002.zip › suooprt information/pose/train34/results.png]

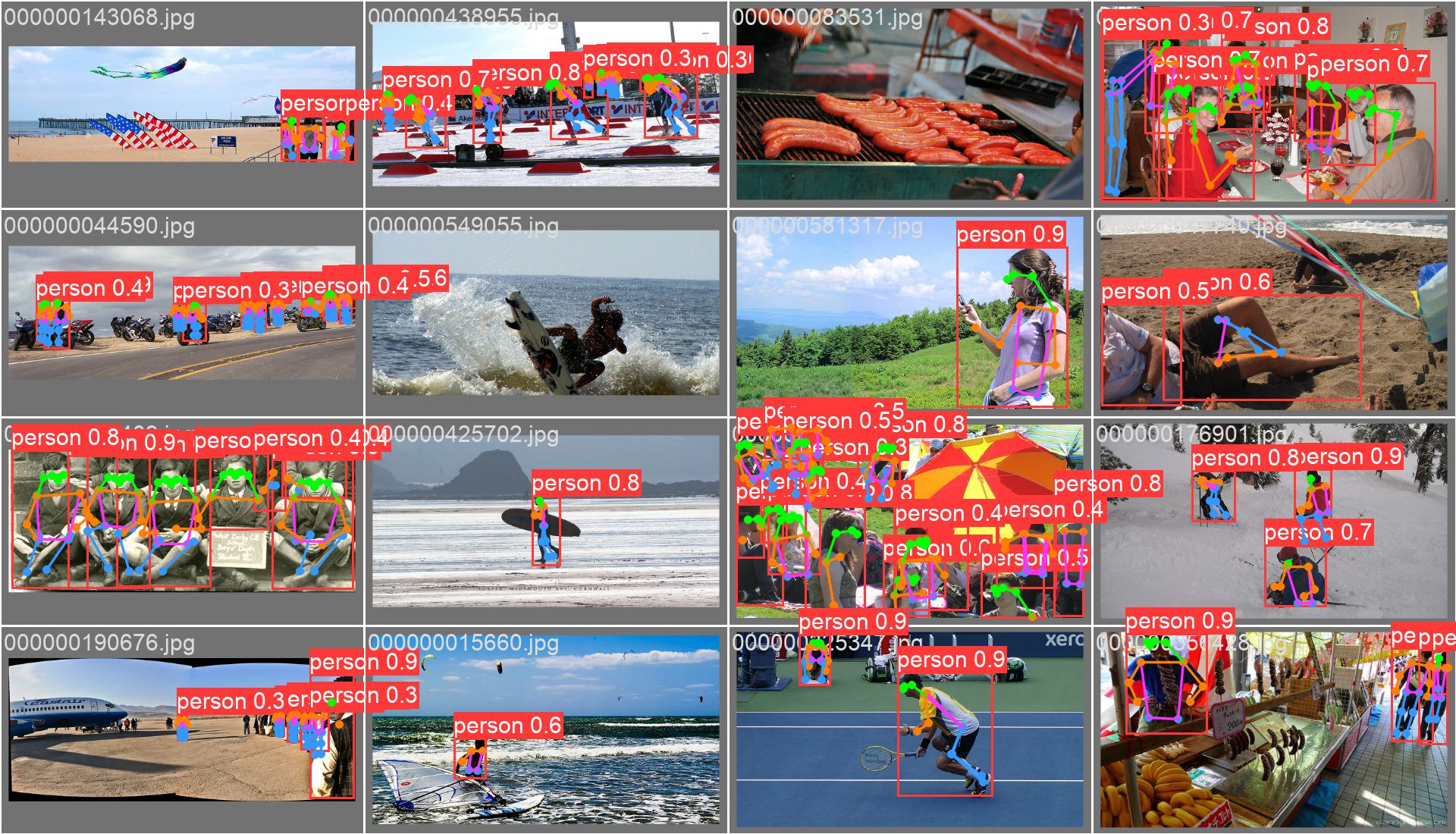

Supplement: S1 File — (ZIP) [file pone.0318578.s002.zip › suooprt information/pose/train34/val_batch0_pred.jpg]

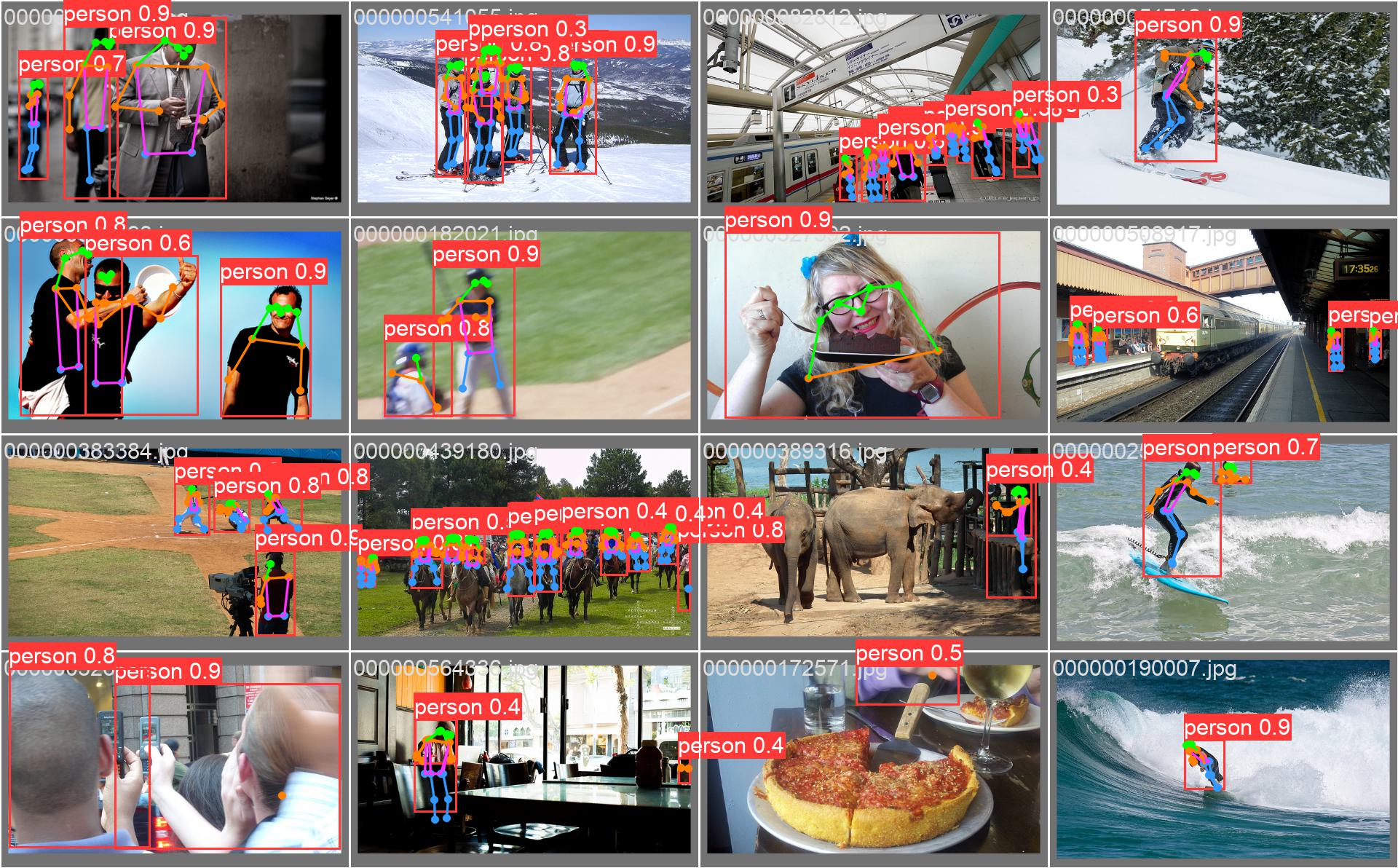

Supplement: S1 File — (ZIP) [file pone.0318578.s002.zip › suooprt information/pose/train34/val_batch1_pred.jpg]

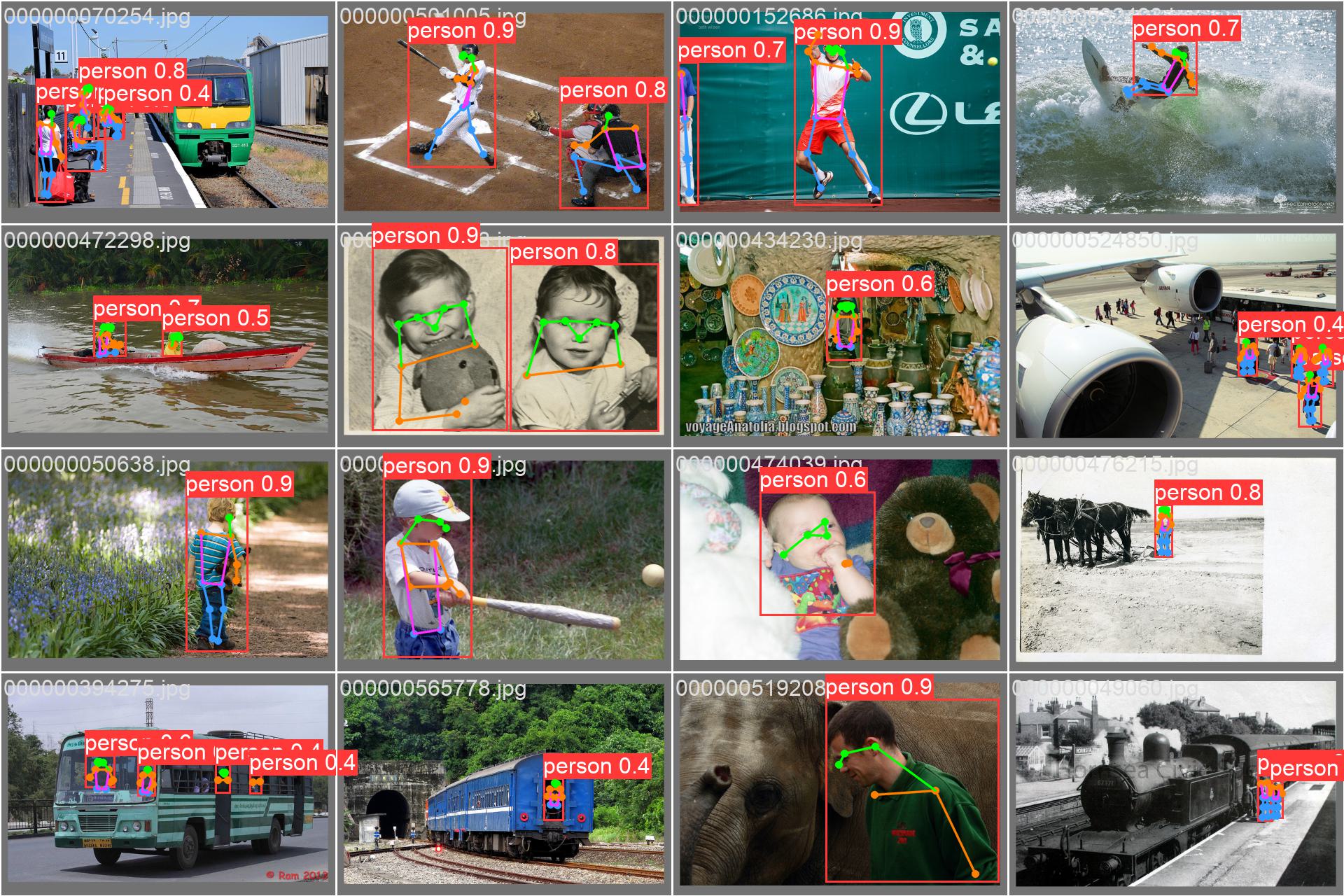

Supplement: S1 File — (ZIP) [file pone.0318578.s002.zip › suooprt information/pose/train34/val_batch2_pred.jpg]

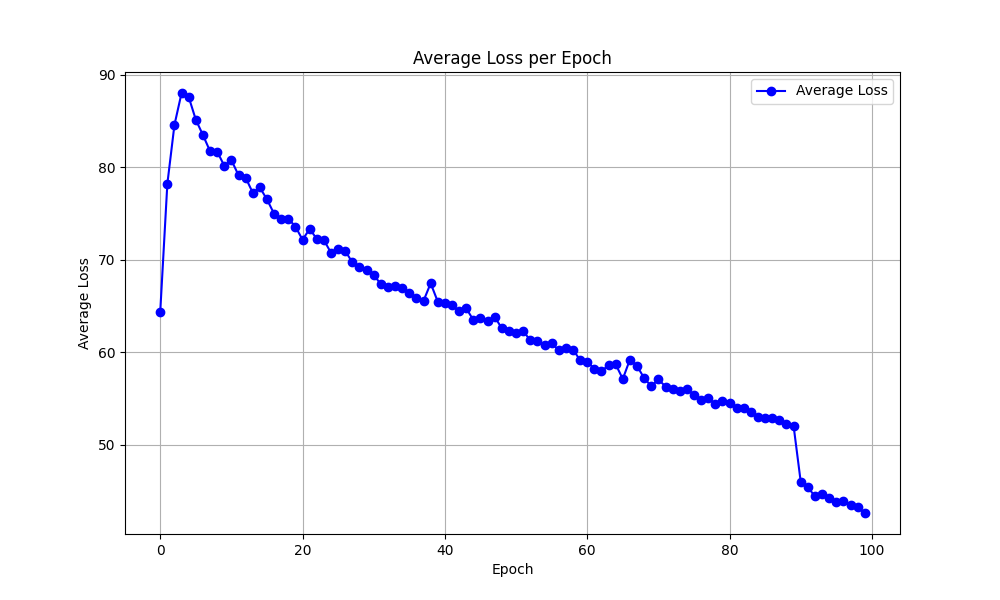

Supplement: S1 File — (ZIP) [file pone.0318578.s002.zip › suooprt information/pose/train35/avg_loss_per_epoch.png]

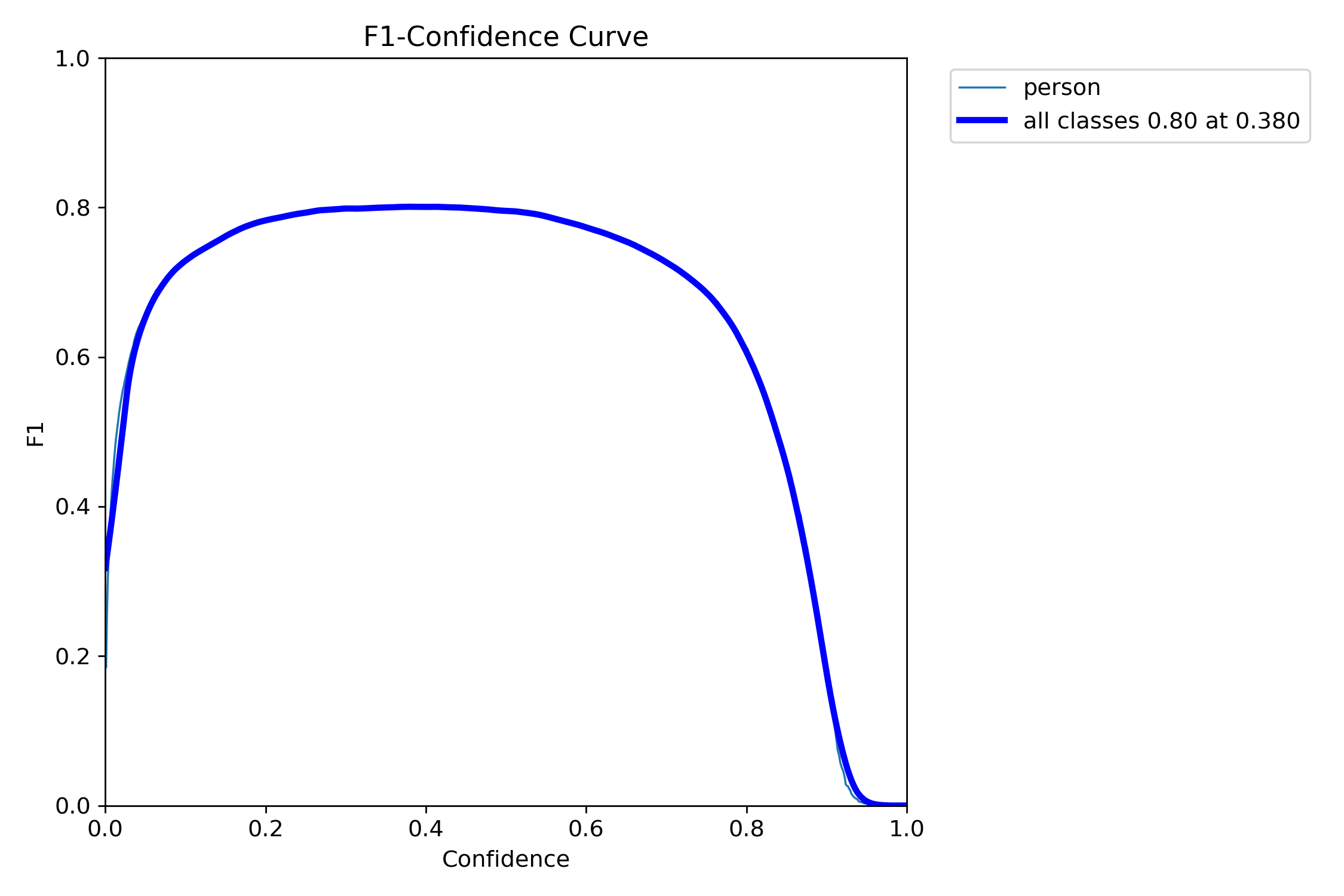

Supplement: S1 File — (ZIP) [file pone.0318578.s002.zip › suooprt information/pose/train35/BoxF1_curve.png]

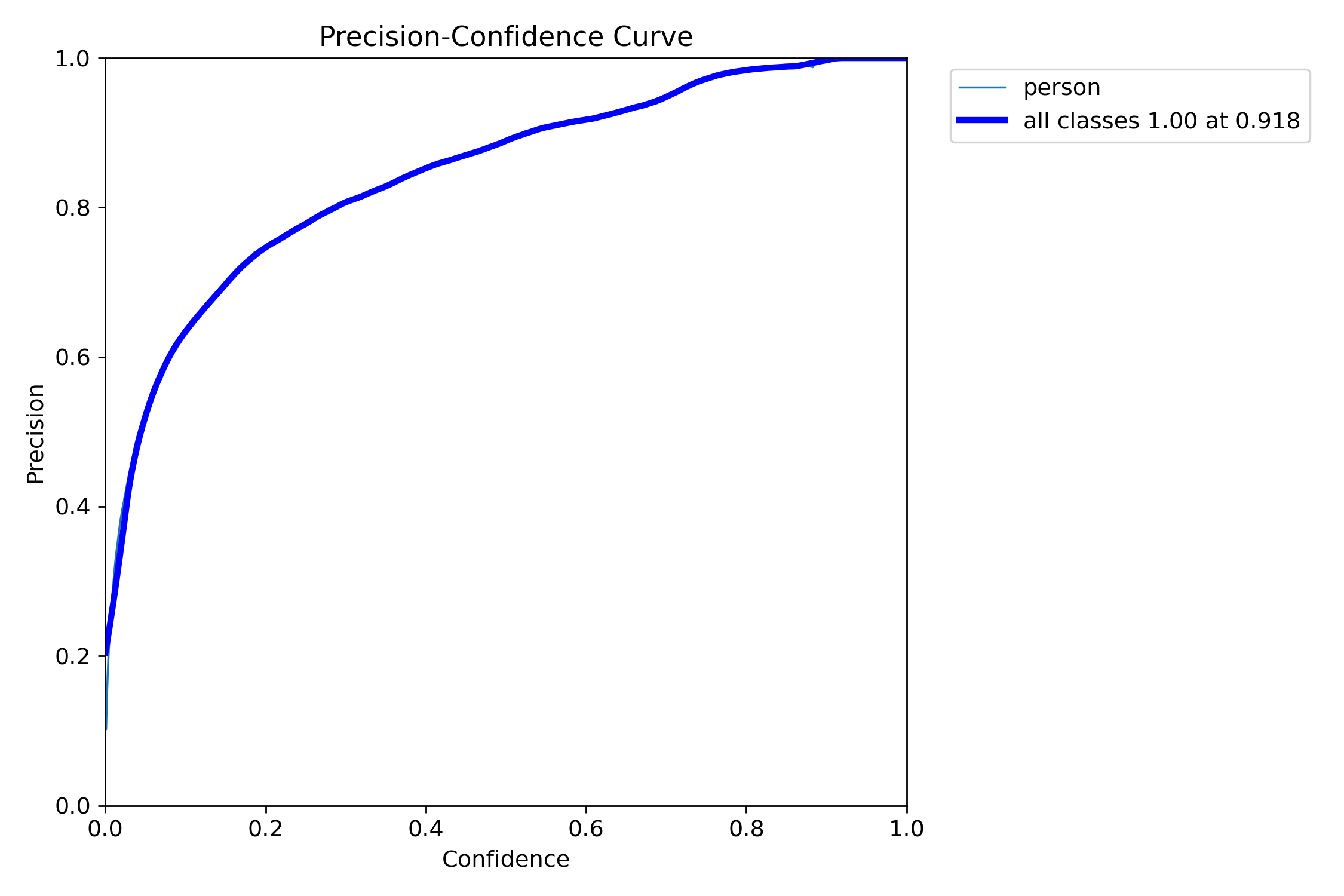

Supplement: S1 File — (ZIP) [file pone.0318578.s002.zip › suooprt information/pose/train35/BoxP_curve.png]

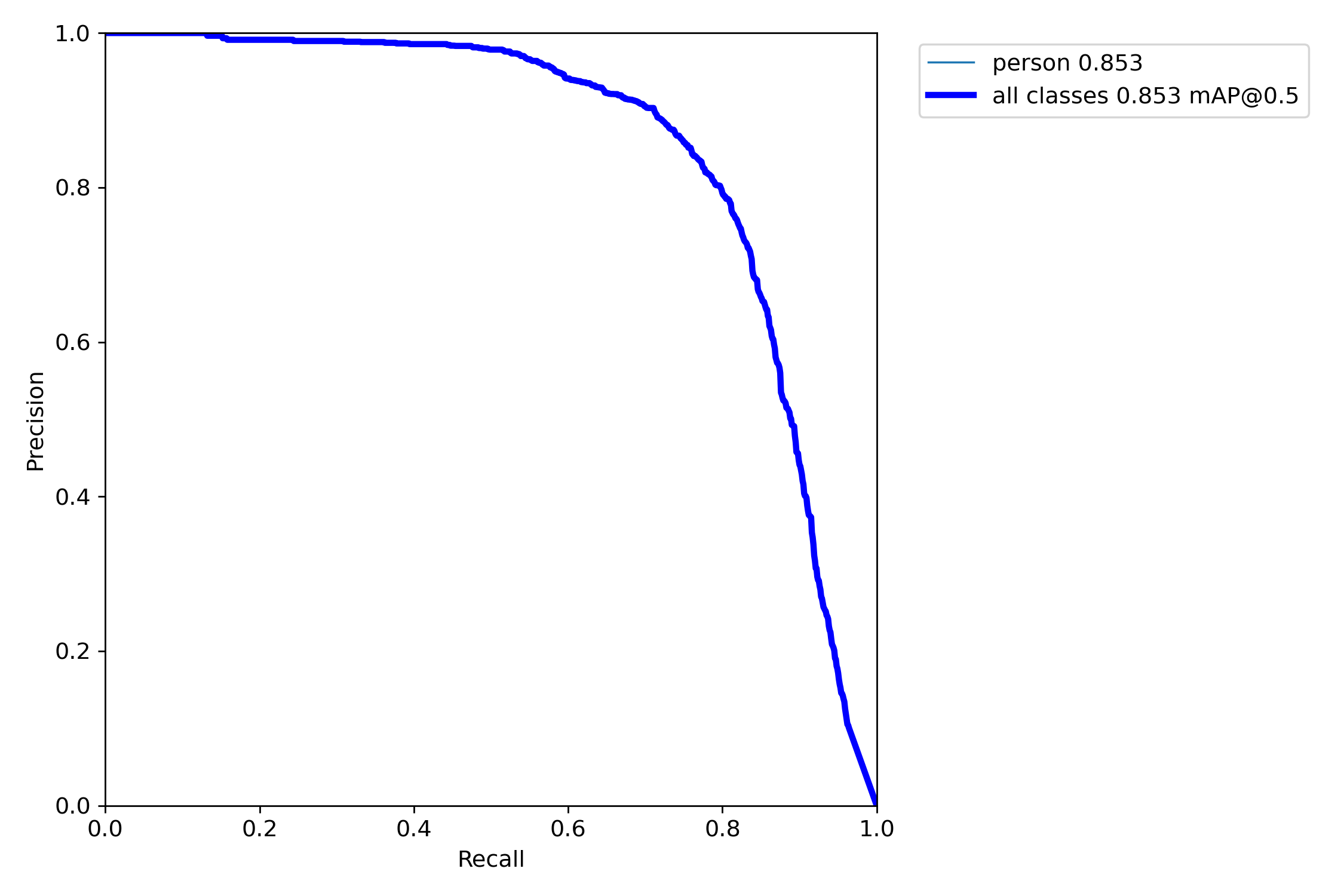

Supplement: S1 File — (ZIP) [file pone.0318578.s002.zip › suooprt information/pose/train35/BoxPR_curve.png]

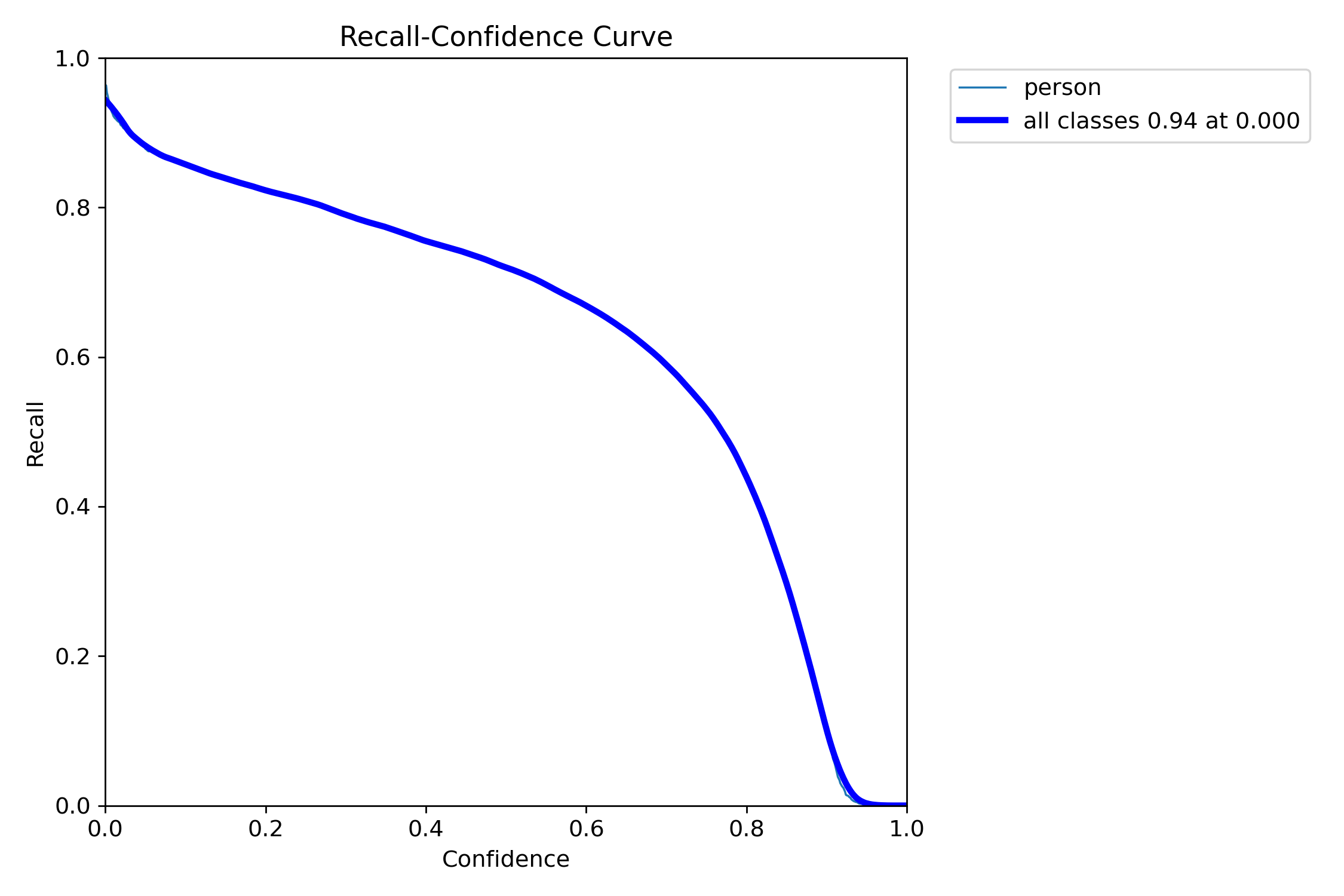

Supplement: S1 File — (ZIP) [file pone.0318578.s002.zip › suooprt information/pose/train35/BoxR_curve.png]

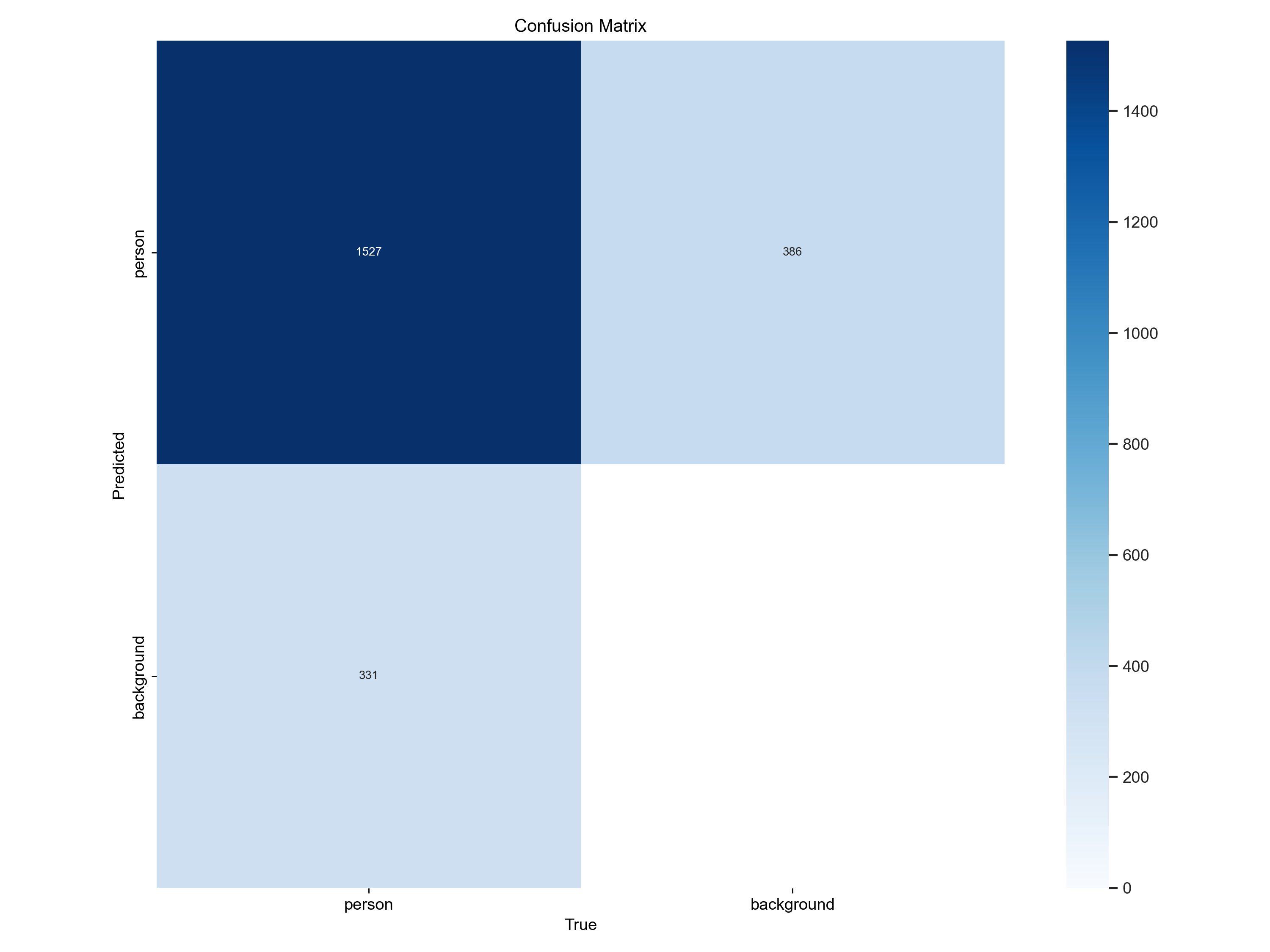

Supplement: S1 File — (ZIP) [file pone.0318578.s002.zip › suooprt information/pose/train35/confusion_matrix.png]

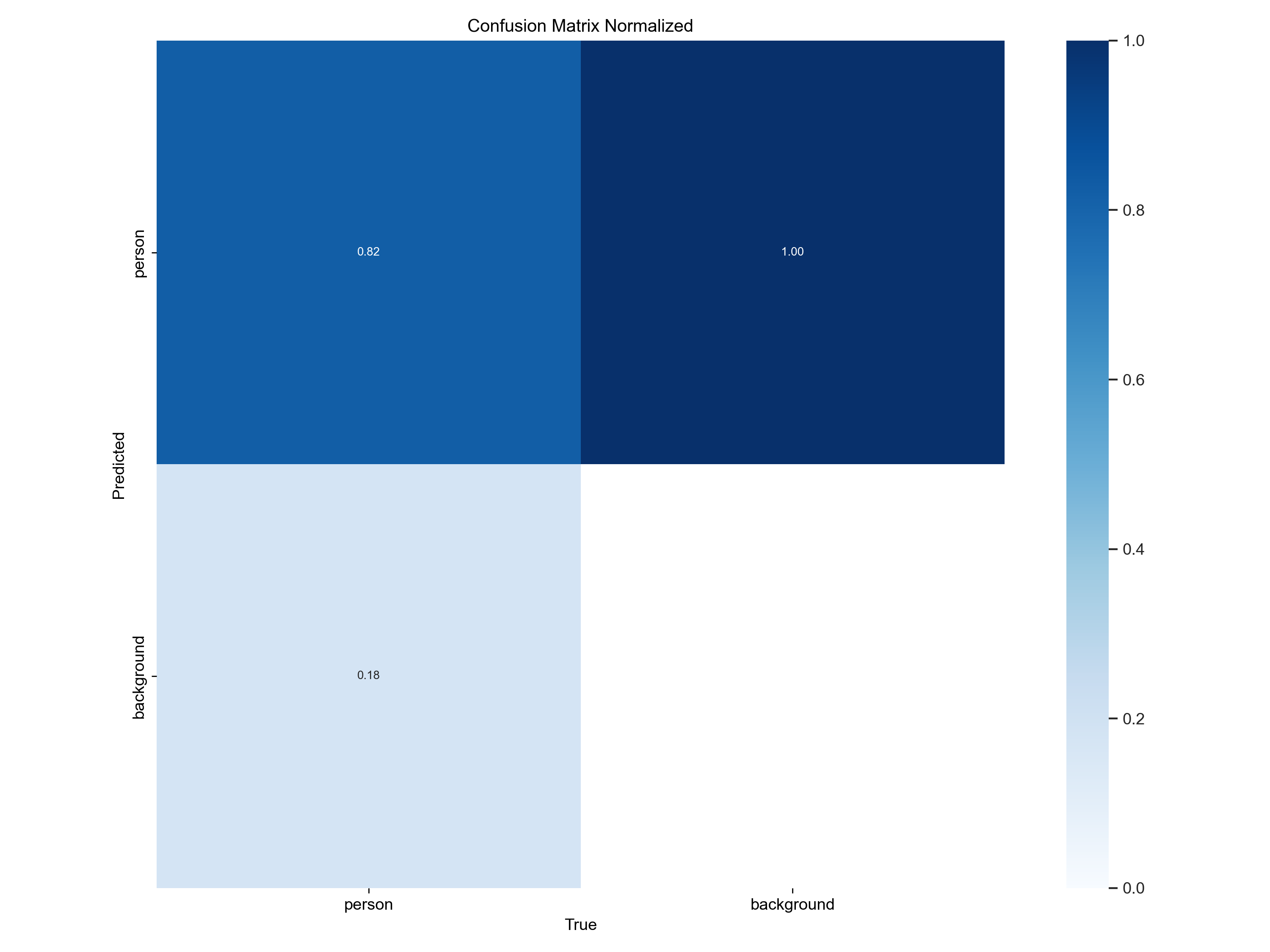

Supplement: S1 File — (ZIP) [file pone.0318578.s002.zip › suooprt information/pose/train35/confusion_matrix_normalized.png]

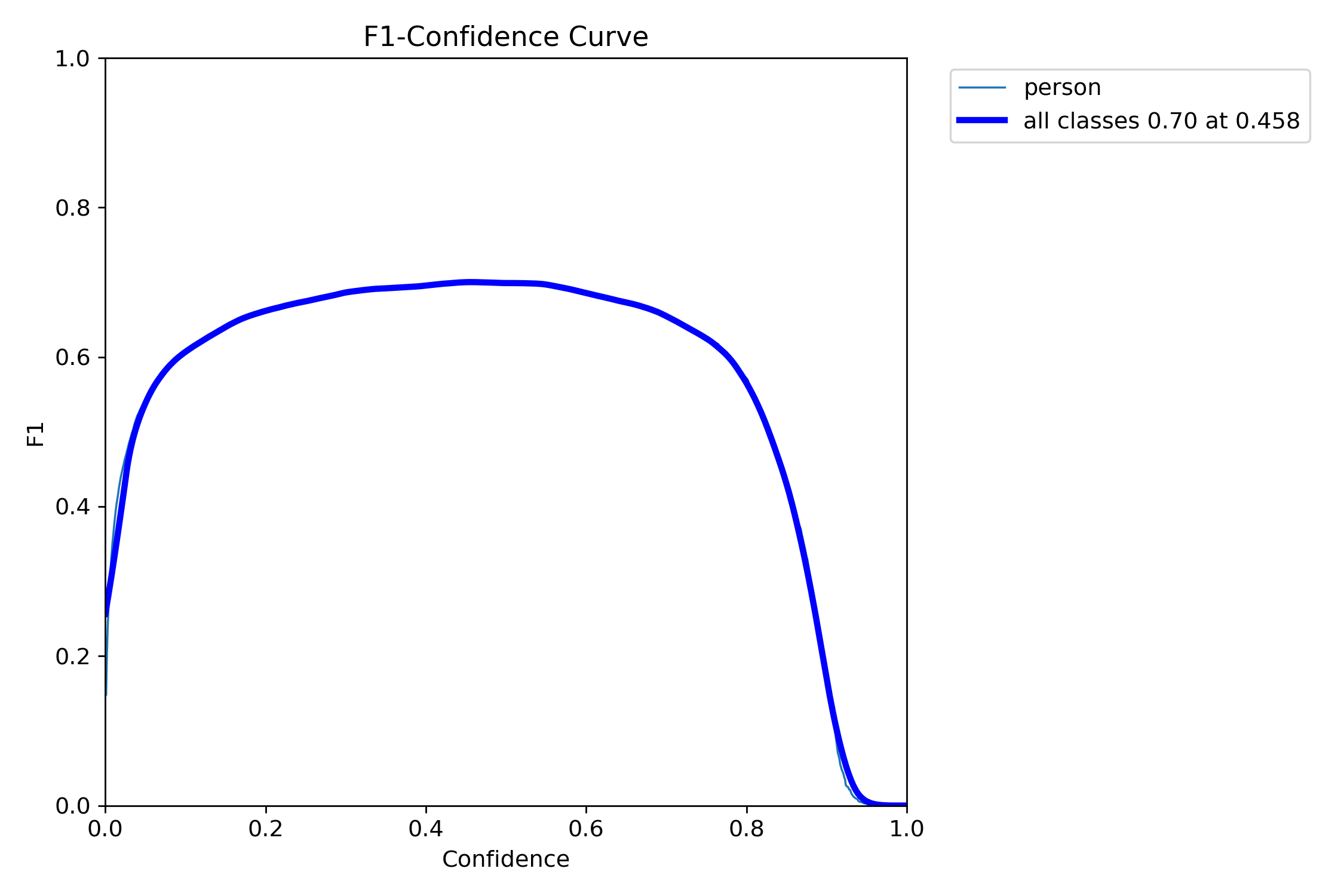

Supplement: S1 File — (ZIP) [file pone.0318578.s002.zip › suooprt information/pose/train35/PoseF1_curve.png]

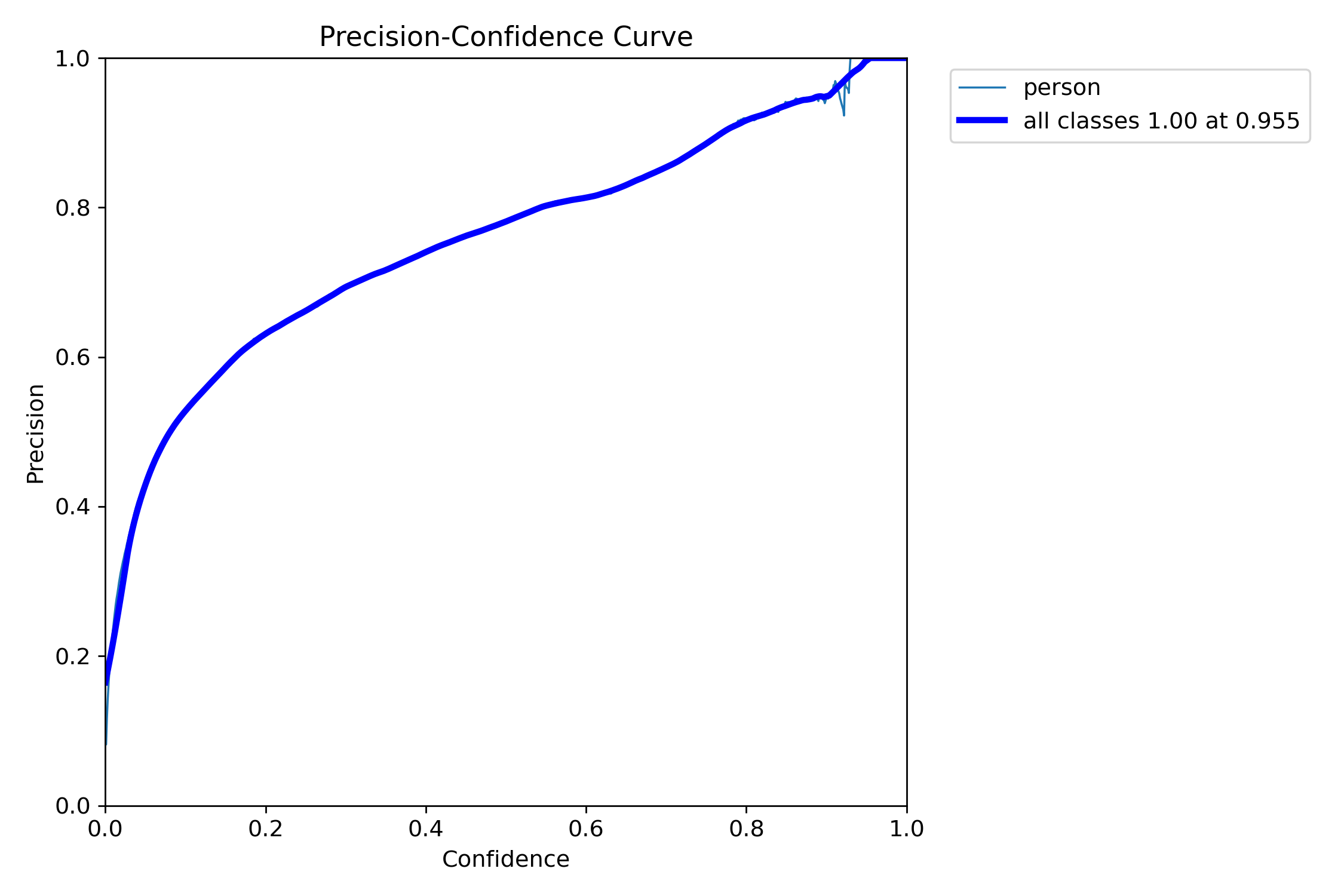

Supplement: S1 File — (ZIP) [file pone.0318578.s002.zip › suooprt information/pose/train35/PoseP_curve.png]

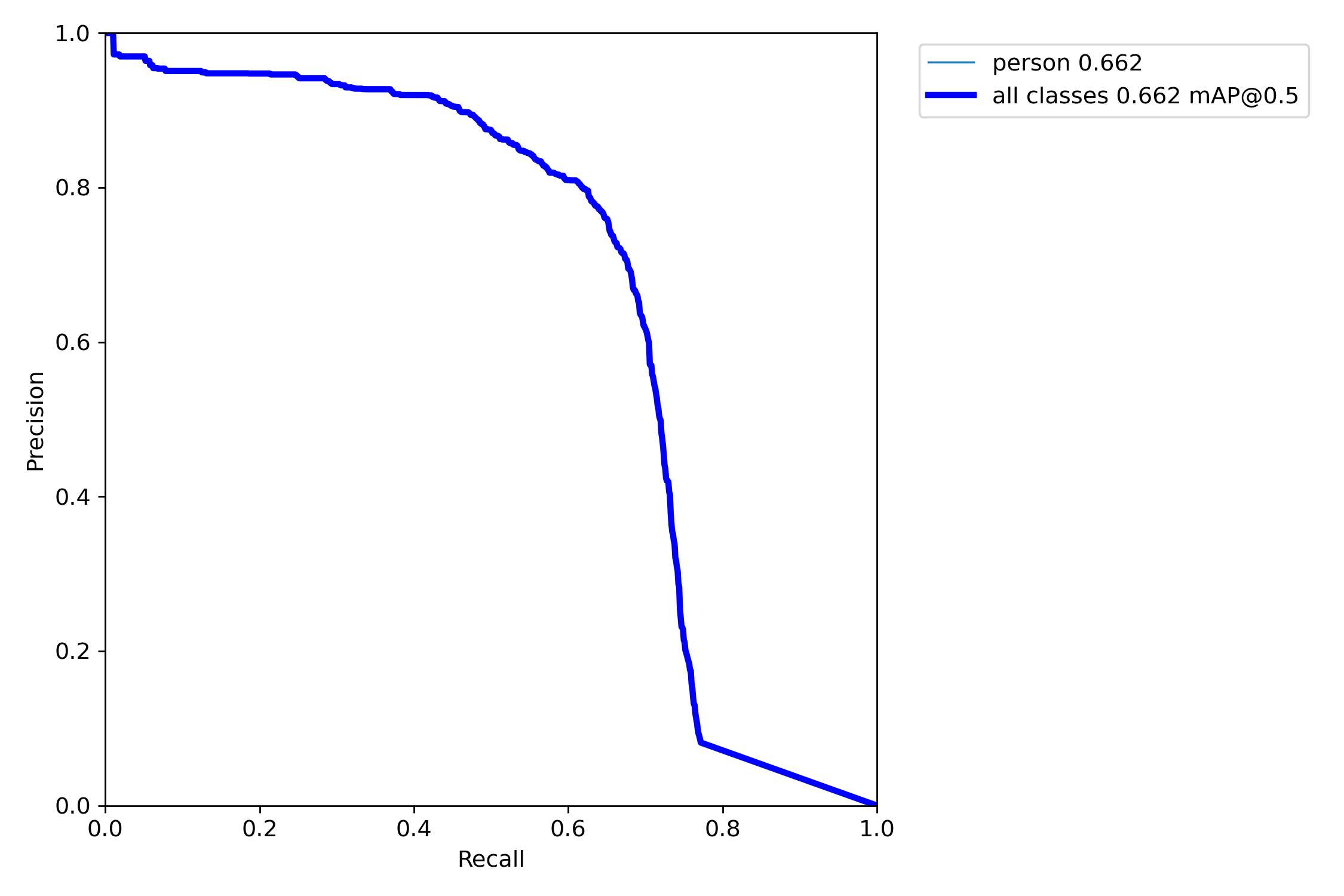

Supplement: S1 File — (ZIP) [file pone.0318578.s002.zip › suooprt information/pose/train35/PosePR_curve.png]

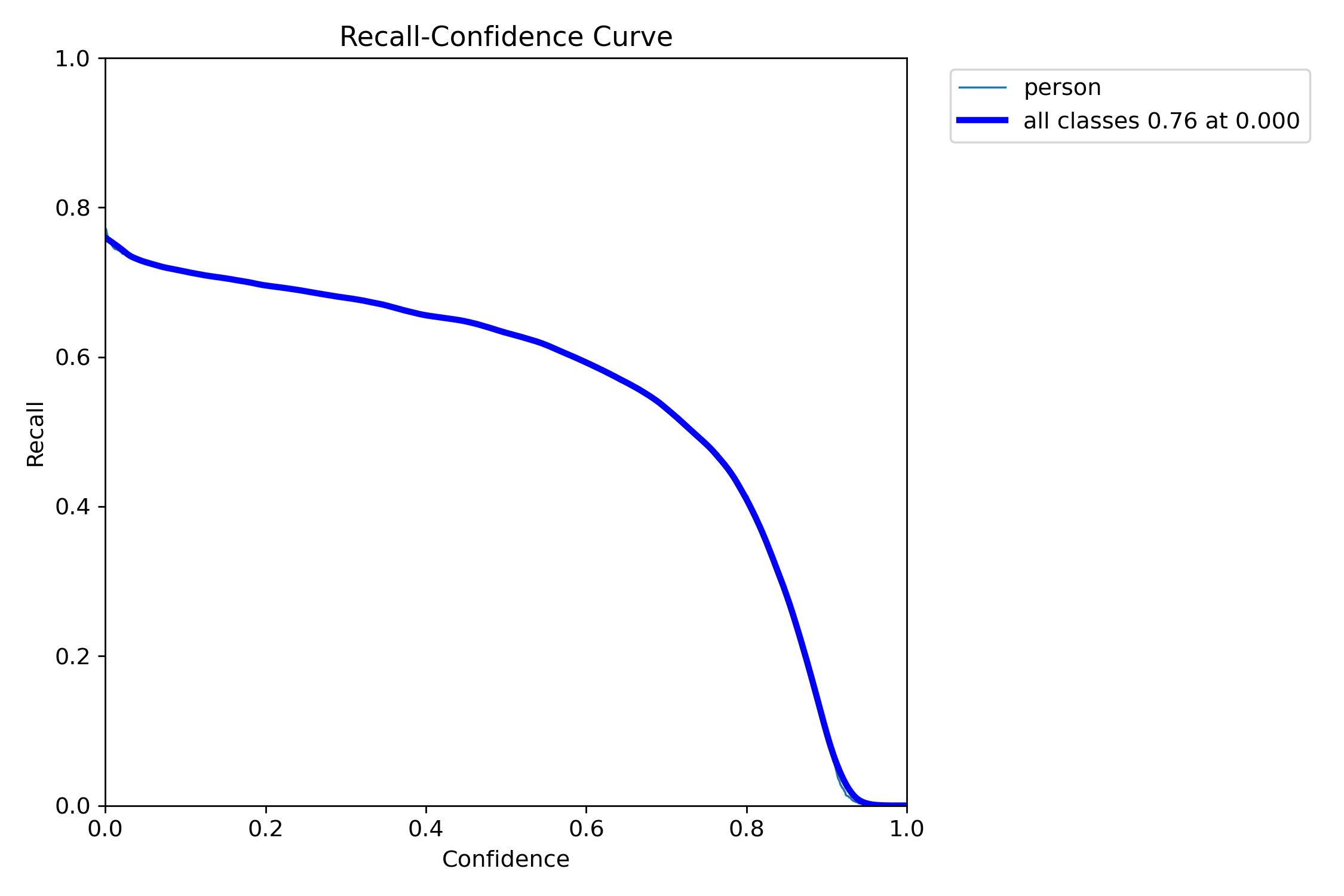

Supplement: S1 File — (ZIP) [file pone.0318578.s002.zip › suooprt information/pose/train35/PoseR_curve.png]

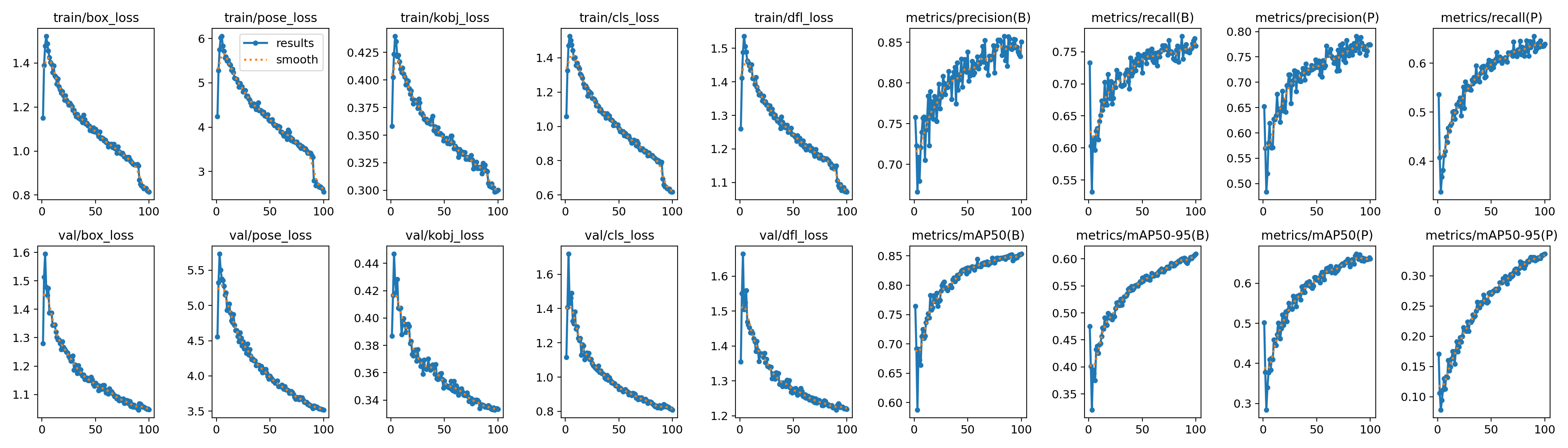

Supplement: S1 File — (ZIP) [file pone.0318578.s002.zip › suooprt information/pose/train35/results.png]

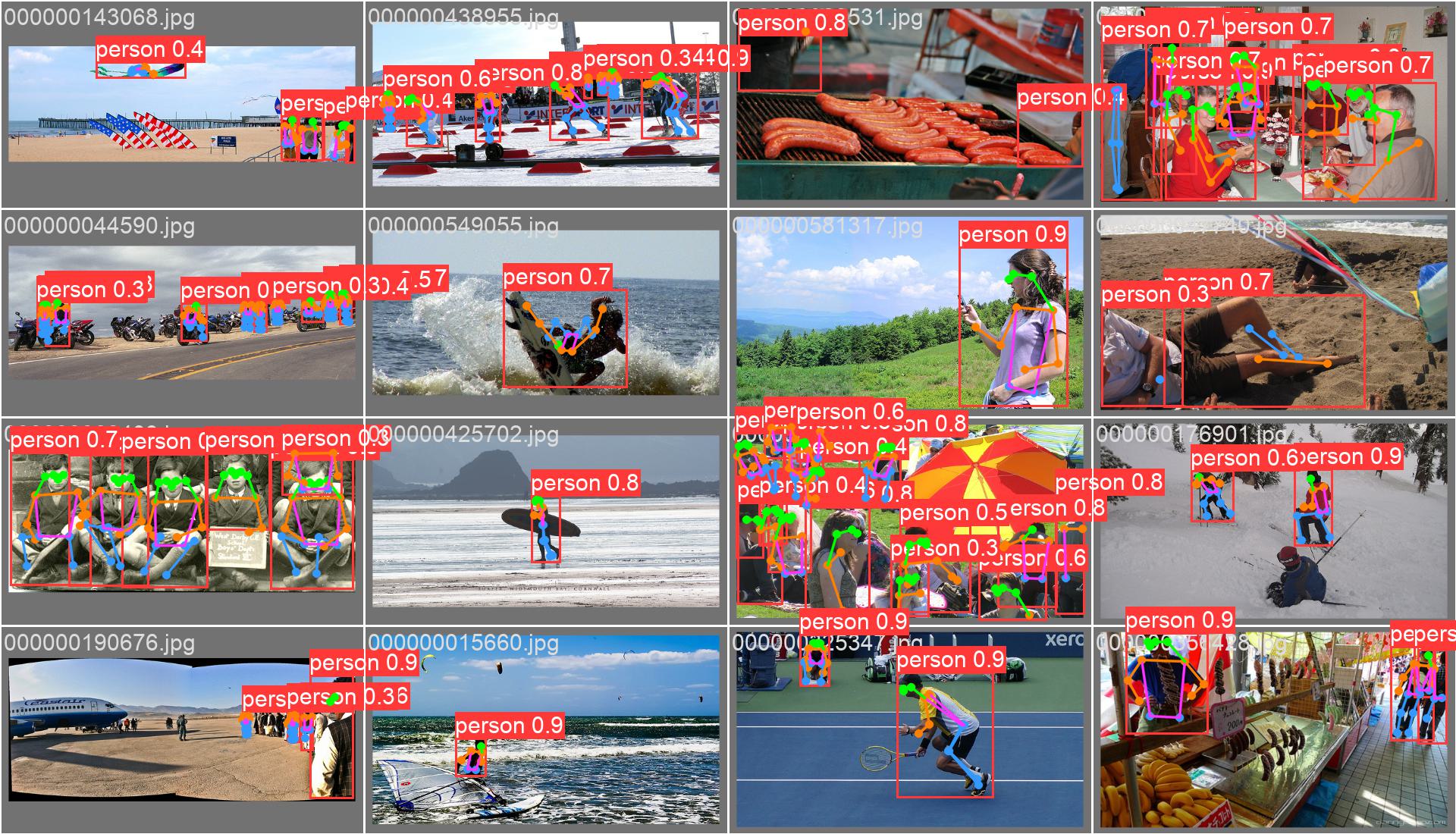

Supplement: S1 File — (ZIP) [file pone.0318578.s002.zip › suooprt information/pose/train35/val_batch0_pred.jpg]

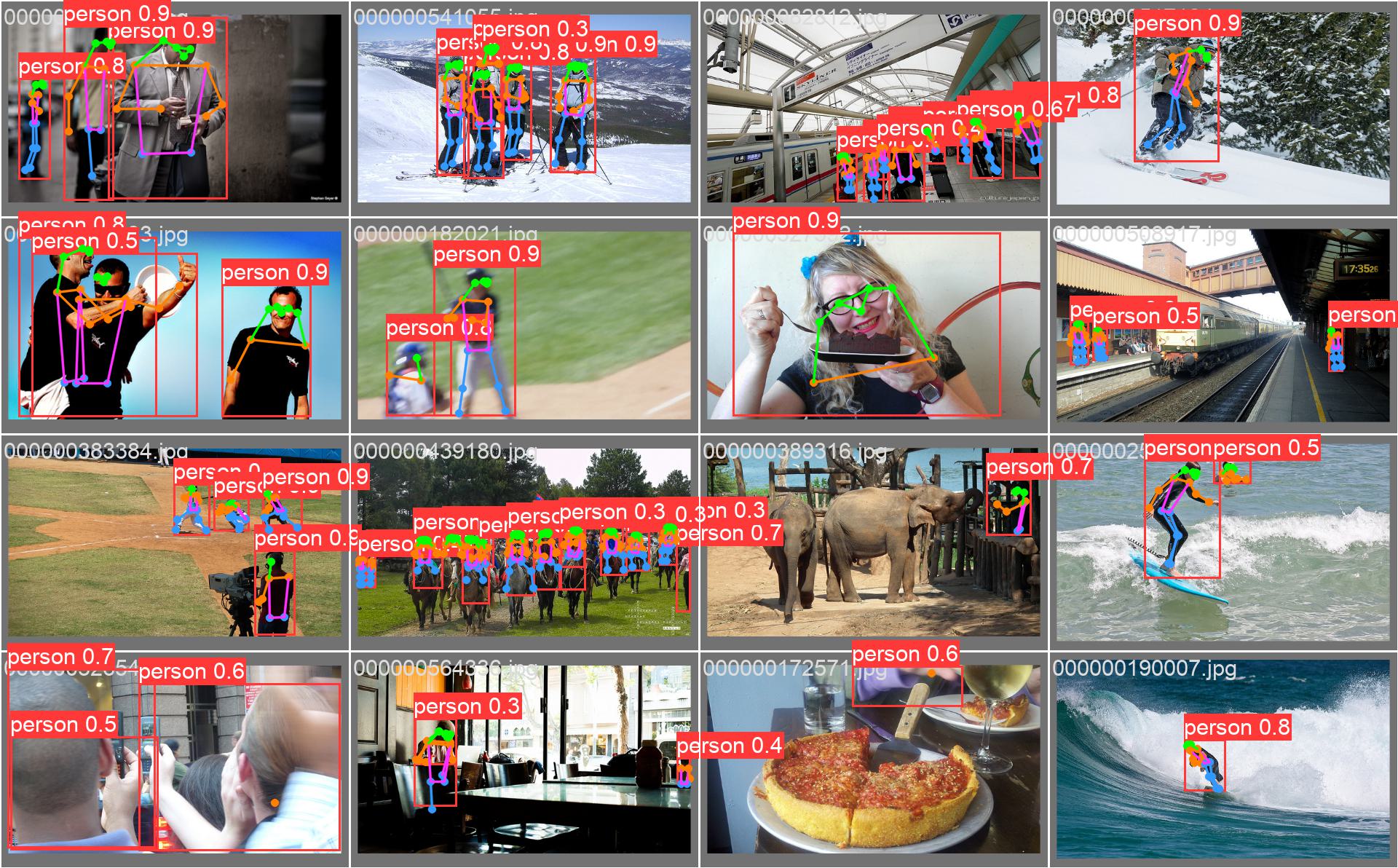

Supplement: S1 File — (ZIP) [file pone.0318578.s002.zip › suooprt information/pose/train35/val_batch1_pred.jpg]

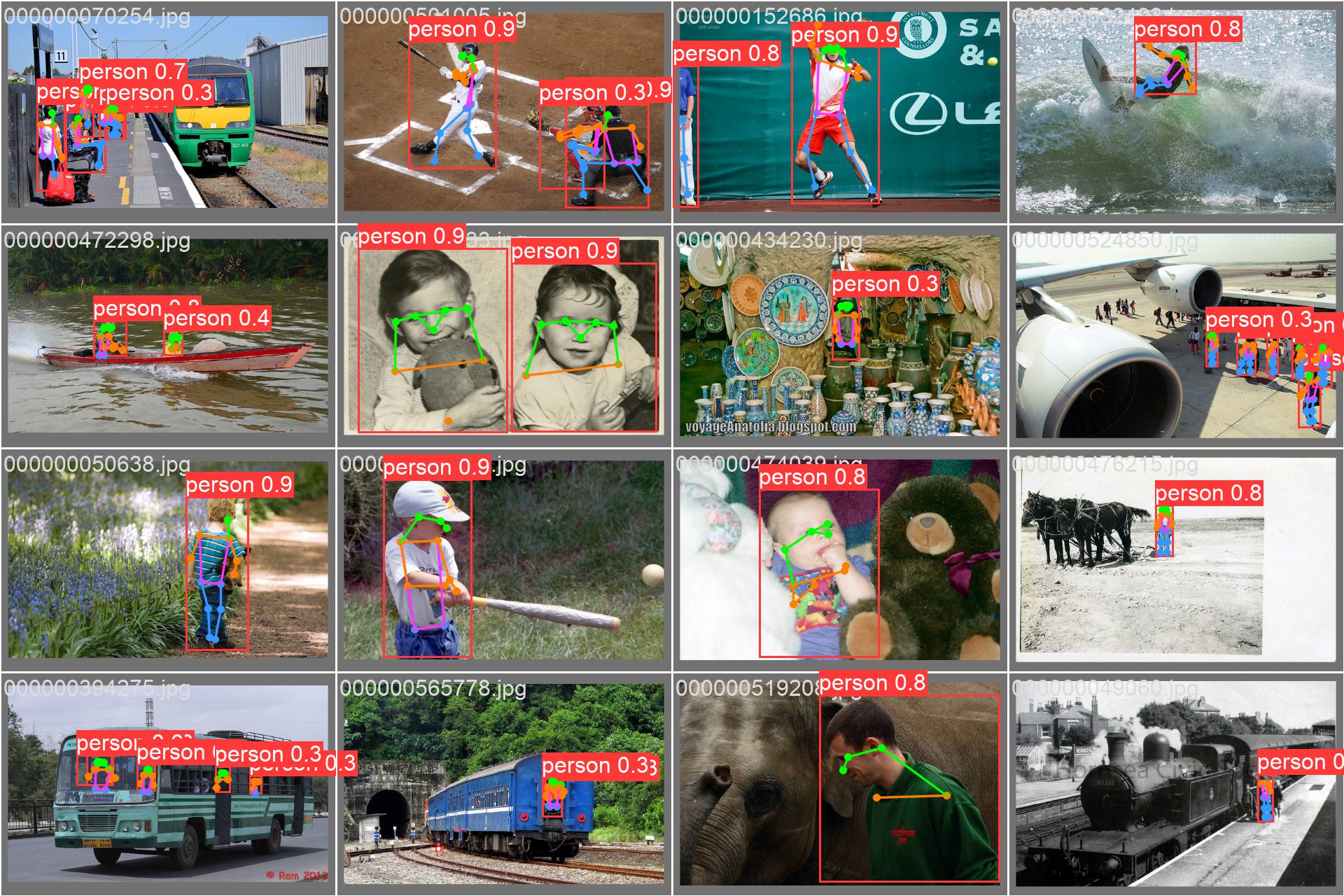

Supplement: S1 File — (ZIP) [file pone.0318578.s002.zip › suooprt information/pose/train35/val_batch2_pred.jpg]
