# Supplementary figures and images for: Enhanced human pose estimation using YOLOv8 with Integrated SimDLKA attention mechanism and DCIOU loss function: Analysis of human body behavior and posture (part 1 of 2)
Source: PLoS One. 2025 May 7;20(5):e0318578. doi: 10.1371/journal.pone.0318578 (PMC12057905; doi:10.1371/journal.pone.0318578)

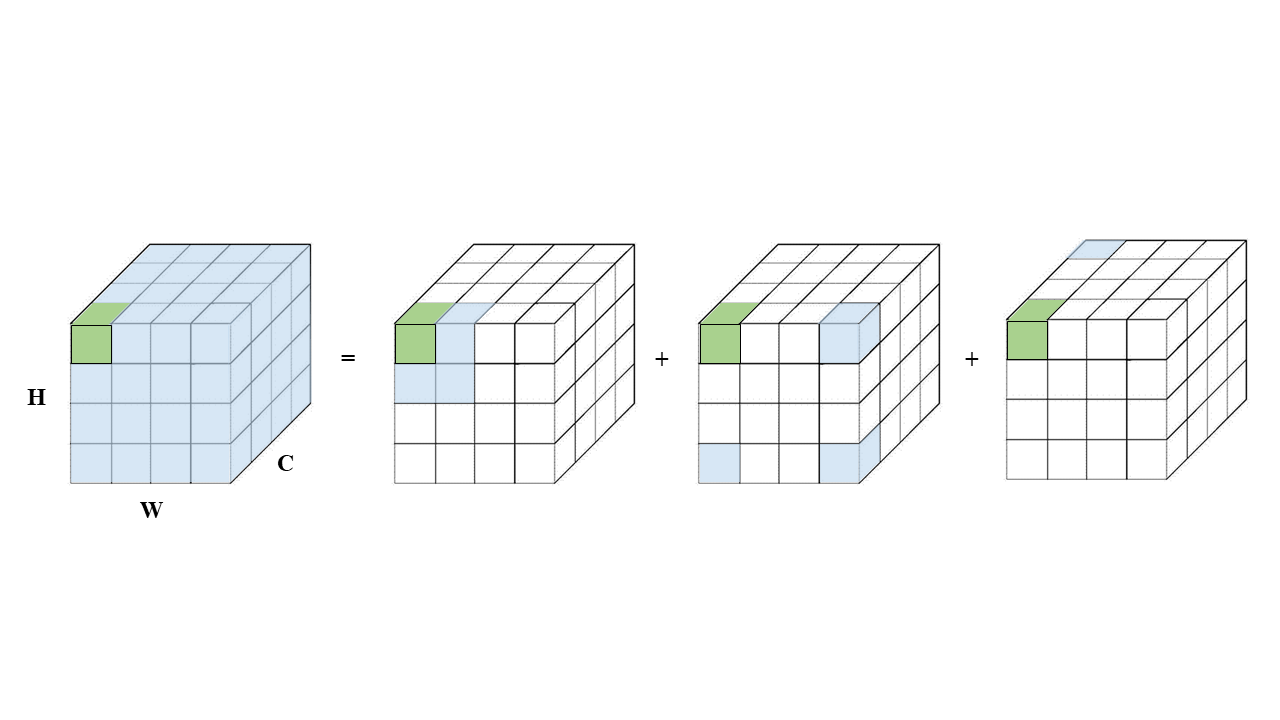

Supplement: S1 File — (ZIP) [file pone.0318578.s002.zip › suooprt information/images/Fig 2.tif]

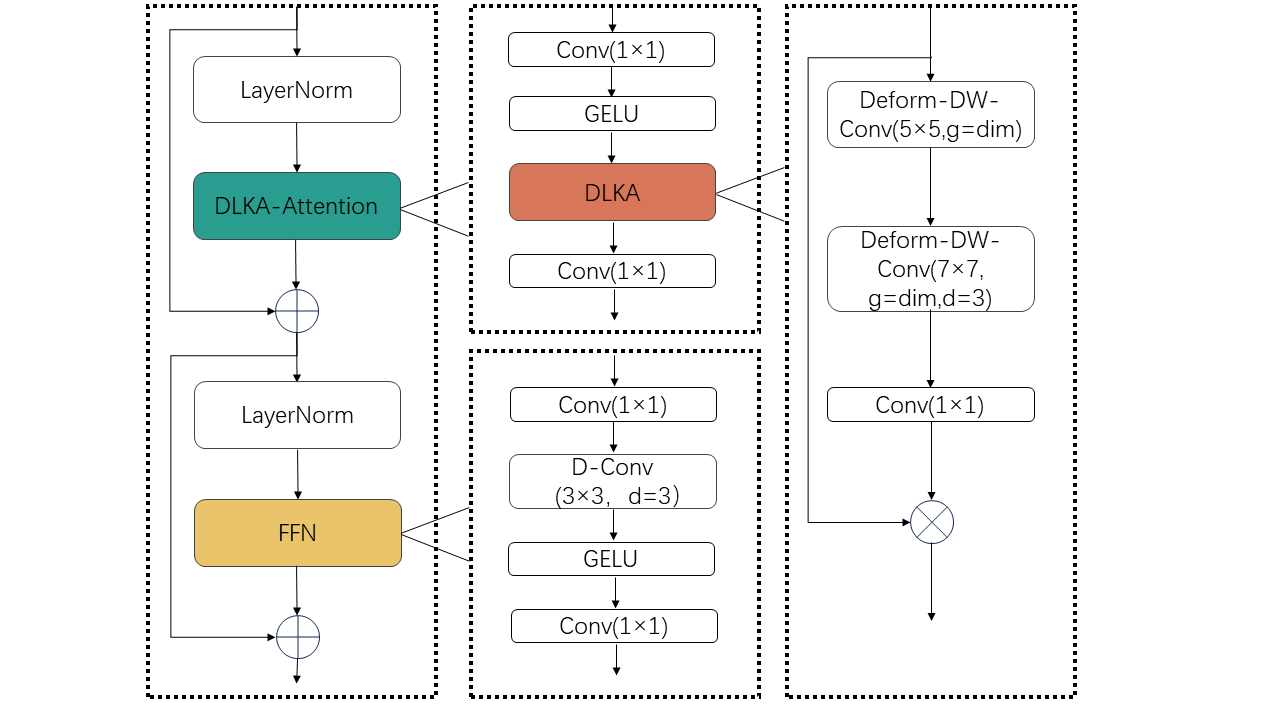

Supplement: S1 File — (ZIP) [file pone.0318578.s002.zip › suooprt information/images/Fig 3.tif]

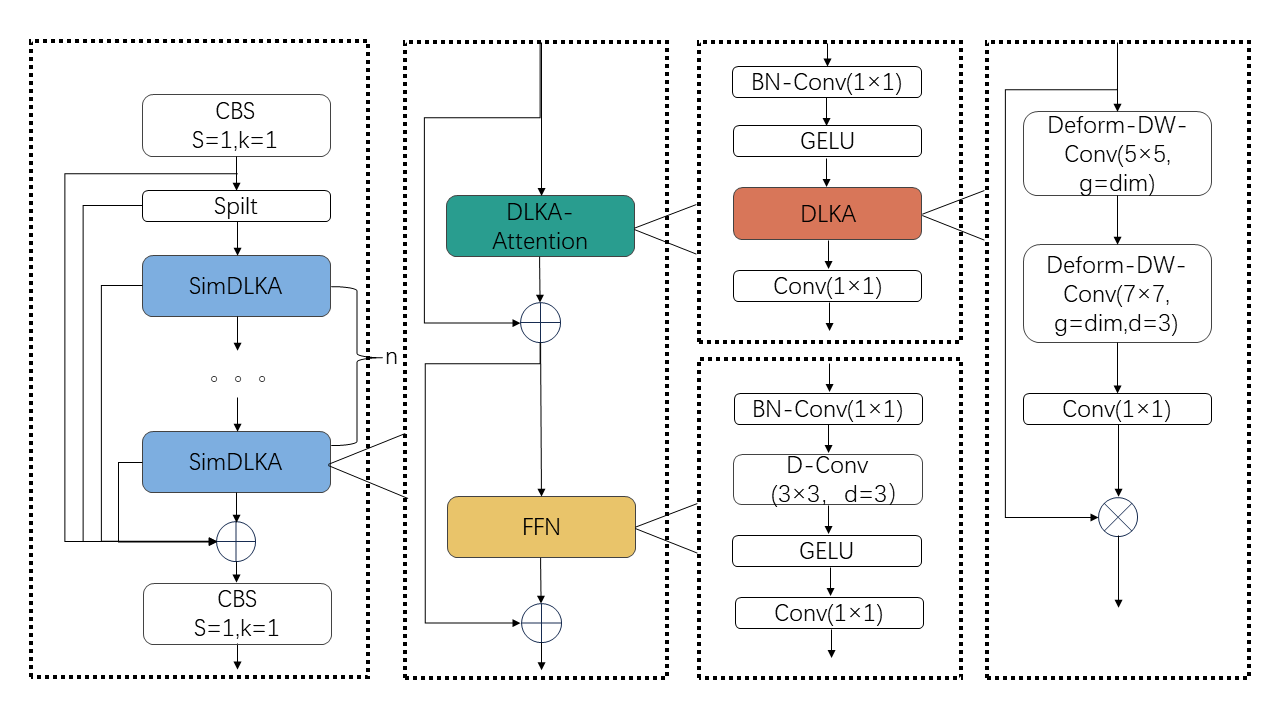

Supplement: S1 File — (ZIP) [file pone.0318578.s002.zip › suooprt information/images/Fig 4.tif]

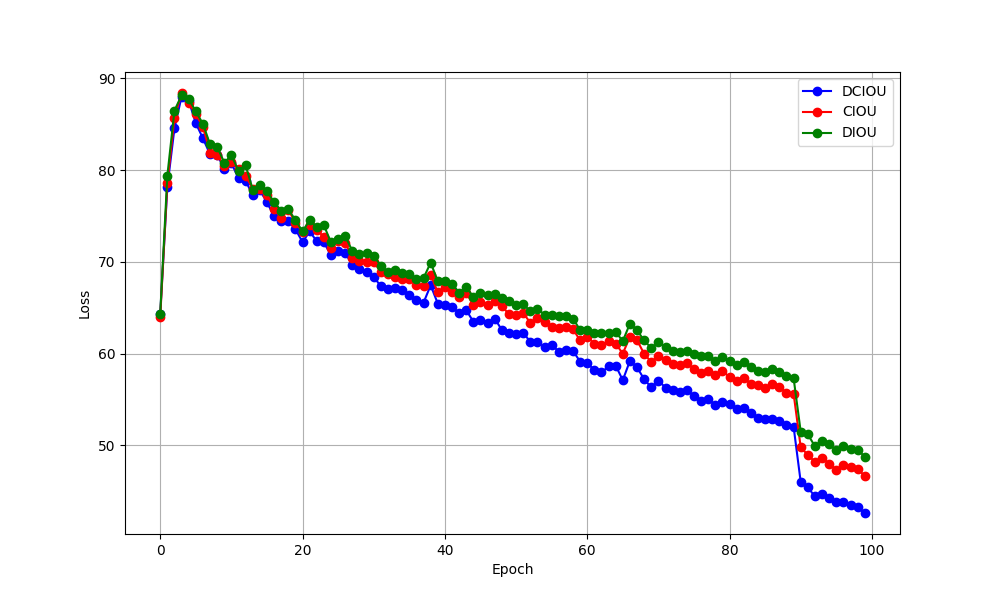

Supplement: S1 File — (ZIP) [file pone.0318578.s002.zip › suooprt information/images/Fig 5.tif]

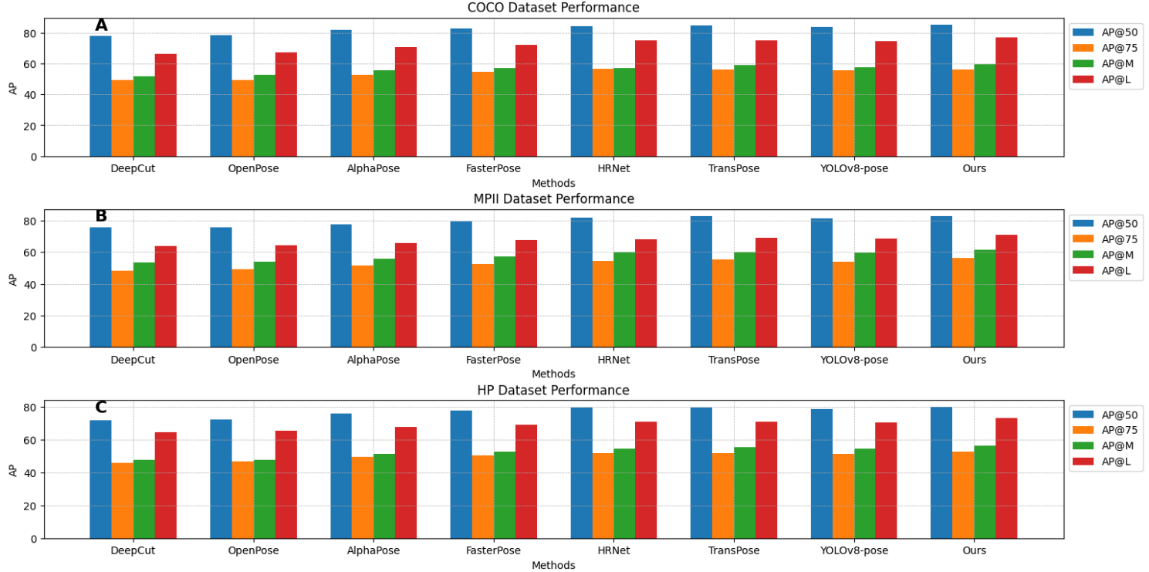

Supplement: S1 File — (ZIP) [file pone.0318578.s002.zip › suooprt information/images/Fig 6.tif]

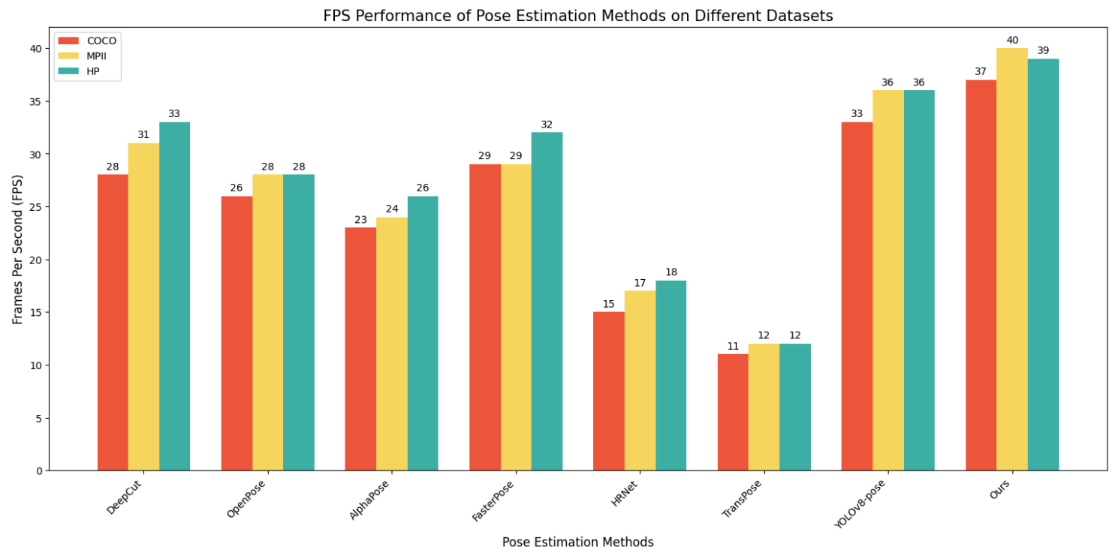

Supplement: S1 File — (ZIP) [file pone.0318578.s002.zip › suooprt information/images/Fig 7.tif]

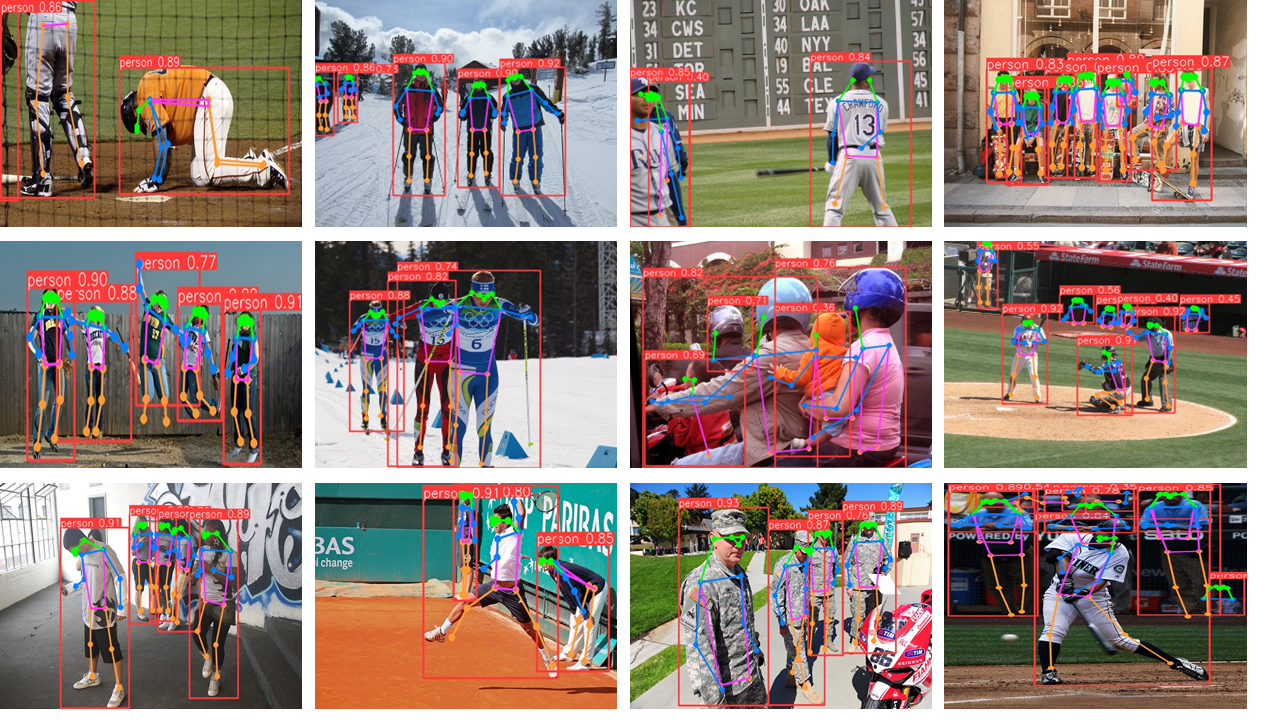

Supplement: S1 File — (ZIP) [file pone.0318578.s002.zip › suooprt information/images/Fig 8.tif]

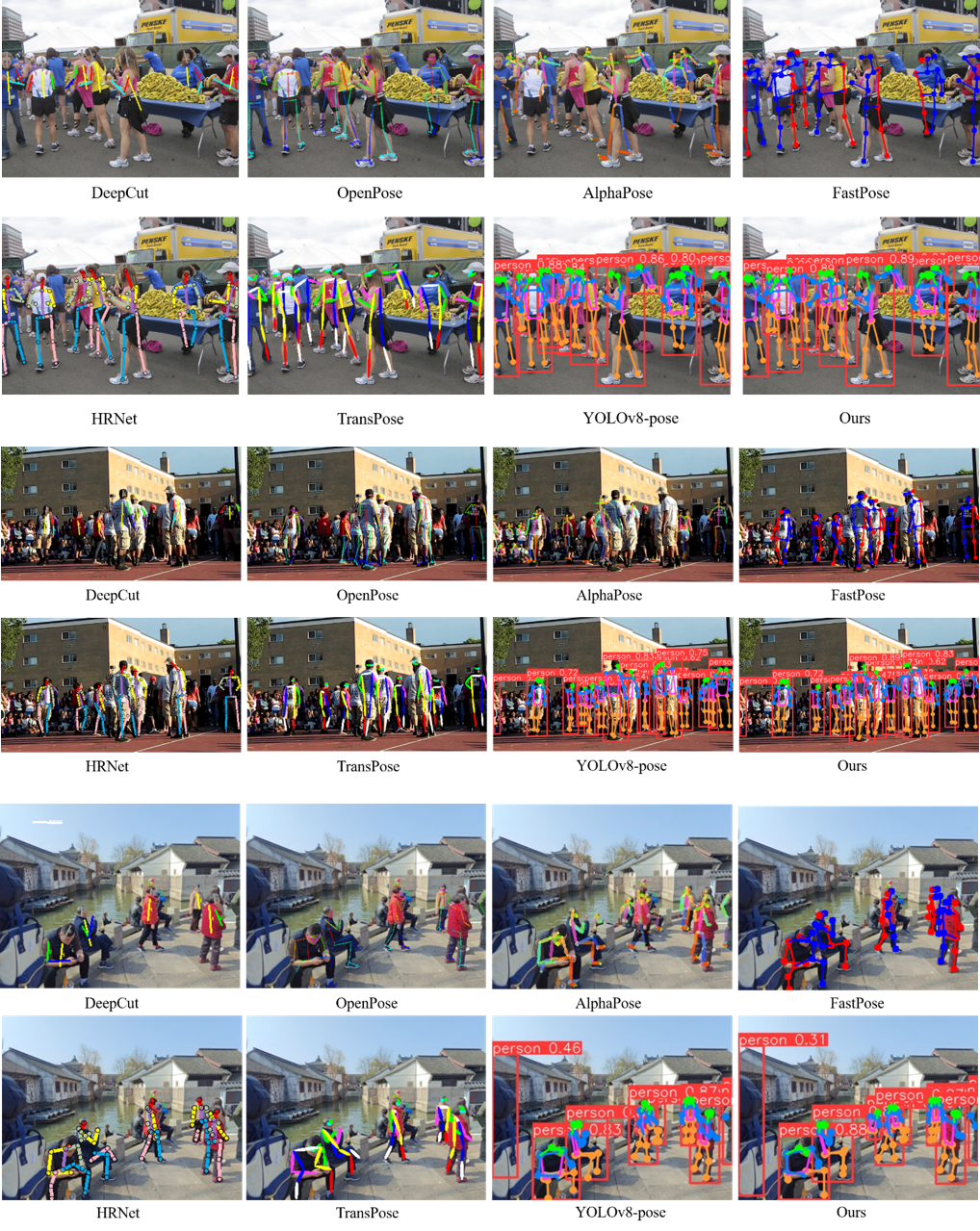

Supplement: S1 File — (ZIP) [file pone.0318578.s002.zip › suooprt information/images/Fig 9.tif]

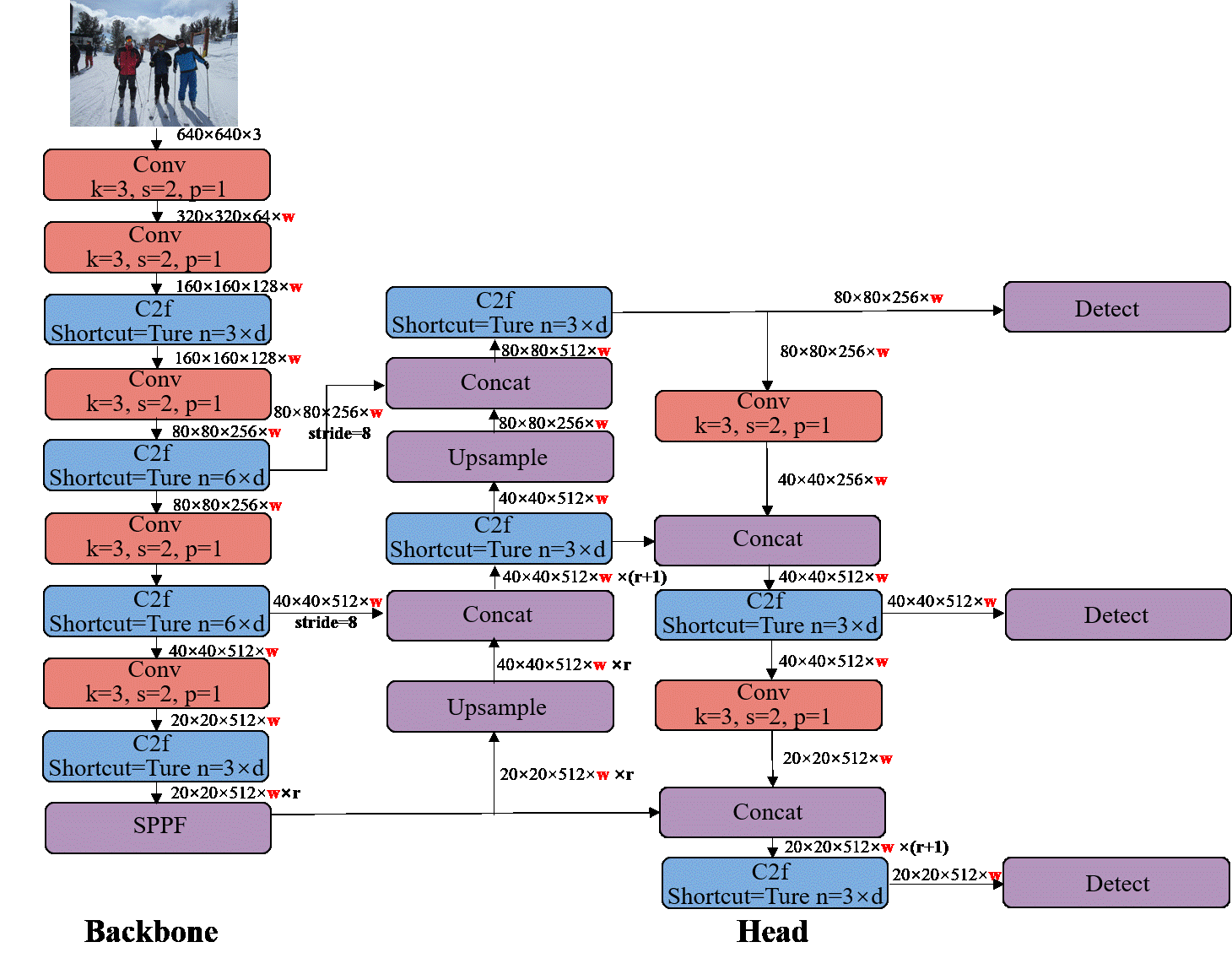

Supplement: S1 File — (ZIP) [file pone.0318578.s002.zip › suooprt information/images/Fig1.tif]

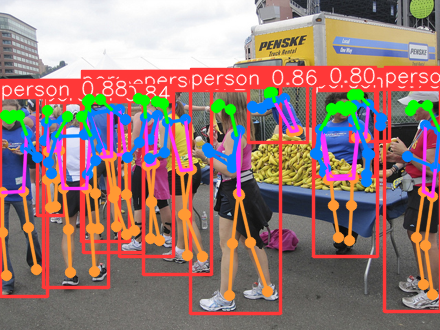

Supplement: S1 File — (ZIP) [file pone.0318578.s002.zip › suooprt information/pose/predict/image1.png]

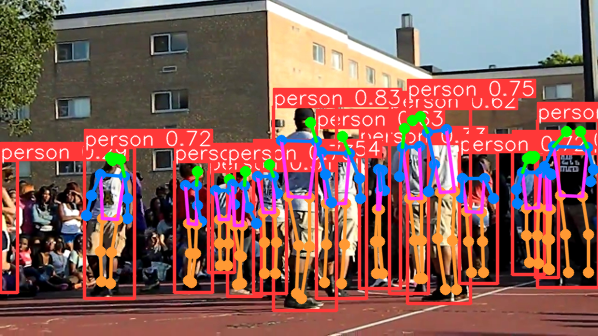

Supplement: S1 File — (ZIP) [file pone.0318578.s002.zip › suooprt information/pose/predict2/images2.png]

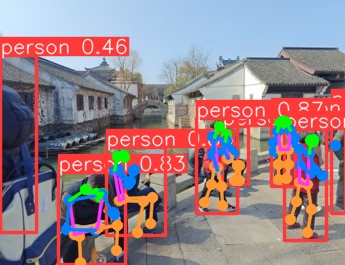

Supplement: S1 File — (ZIP) [file pone.0318578.s002.zip › suooprt information/pose/predict3/images3.jpg]

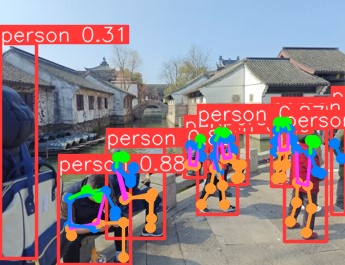

Supplement: S1 File — (ZIP) [file pone.0318578.s002.zip › suooprt information/pose/predict4/images3.jpg]

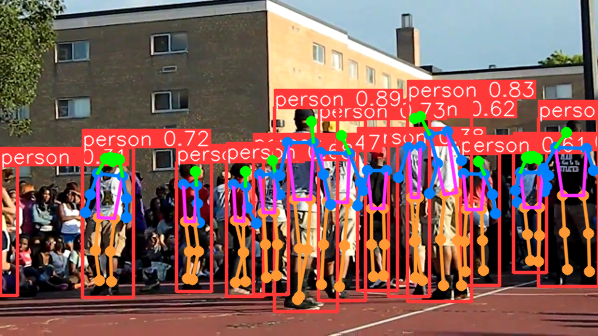

Supplement: S1 File — (ZIP) [file pone.0318578.s002.zip › suooprt information/pose/predict5/images2.png]

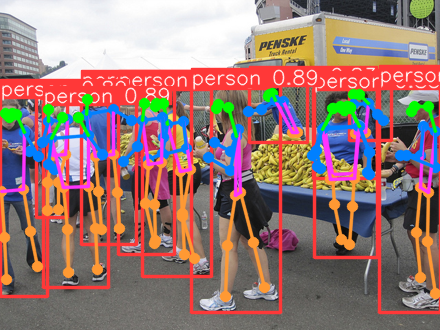

Supplement: S1 File — (ZIP) [file pone.0318578.s002.zip › suooprt information/pose/predict6/image1.png]

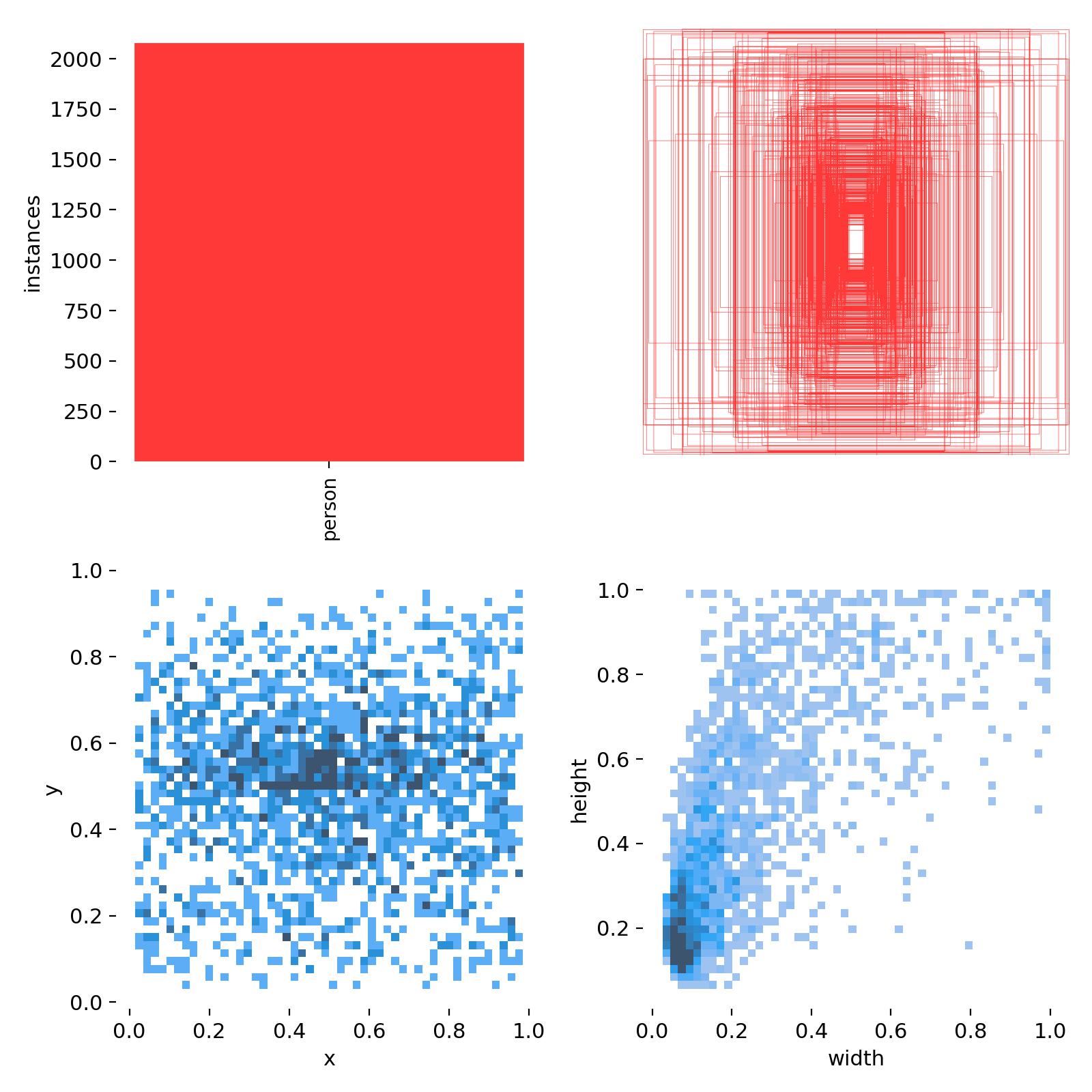

Supplement: S1 File — (ZIP) [file pone.0318578.s002.zip › suooprt information/pose/train28/labels.jpg]

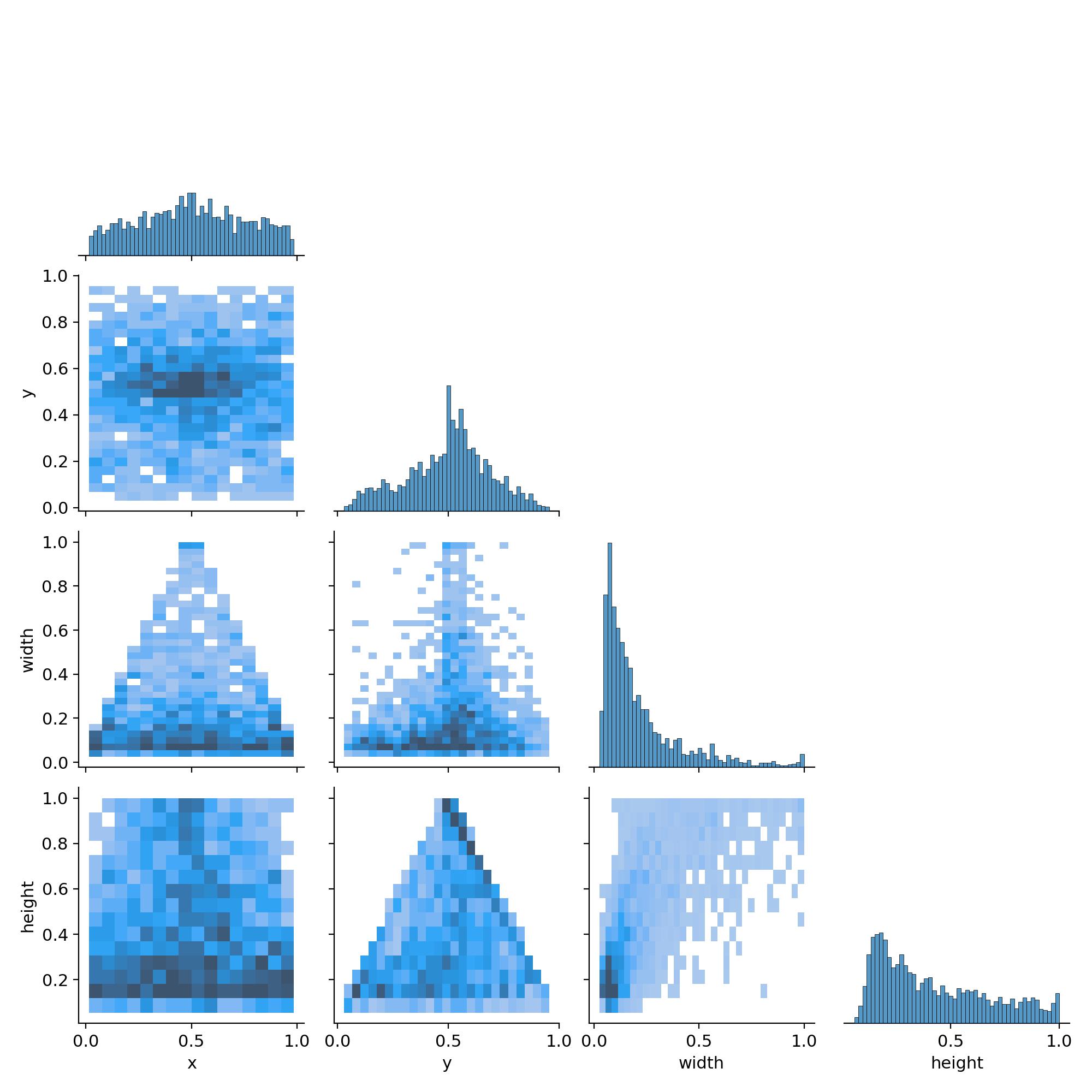

Supplement: S1 File — (ZIP) [file pone.0318578.s002.zip › suooprt information/pose/train28/labels_correlogram.jpg]

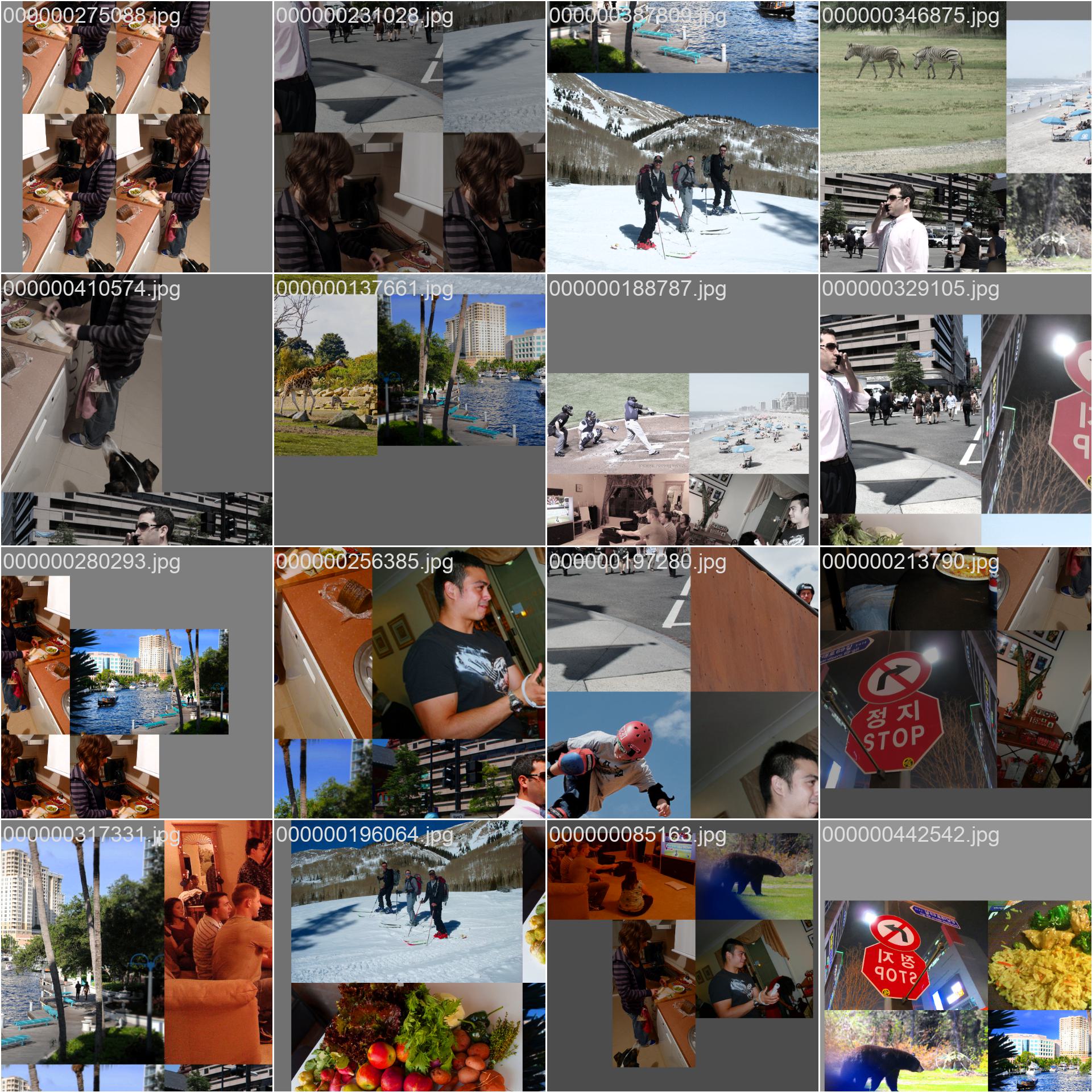

Supplement: S1 File — (ZIP) [file pone.0318578.s002.zip › suooprt information/pose/train28/train_batch0.jpg]

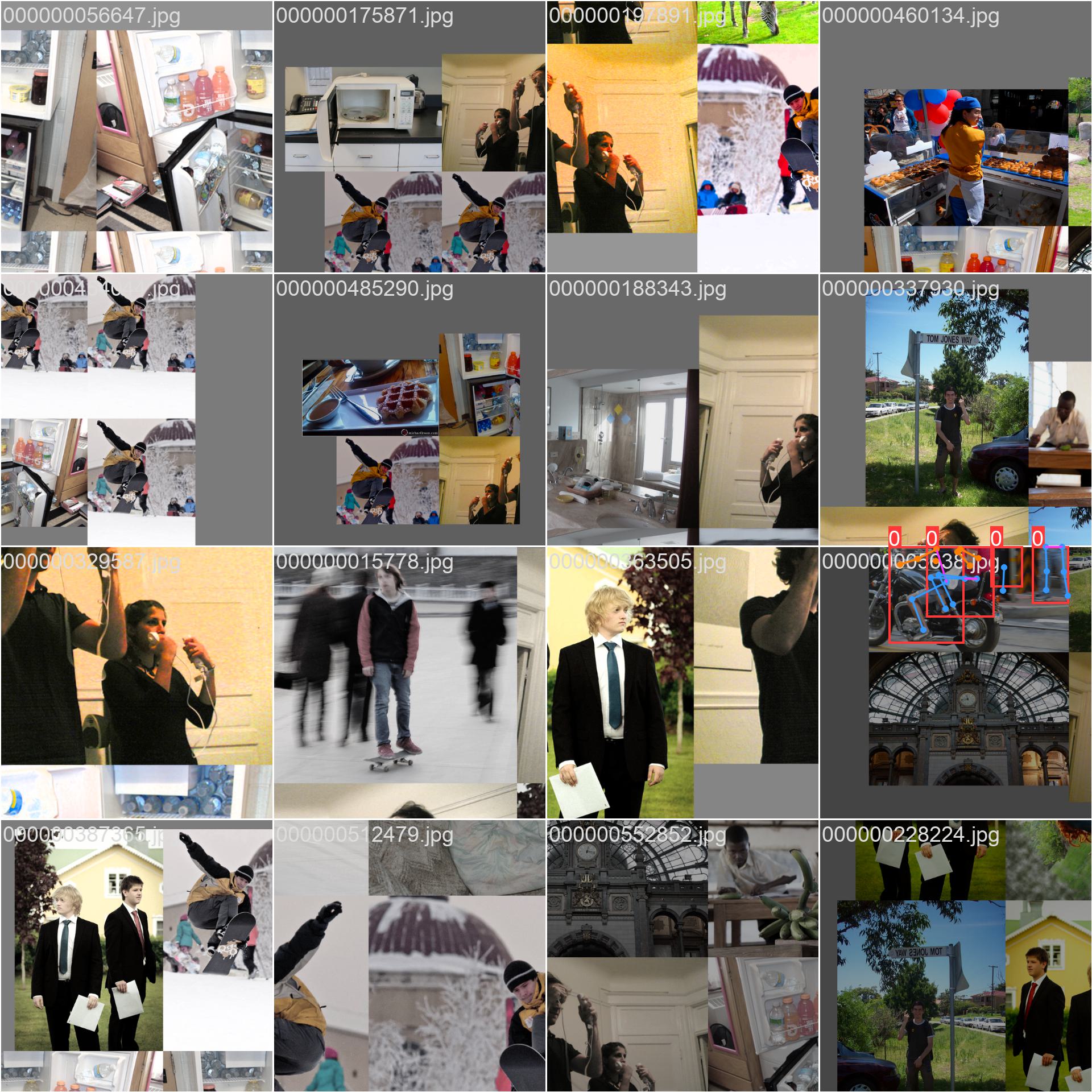

Supplement: S1 File — (ZIP) [file pone.0318578.s002.zip › suooprt information/pose/train28/train_batch1.jpg]

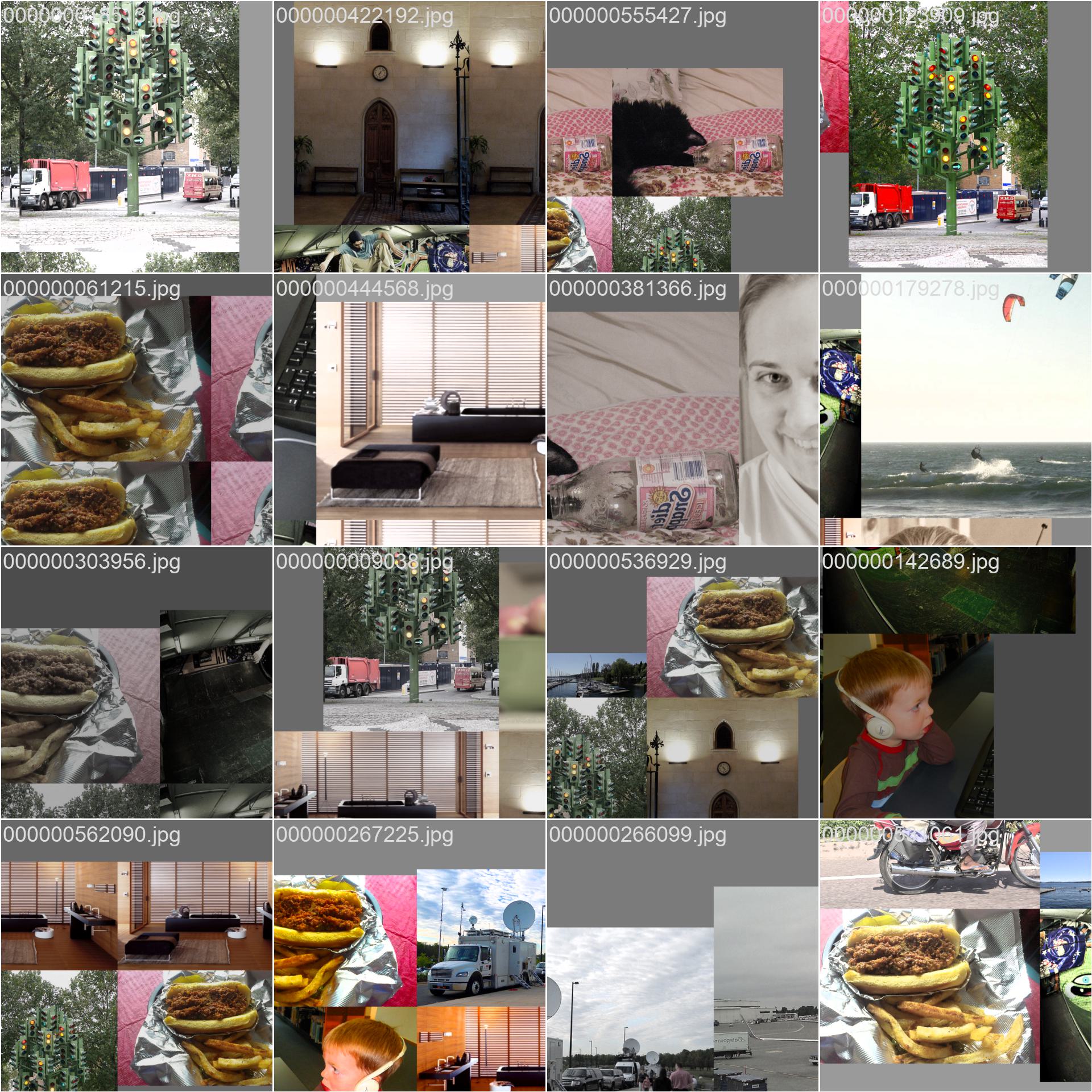

Supplement: S1 File — (ZIP) [file pone.0318578.s002.zip › suooprt information/pose/train28/train_batch2.jpg]

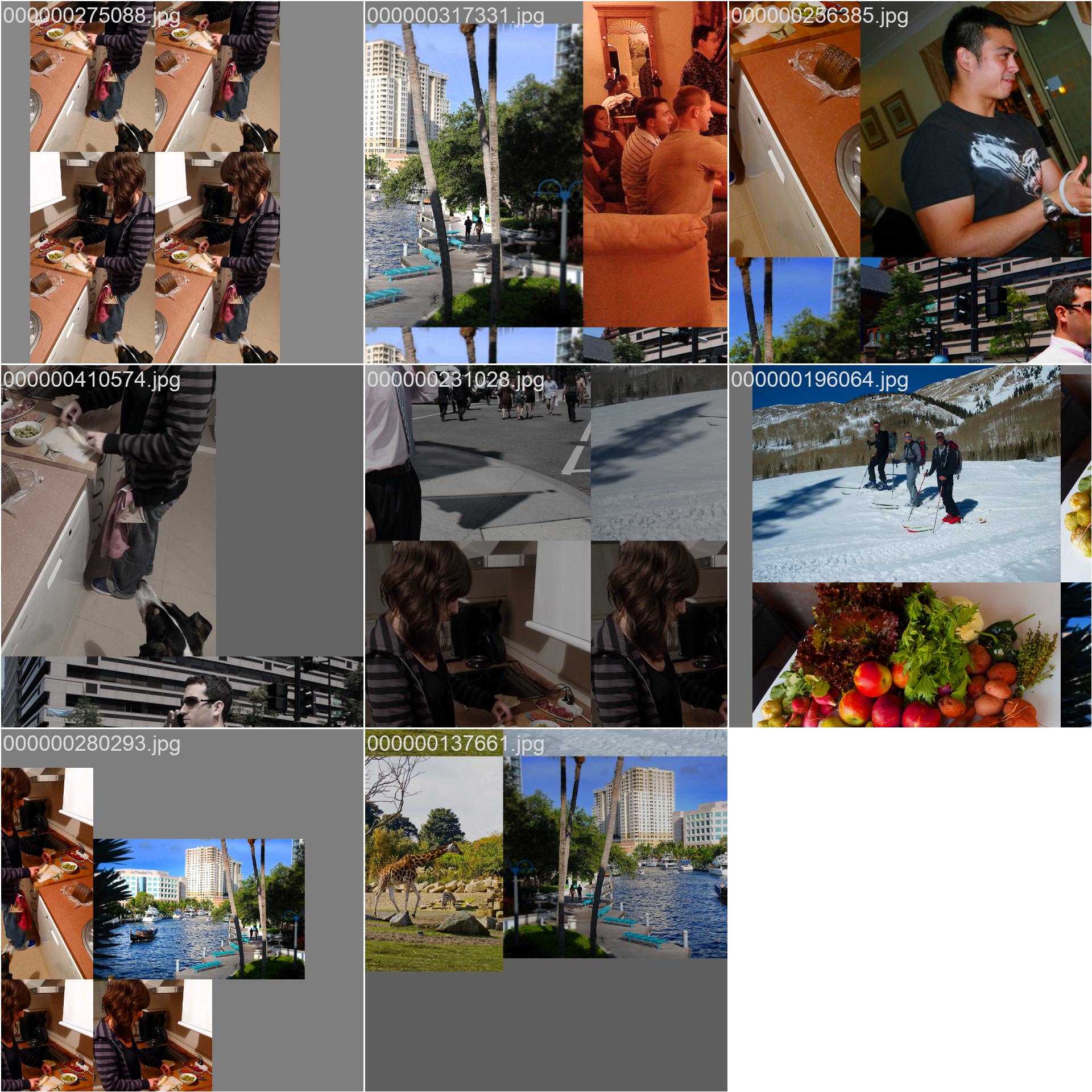

Supplement: S1 File — (ZIP) [file pone.0318578.s002.zip › suooprt information/pose/train29/train_batch0.jpg]

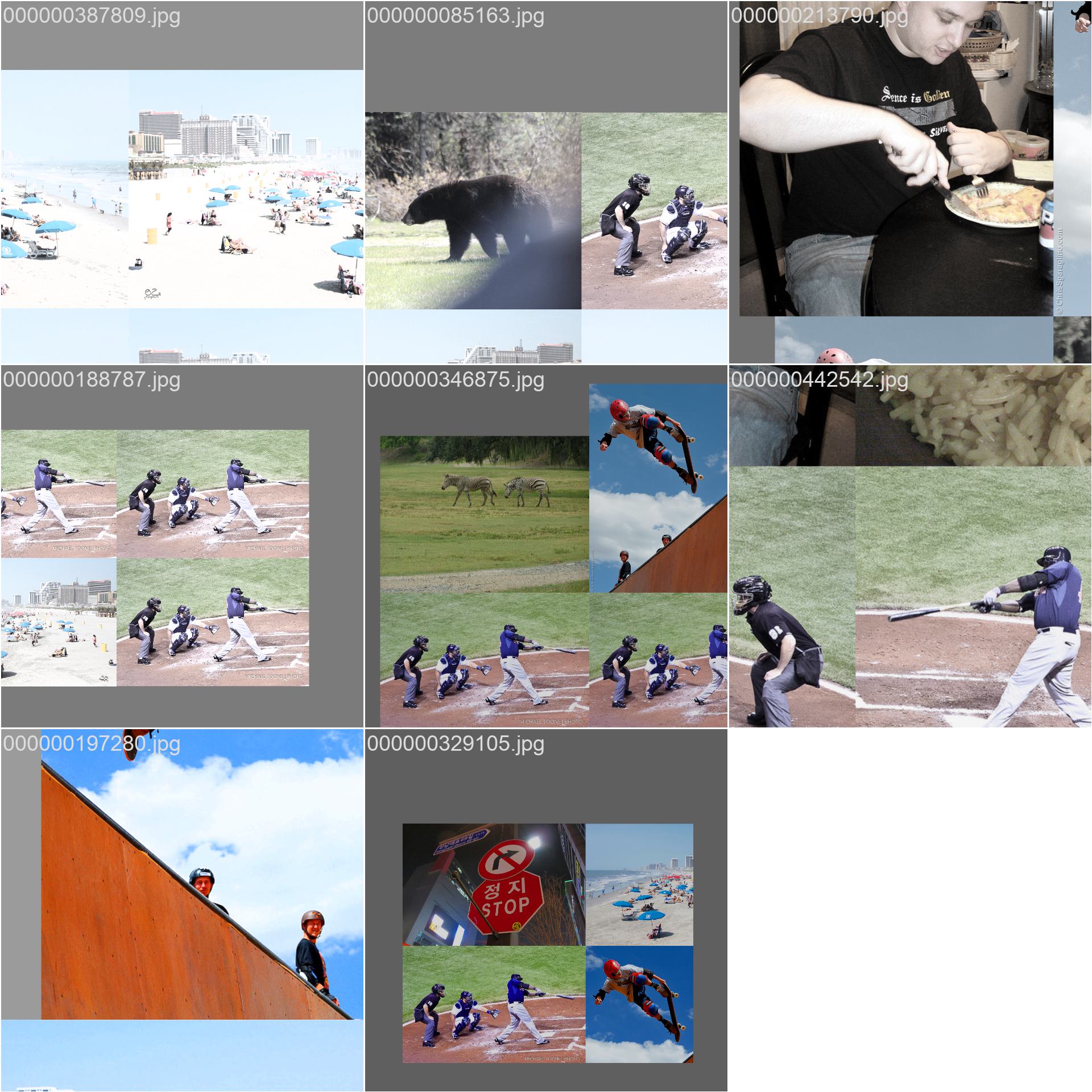

Supplement: S1 File — (ZIP) [file pone.0318578.s002.zip › suooprt information/pose/train29/train_batch1.jpg]

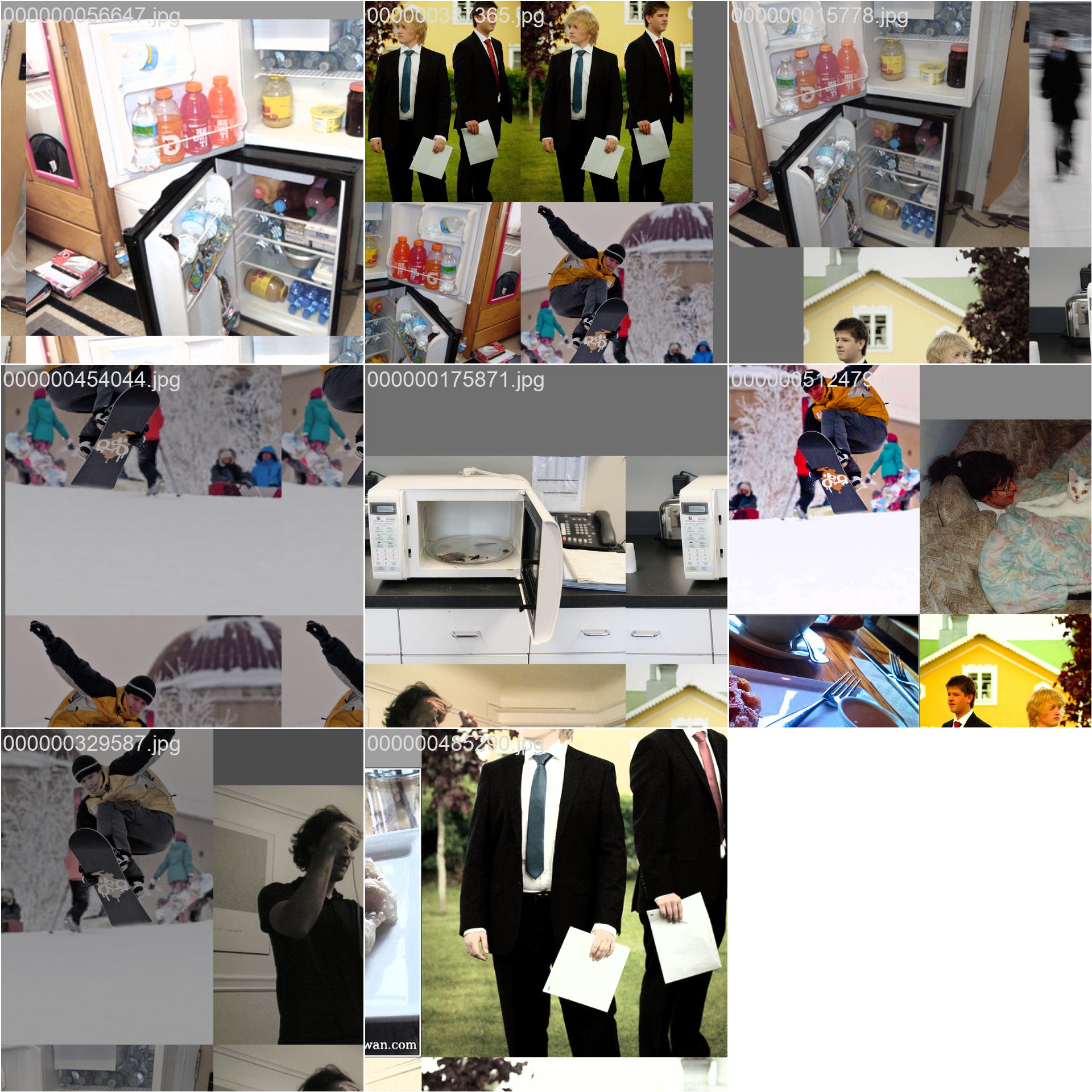

Supplement: S1 File — (ZIP) [file pone.0318578.s002.zip › suooprt information/pose/train29/train_batch2.jpg]

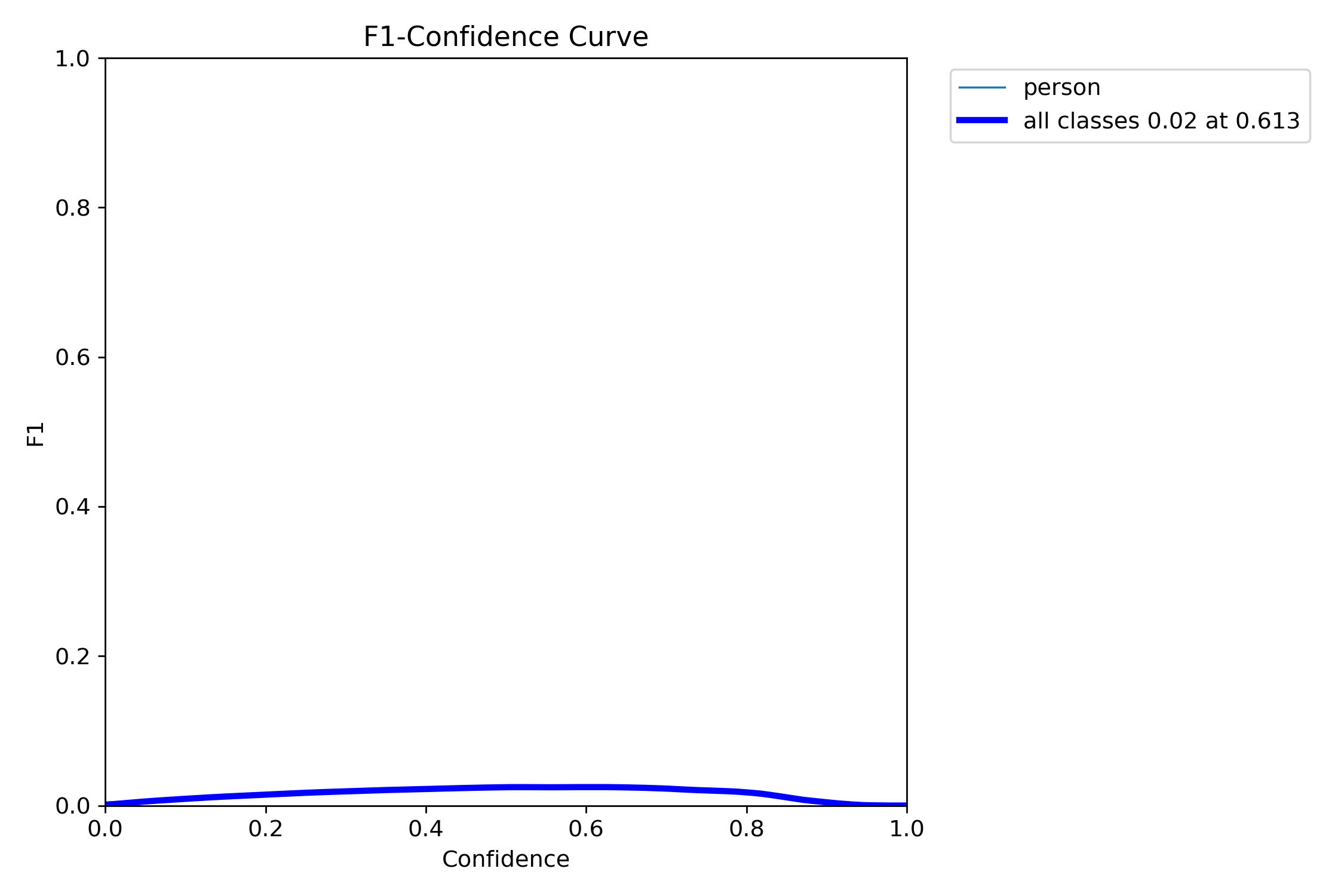

Supplement: S1 File — (ZIP) [file pone.0318578.s002.zip › suooprt information/pose/train30/BoxF1_curve.png]

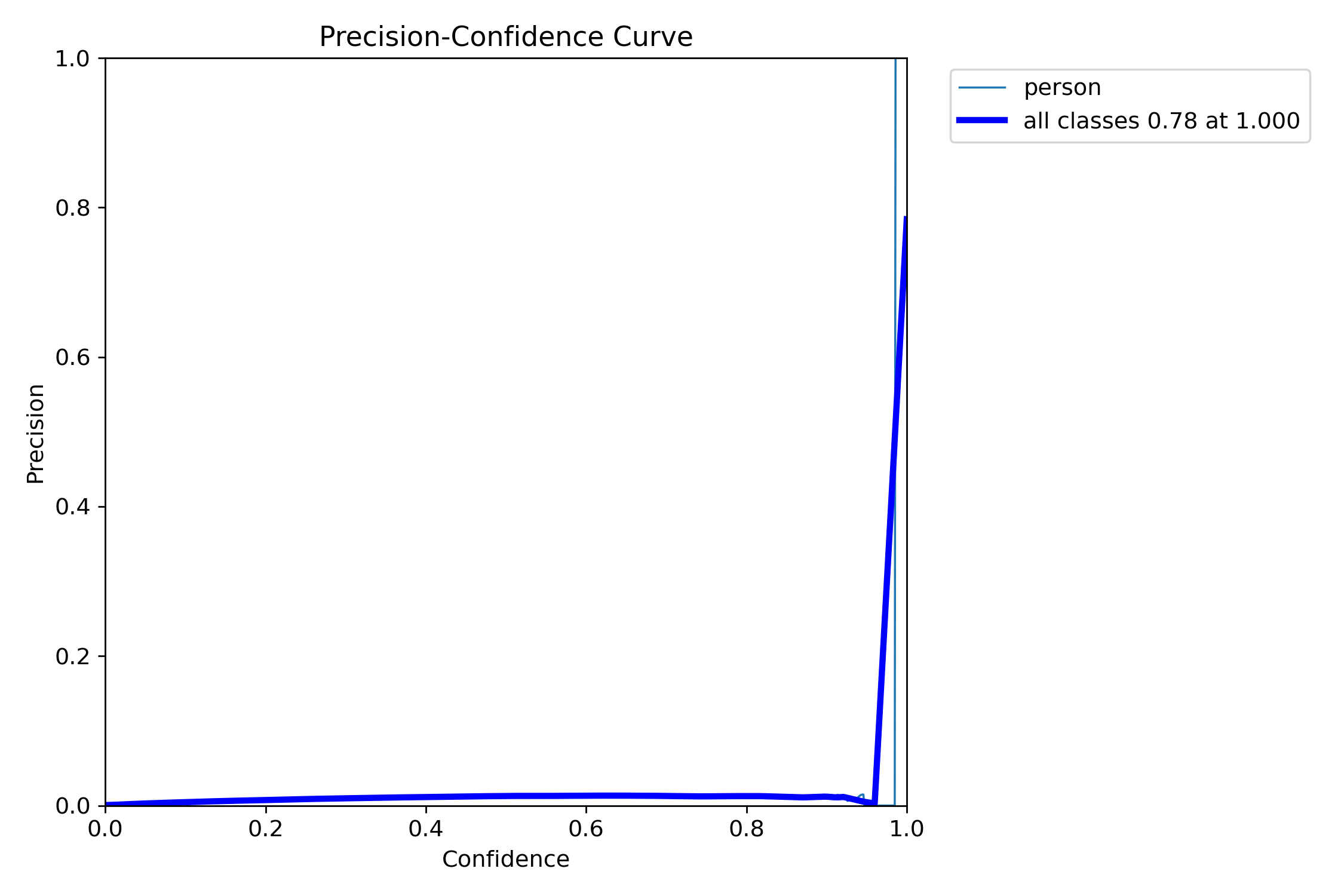

Supplement: S1 File — (ZIP) [file pone.0318578.s002.zip › suooprt information/pose/train30/BoxP_curve.png]

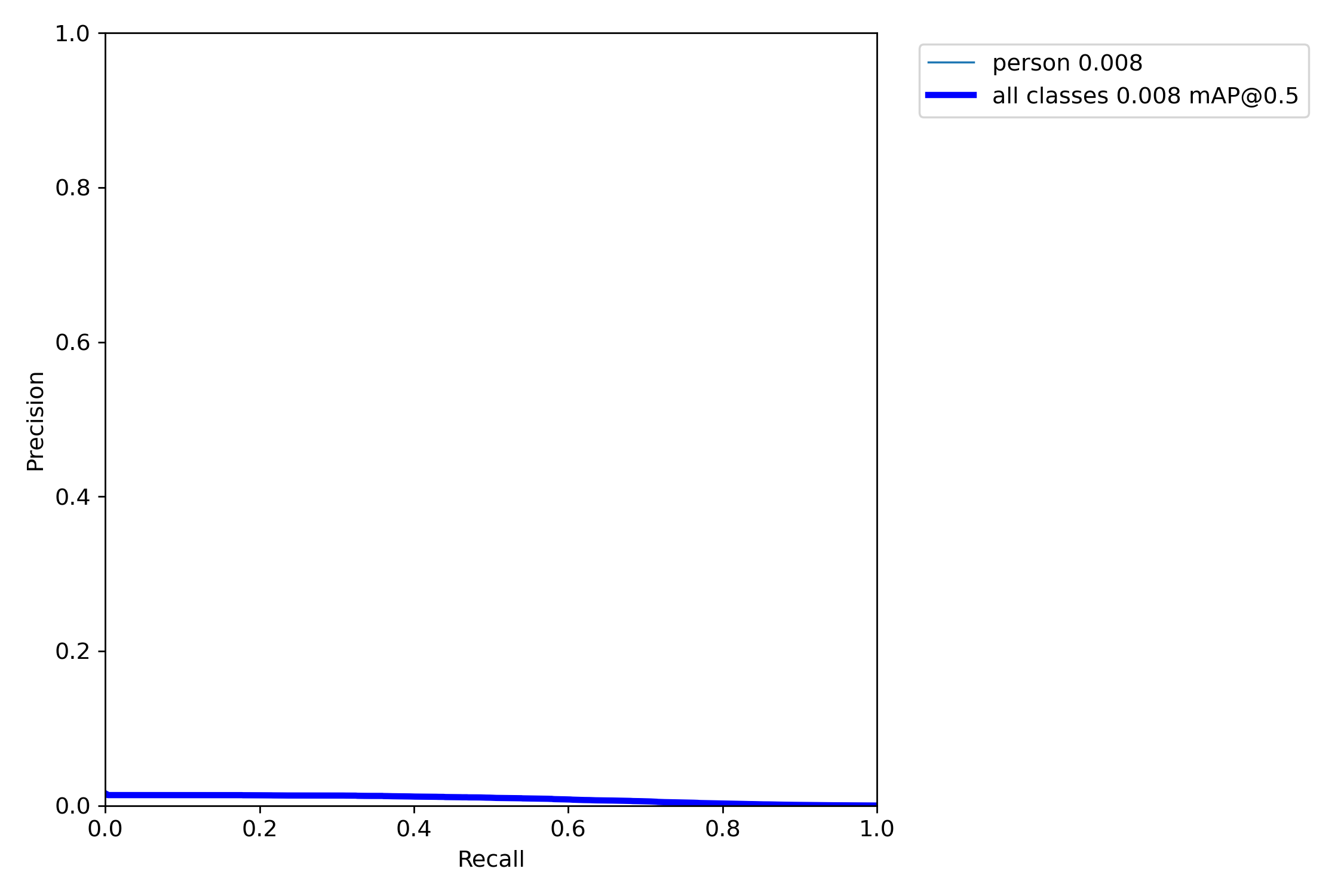

Supplement: S1 File — (ZIP) [file pone.0318578.s002.zip › suooprt information/pose/train30/BoxPR_curve.png]

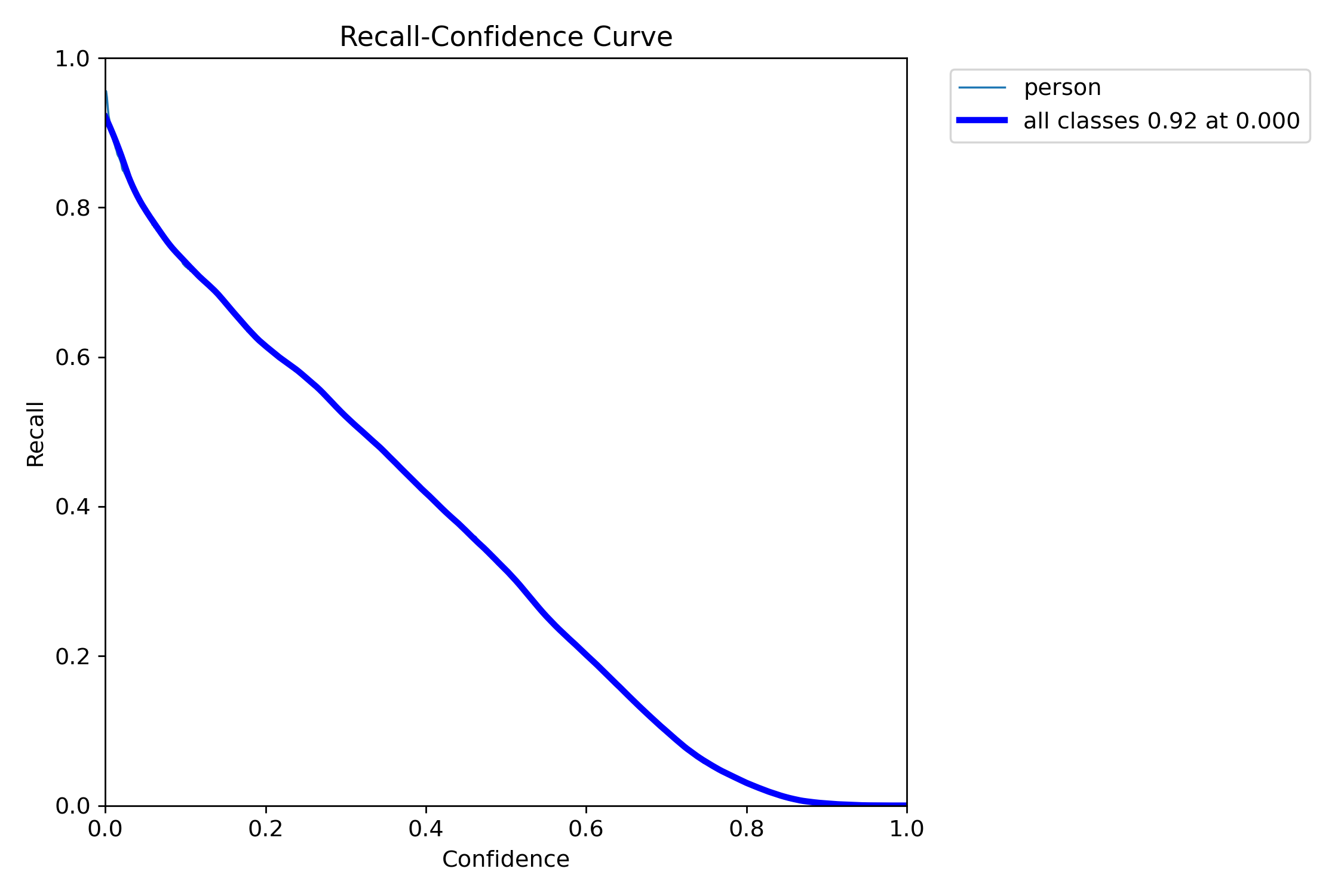

Supplement: S1 File — (ZIP) [file pone.0318578.s002.zip › suooprt information/pose/train30/BoxR_curve.png]

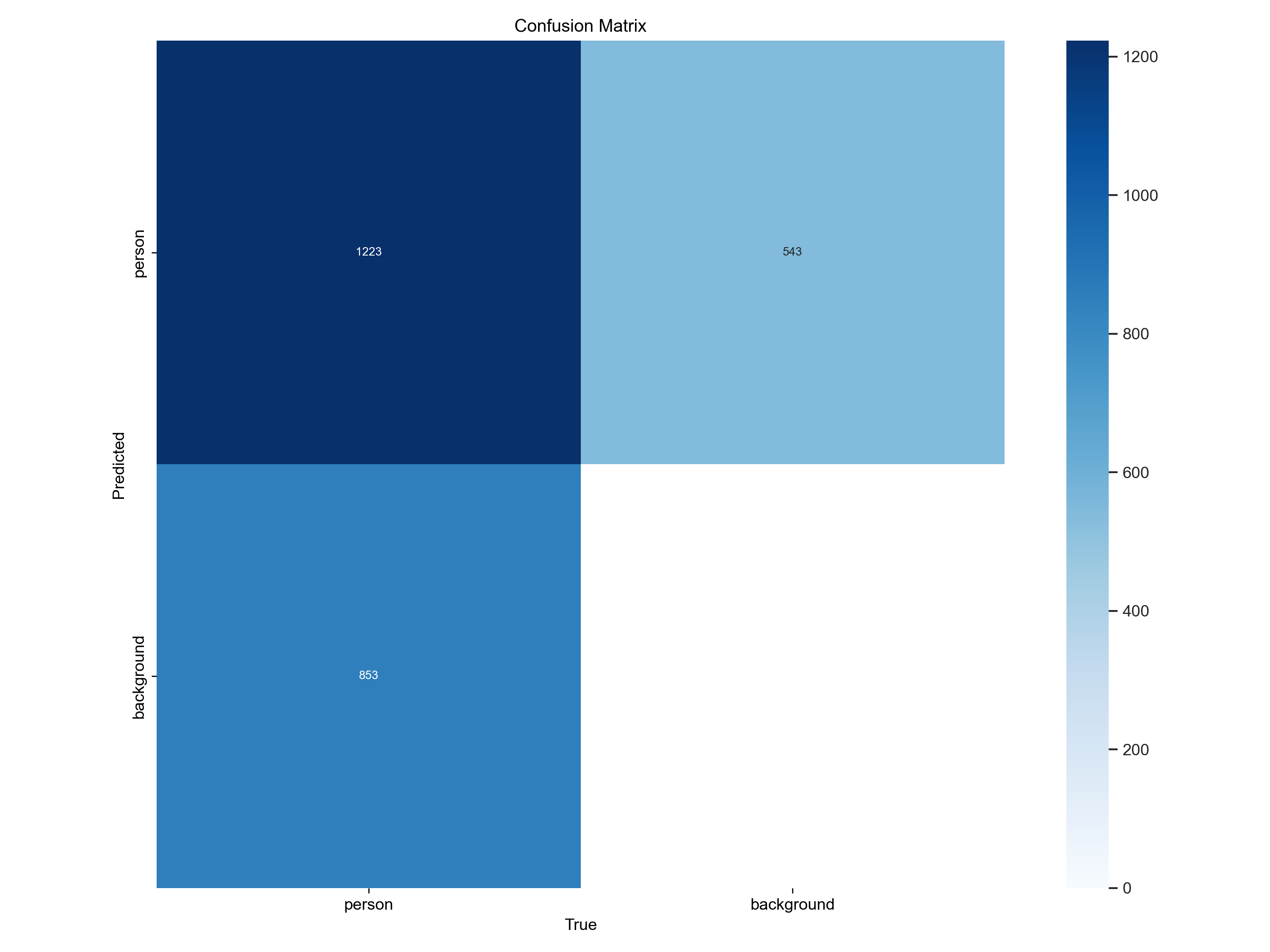

Supplement: S1 File — (ZIP) [file pone.0318578.s002.zip › suooprt information/pose/train30/confusion_matrix.png]

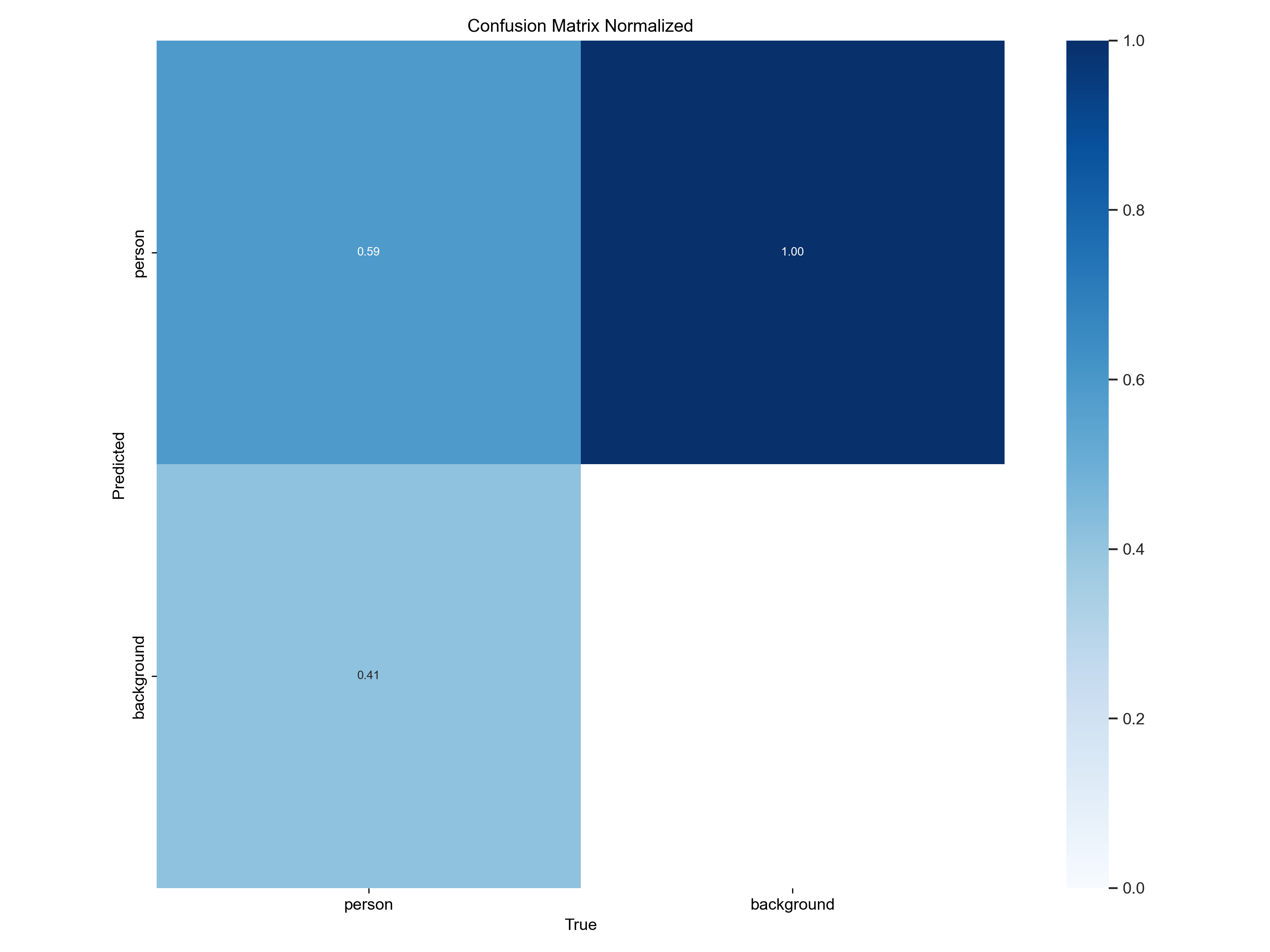

Supplement: S1 File — (ZIP) [file pone.0318578.s002.zip › suooprt information/pose/train30/confusion_matrix_normalized.png]

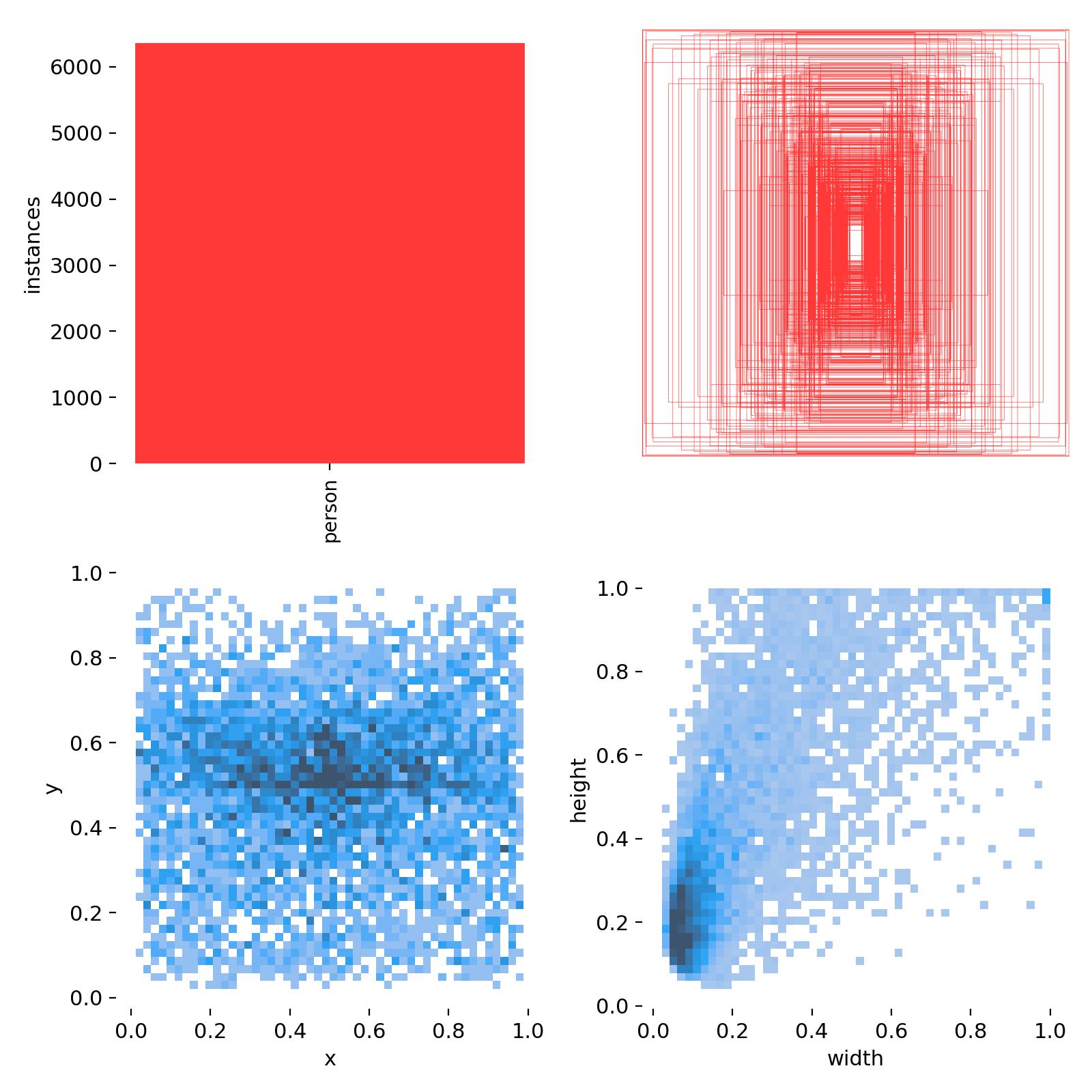

Supplement: S1 File — (ZIP) [file pone.0318578.s002.zip › suooprt information/pose/train30/labels.jpg]

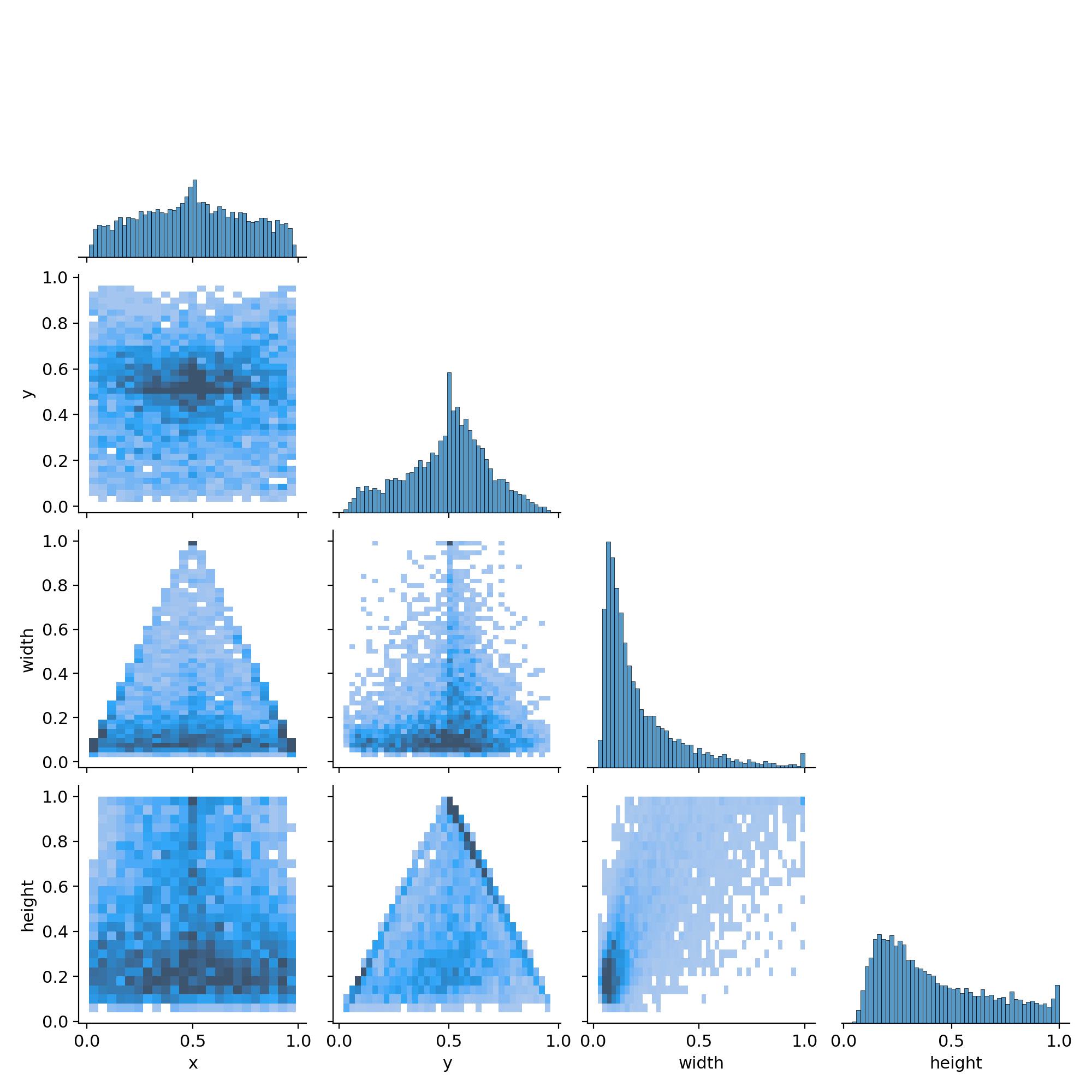

Supplement: S1 File — (ZIP) [file pone.0318578.s002.zip › suooprt information/pose/train30/labels_correlogram.jpg]

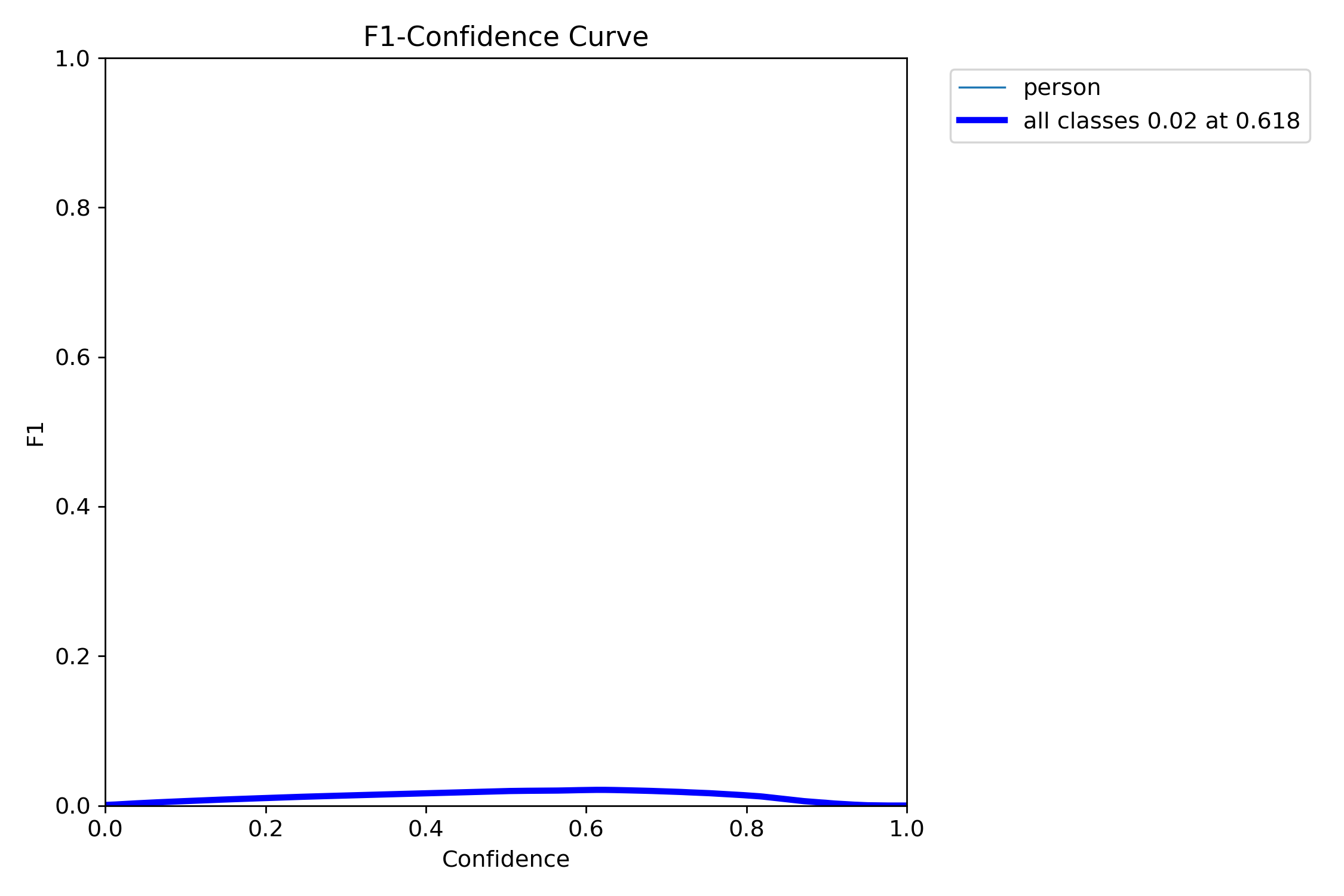

Supplement: S1 File — (ZIP) [file pone.0318578.s002.zip › suooprt information/pose/train30/PoseF1_curve.png]

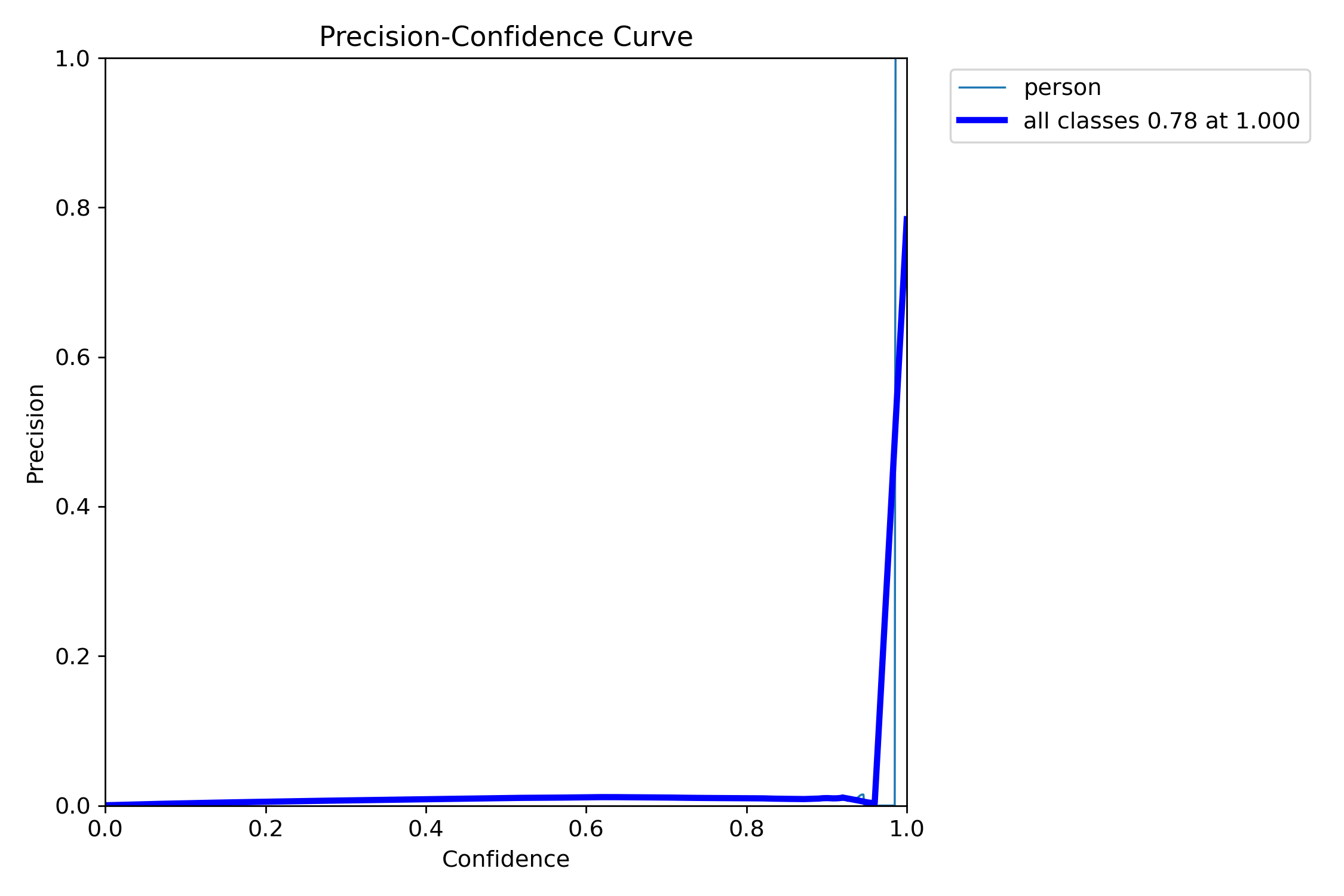

Supplement: S1 File — (ZIP) [file pone.0318578.s002.zip › suooprt information/pose/train30/PoseP_curve.png]

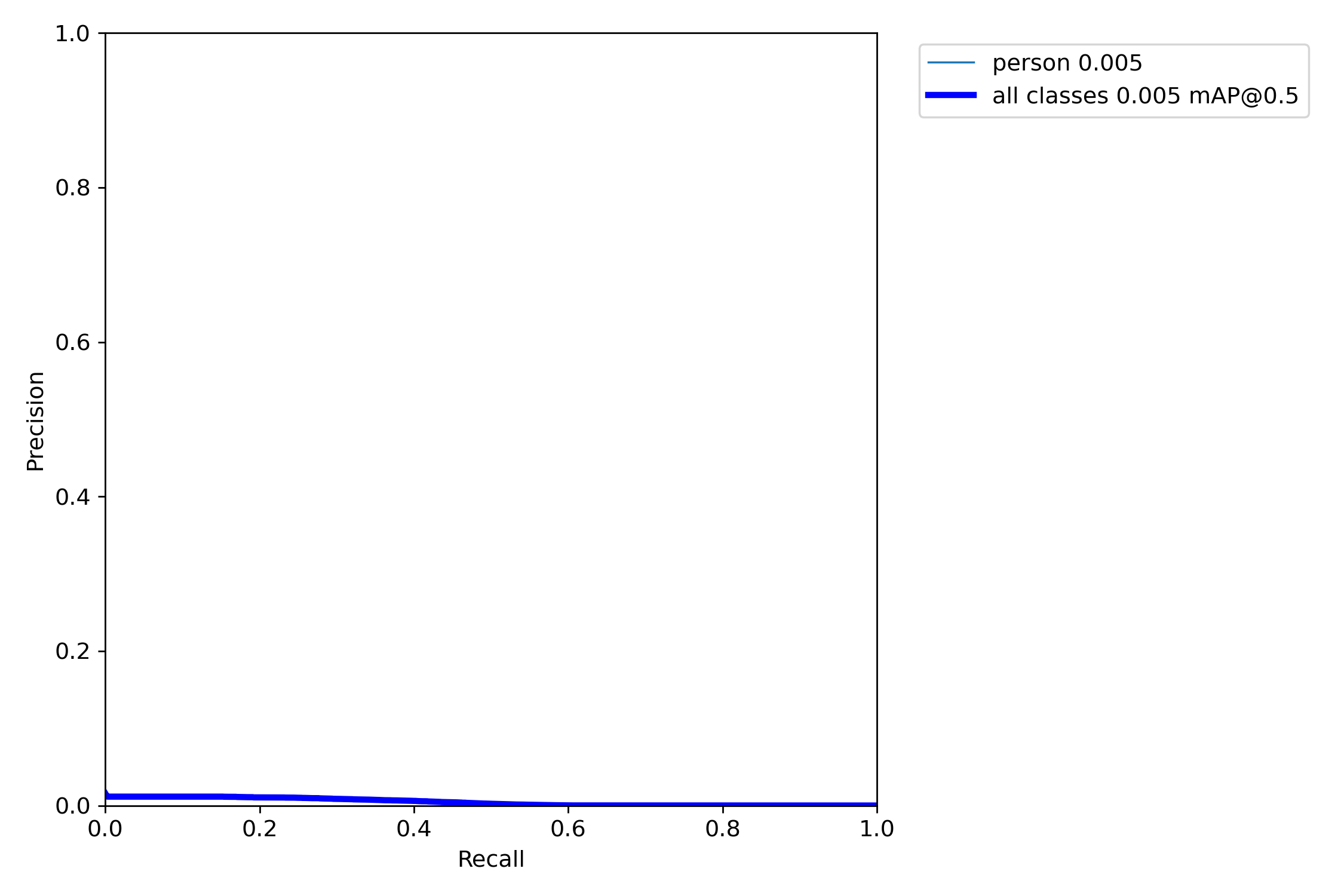

Supplement: S1 File — (ZIP) [file pone.0318578.s002.zip › suooprt information/pose/train30/PosePR_curve.png]

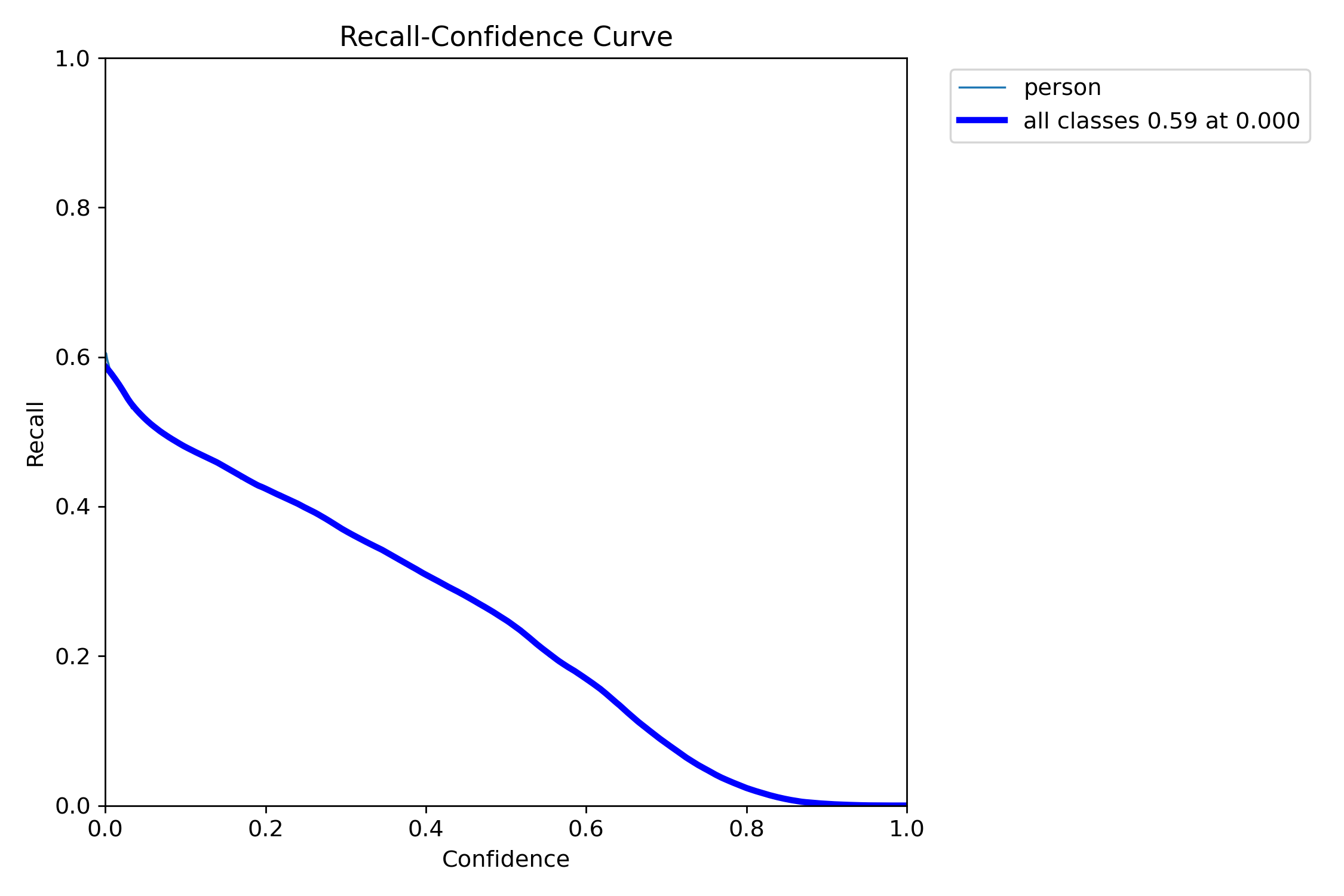

Supplement: S1 File — (ZIP) [file pone.0318578.s002.zip › suooprt information/pose/train30/PoseR_curve.png]

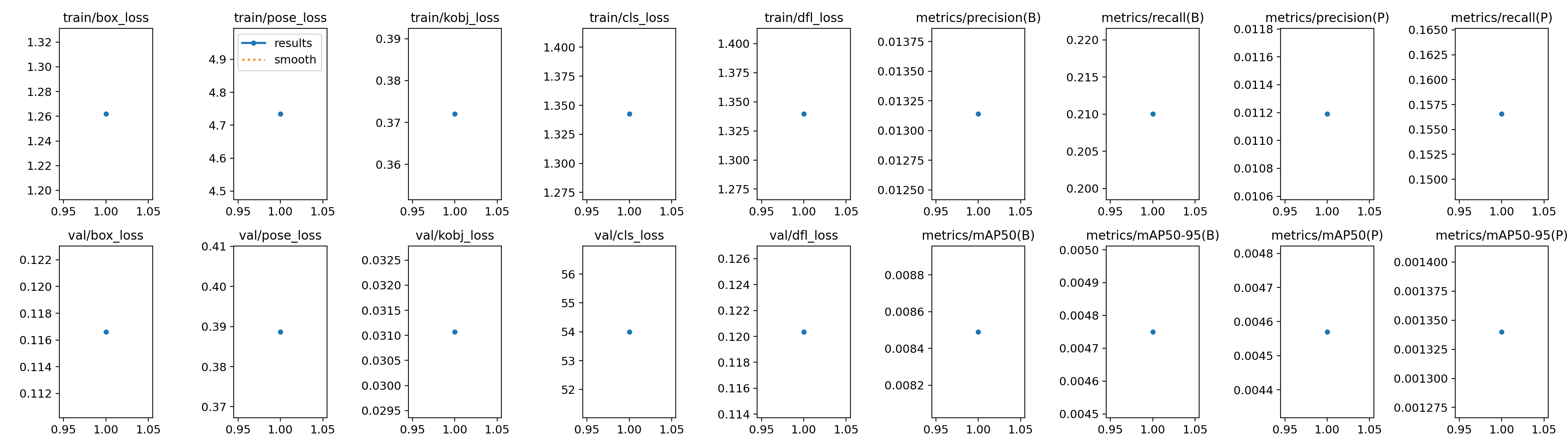

Supplement: S1 File — (ZIP) [file pone.0318578.s002.zip › suooprt information/pose/train30/results.png]

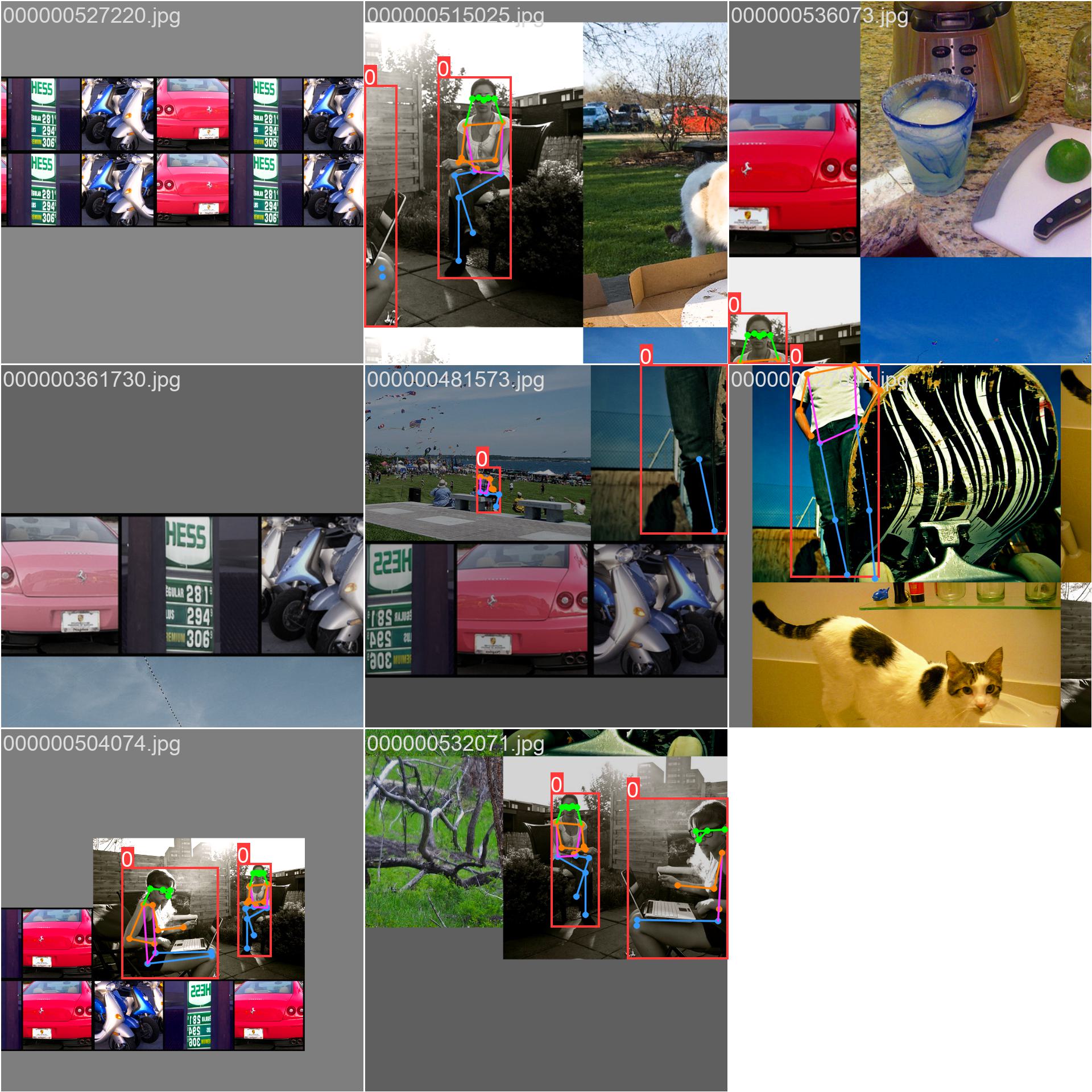

Supplement: S1 File — (ZIP) [file pone.0318578.s002.zip › suooprt information/pose/train30/train_batch0.jpg]

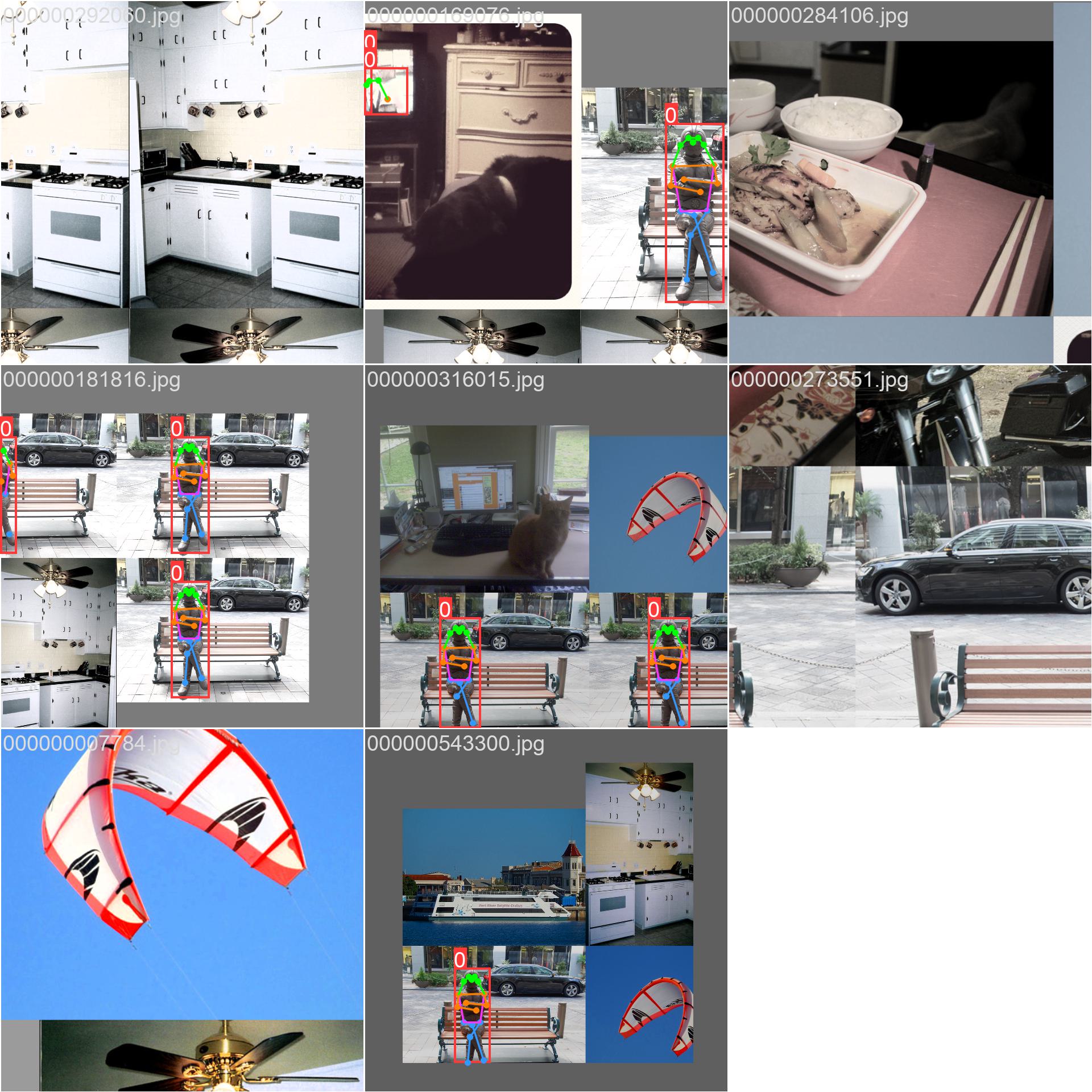

Supplement: S1 File — (ZIP) [file pone.0318578.s002.zip › suooprt information/pose/train30/train_batch1.jpg]

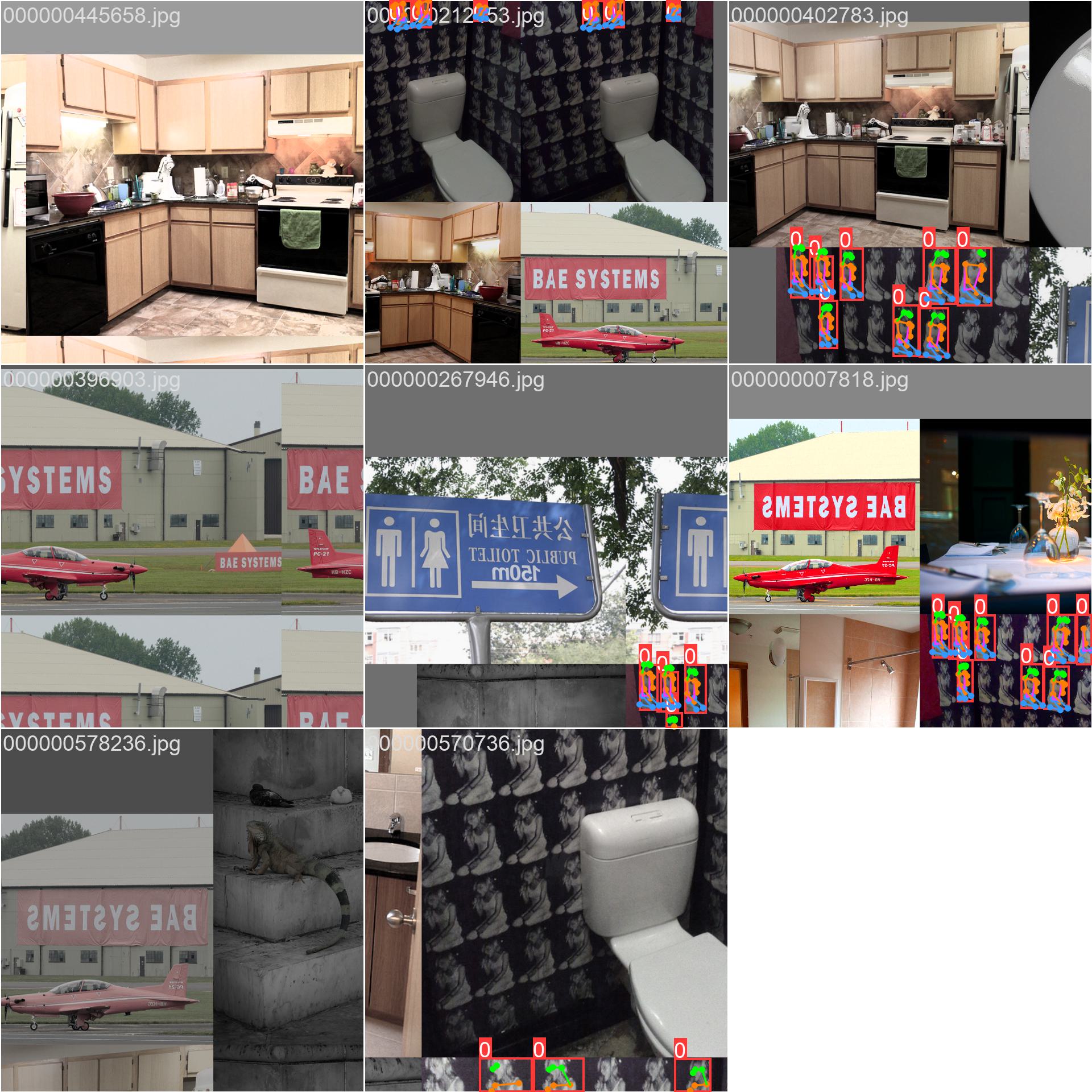

Supplement: S1 File — (ZIP) [file pone.0318578.s002.zip › suooprt information/pose/train30/train_batch2.jpg]

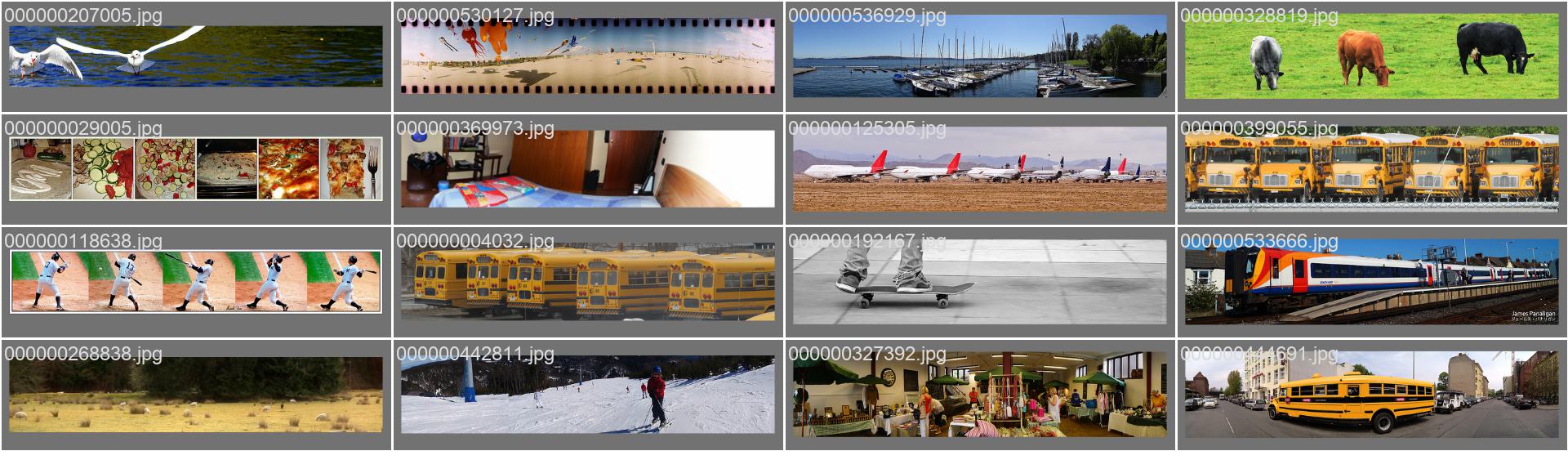

Supplement: S1 File — (ZIP) [file pone.0318578.s002.zip › suooprt information/pose/train30/val_batch0_labels.jpg]

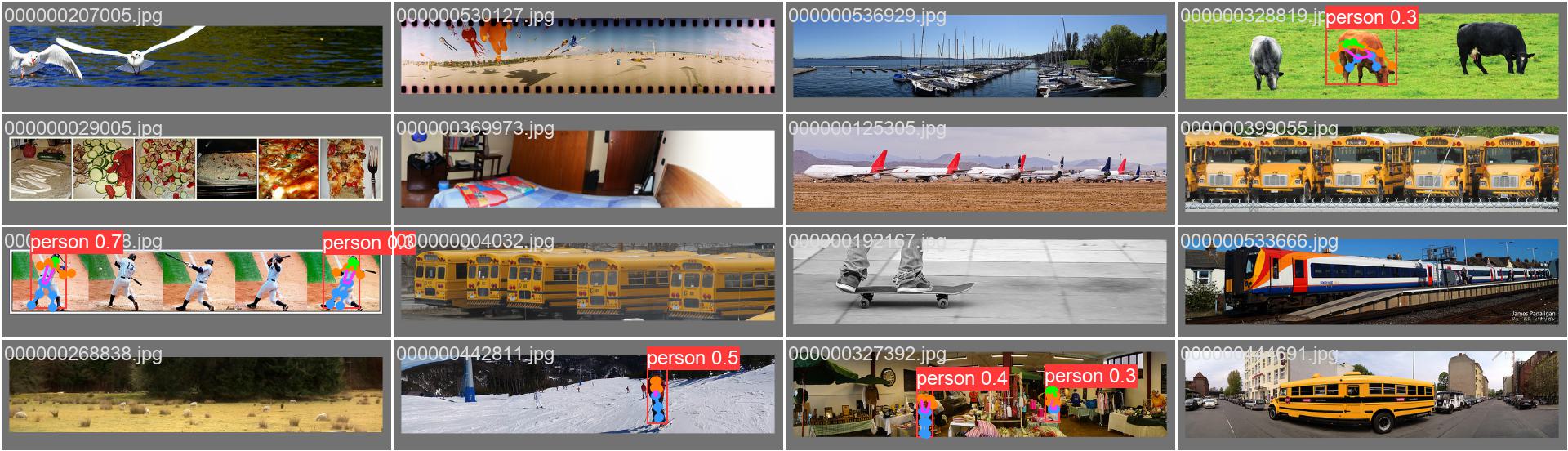

Supplement: S1 File — (ZIP) [file pone.0318578.s002.zip › suooprt information/pose/train30/val_batch0_pred.jpg]

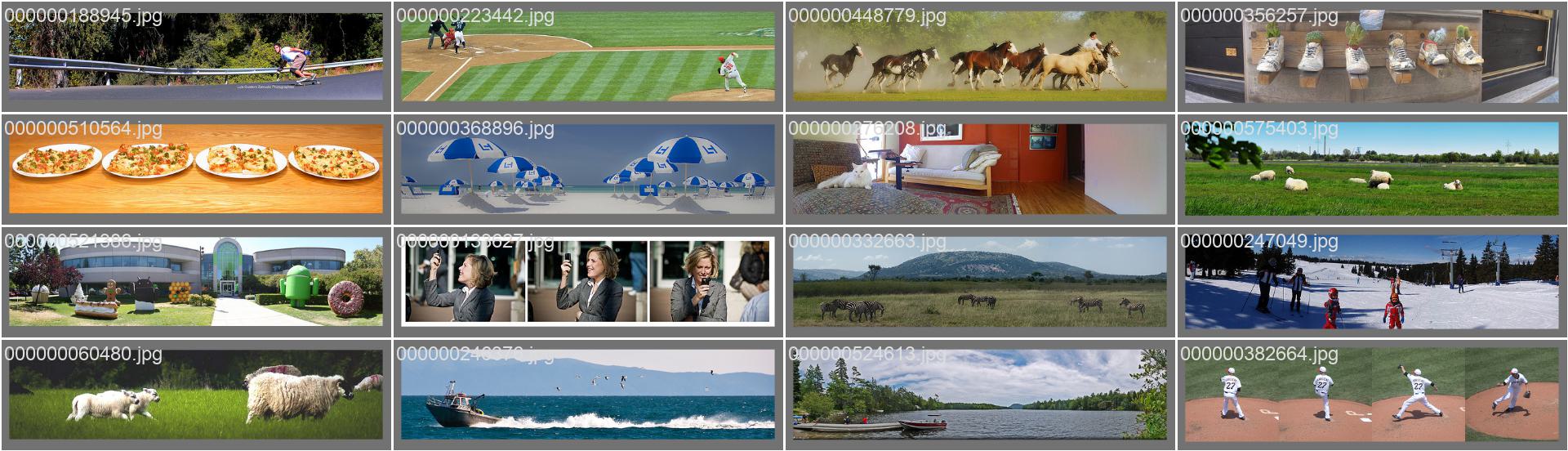

Supplement: S1 File — (ZIP) [file pone.0318578.s002.zip › suooprt information/pose/train30/val_batch1_labels.jpg]

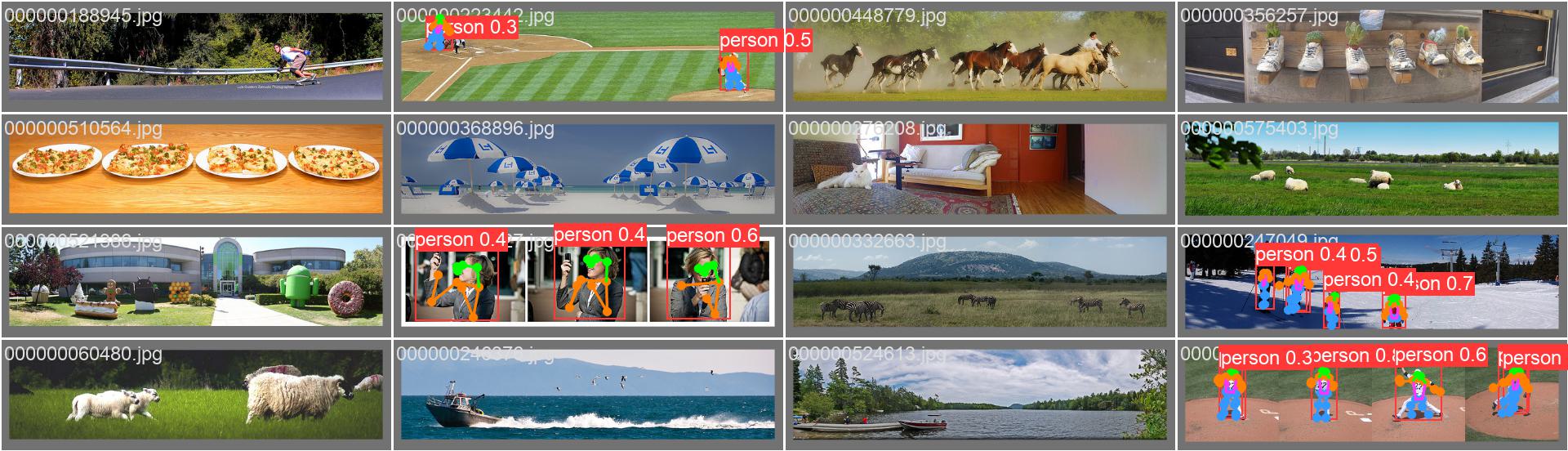

Supplement: S1 File — (ZIP) [file pone.0318578.s002.zip › suooprt information/pose/train30/val_batch1_pred.jpg]

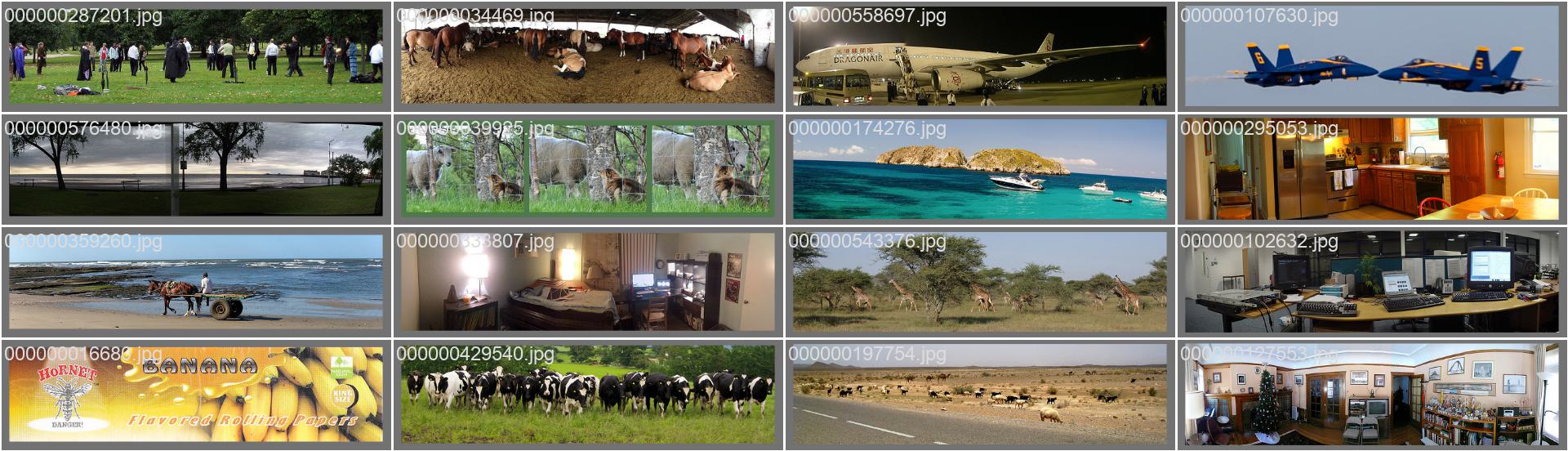

Supplement: S1 File — (ZIP) [file pone.0318578.s002.zip › suooprt information/pose/train30/val_batch2_labels.jpg]

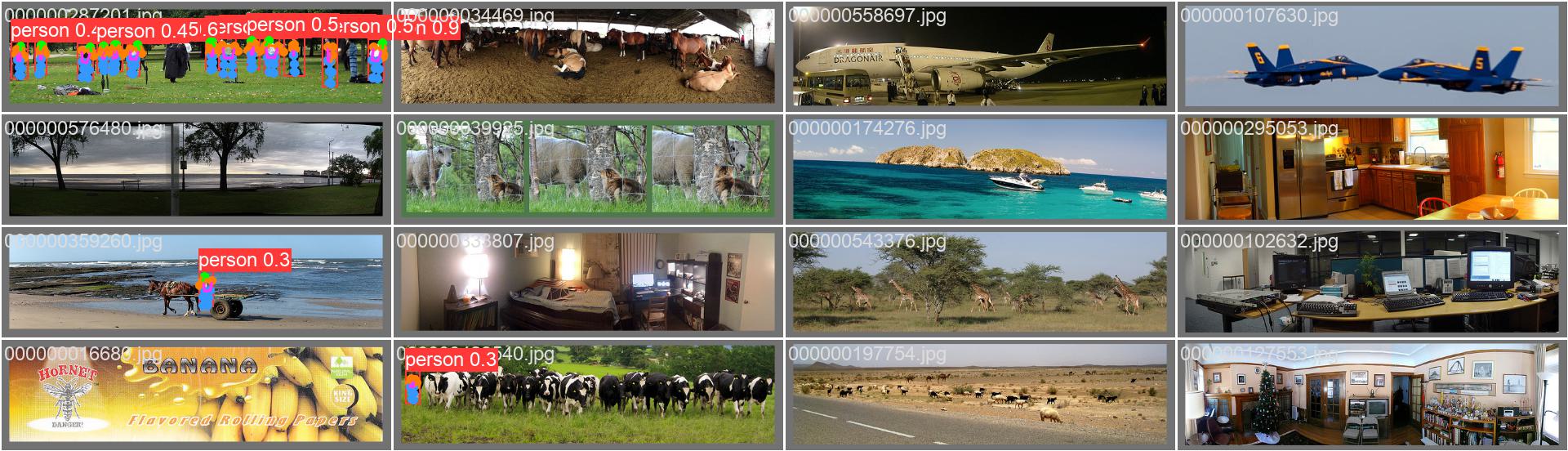

Supplement: S1 File — (ZIP) [file pone.0318578.s002.zip › suooprt information/pose/train30/val_batch2_pred.jpg]

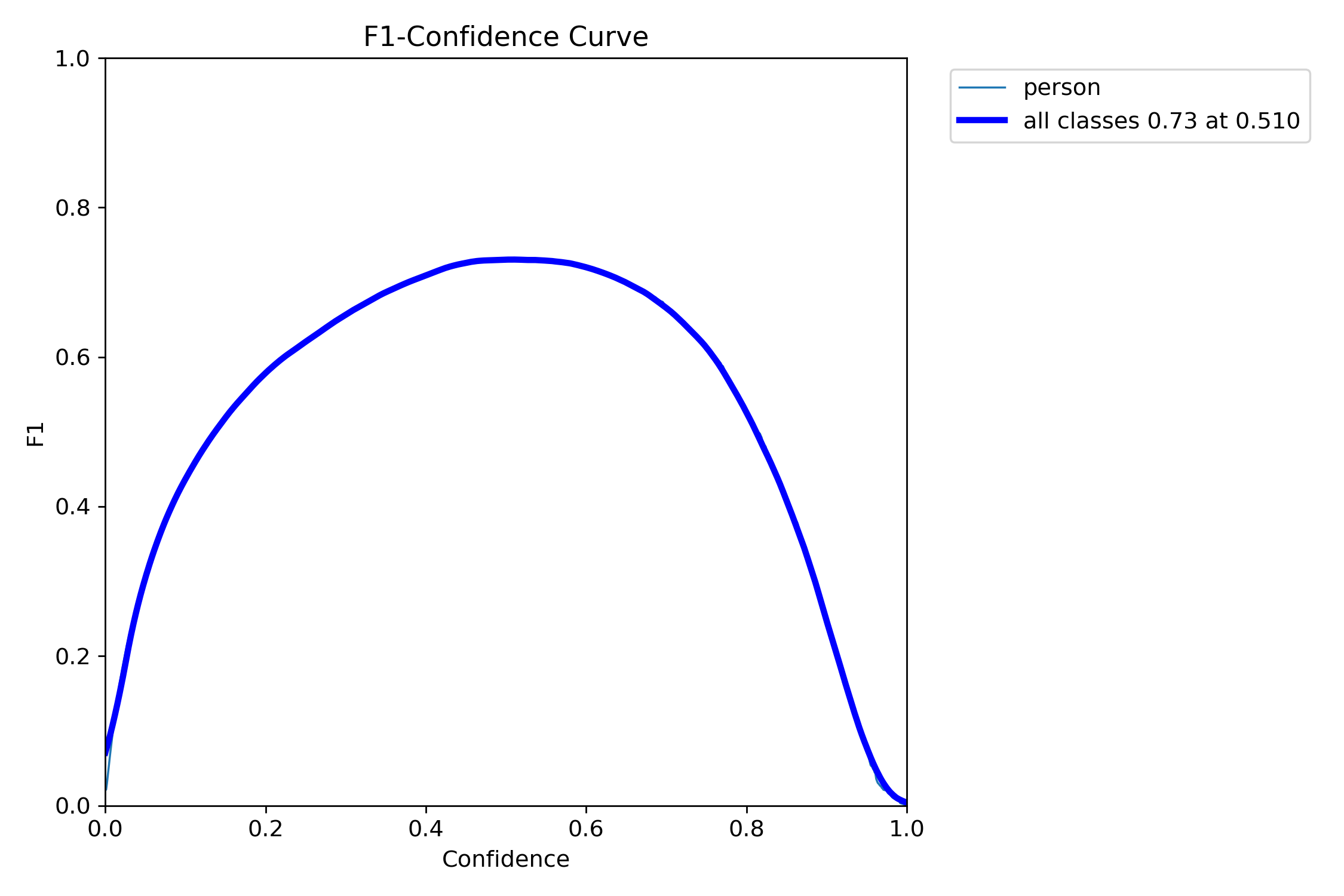

Supplement: S1 File — (ZIP) [file pone.0318578.s002.zip › suooprt information/pose/train31/BoxF1_curve.png]

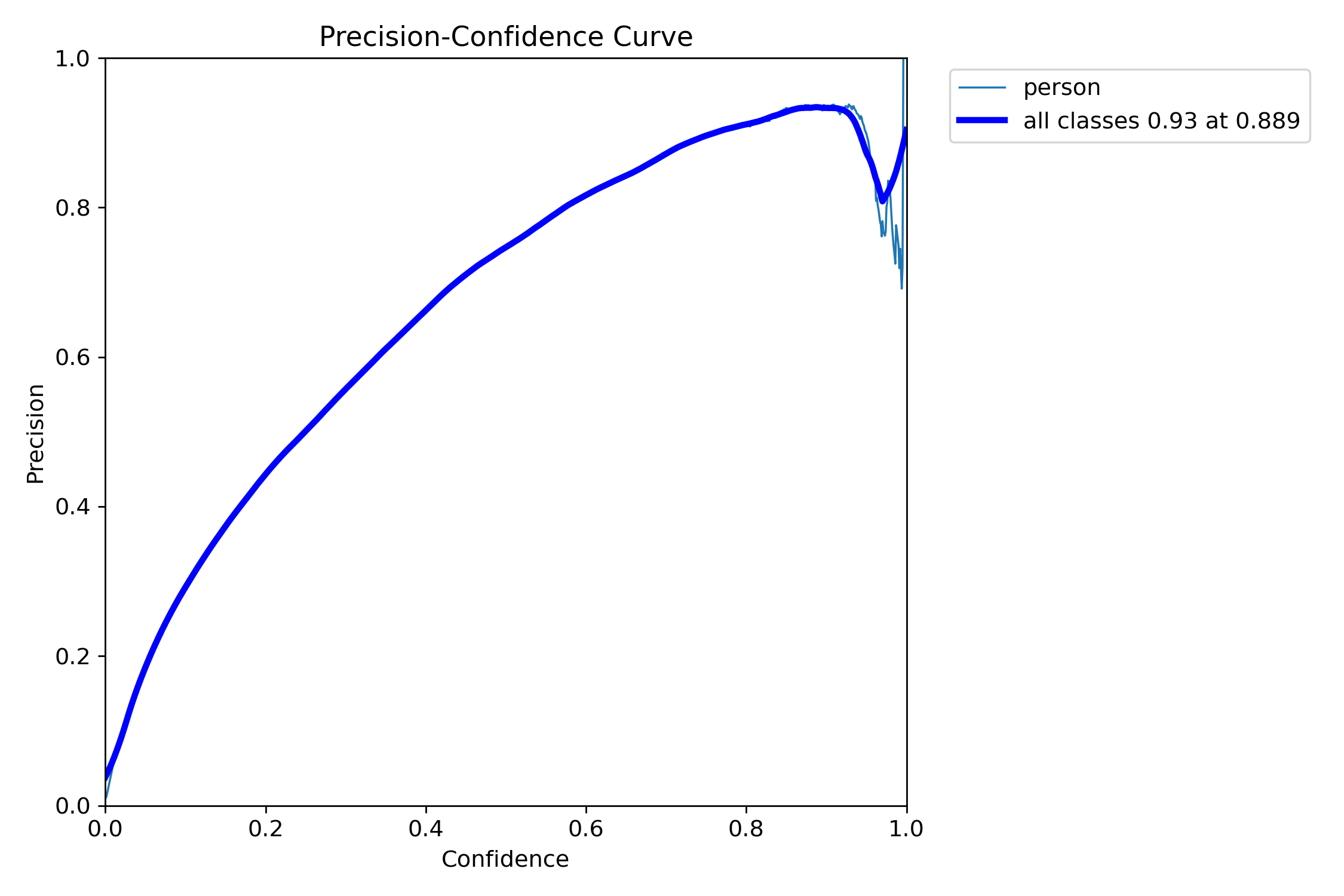

Supplement: S1 File — (ZIP) [file pone.0318578.s002.zip › suooprt information/pose/train31/BoxP_curve.png]

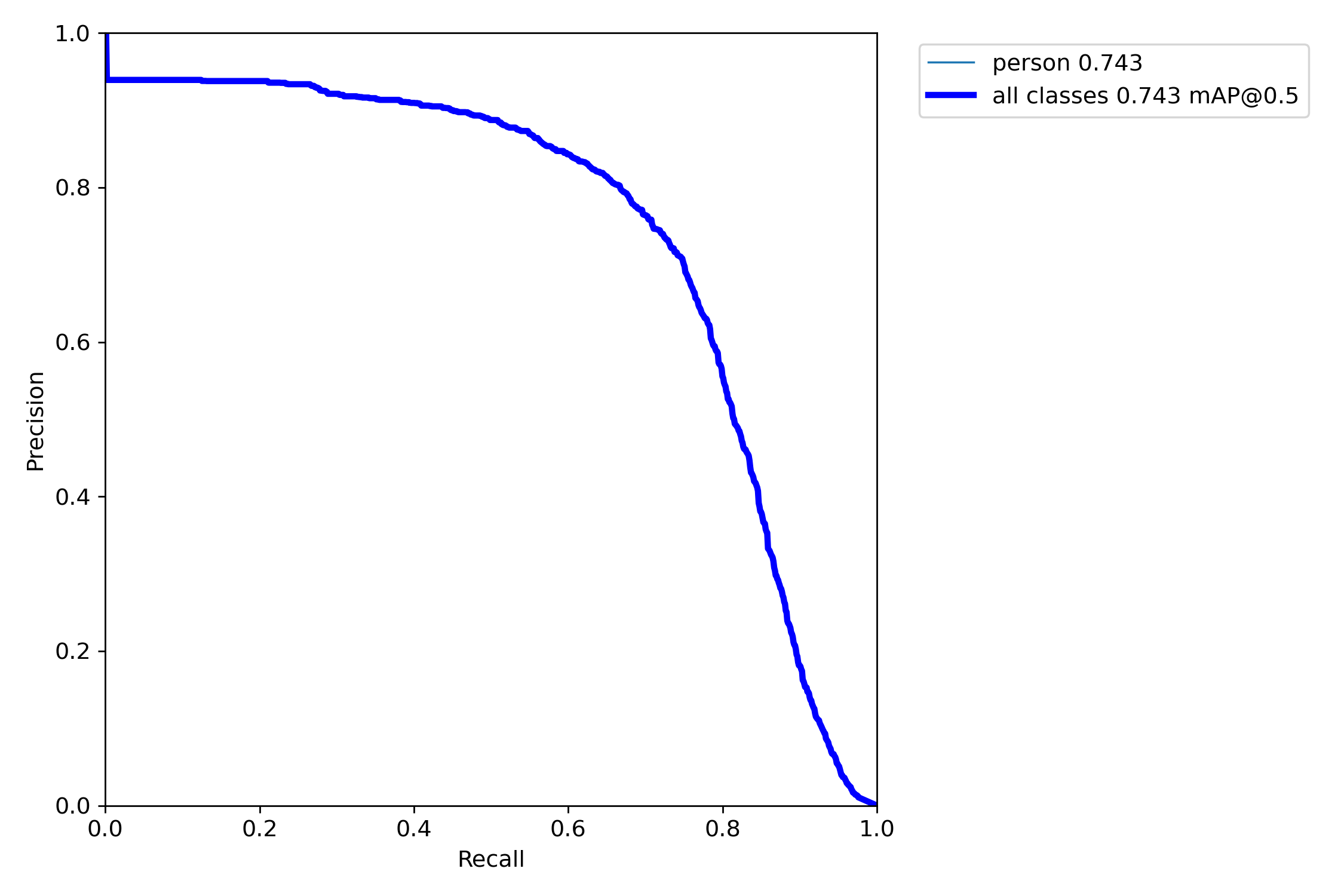

Supplement: S1 File — (ZIP) [file pone.0318578.s002.zip › suooprt information/pose/train31/BoxPR_curve.png]

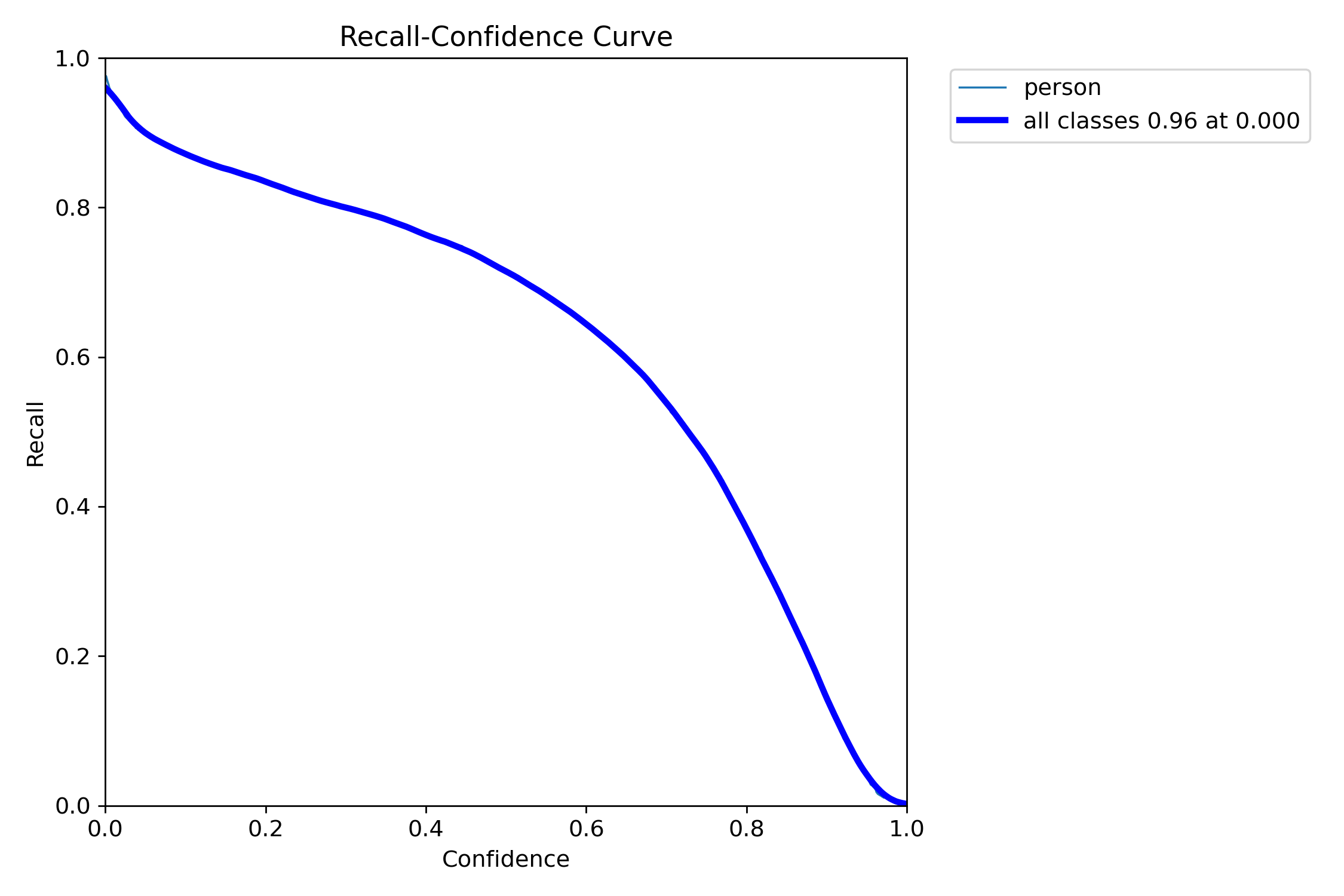

Supplement: S1 File — (ZIP) [file pone.0318578.s002.zip › suooprt information/pose/train31/BoxR_curve.png]

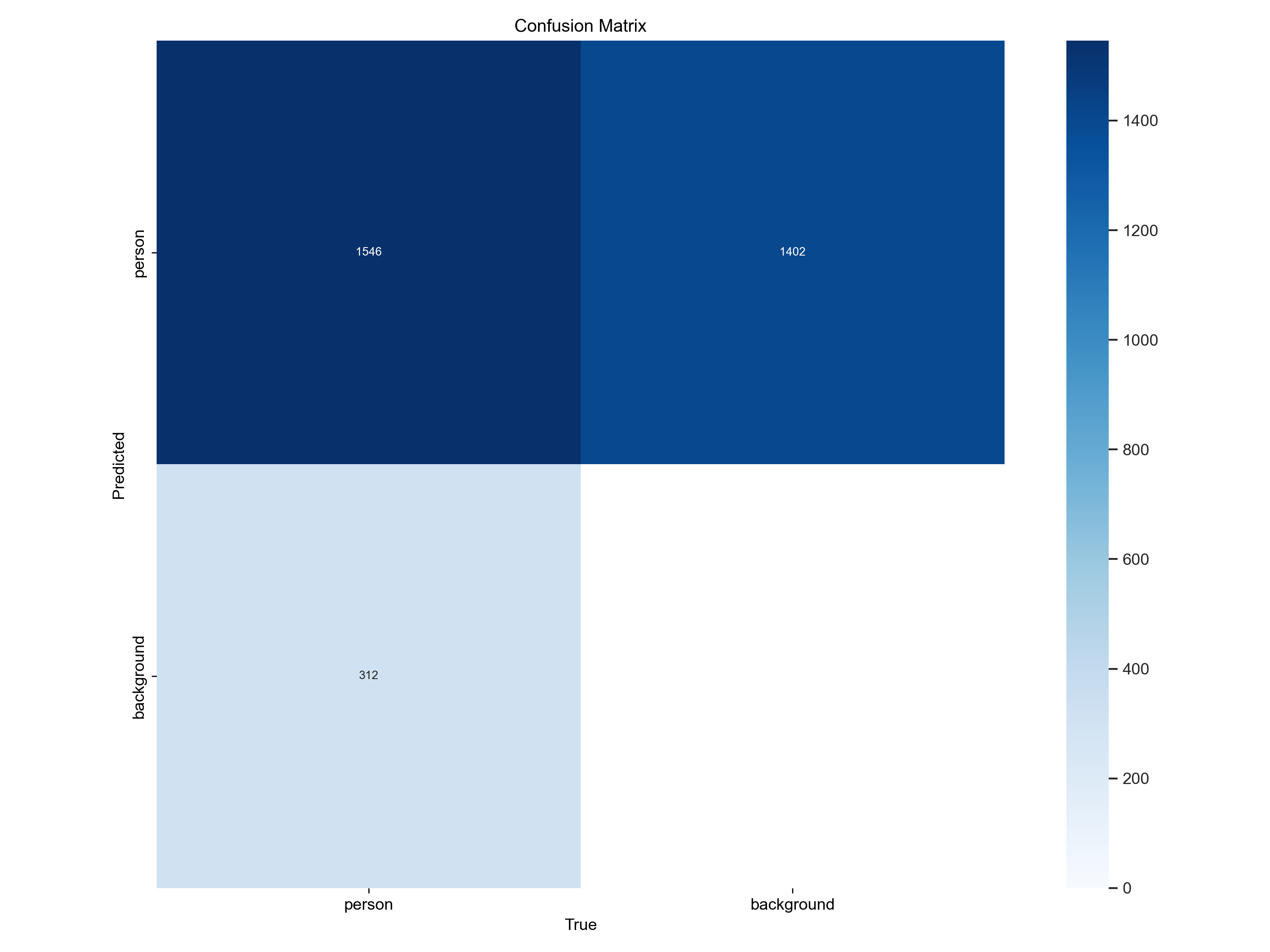

Supplement: S1 File — (ZIP) [file pone.0318578.s002.zip › suooprt information/pose/train31/confusion_matrix.png]

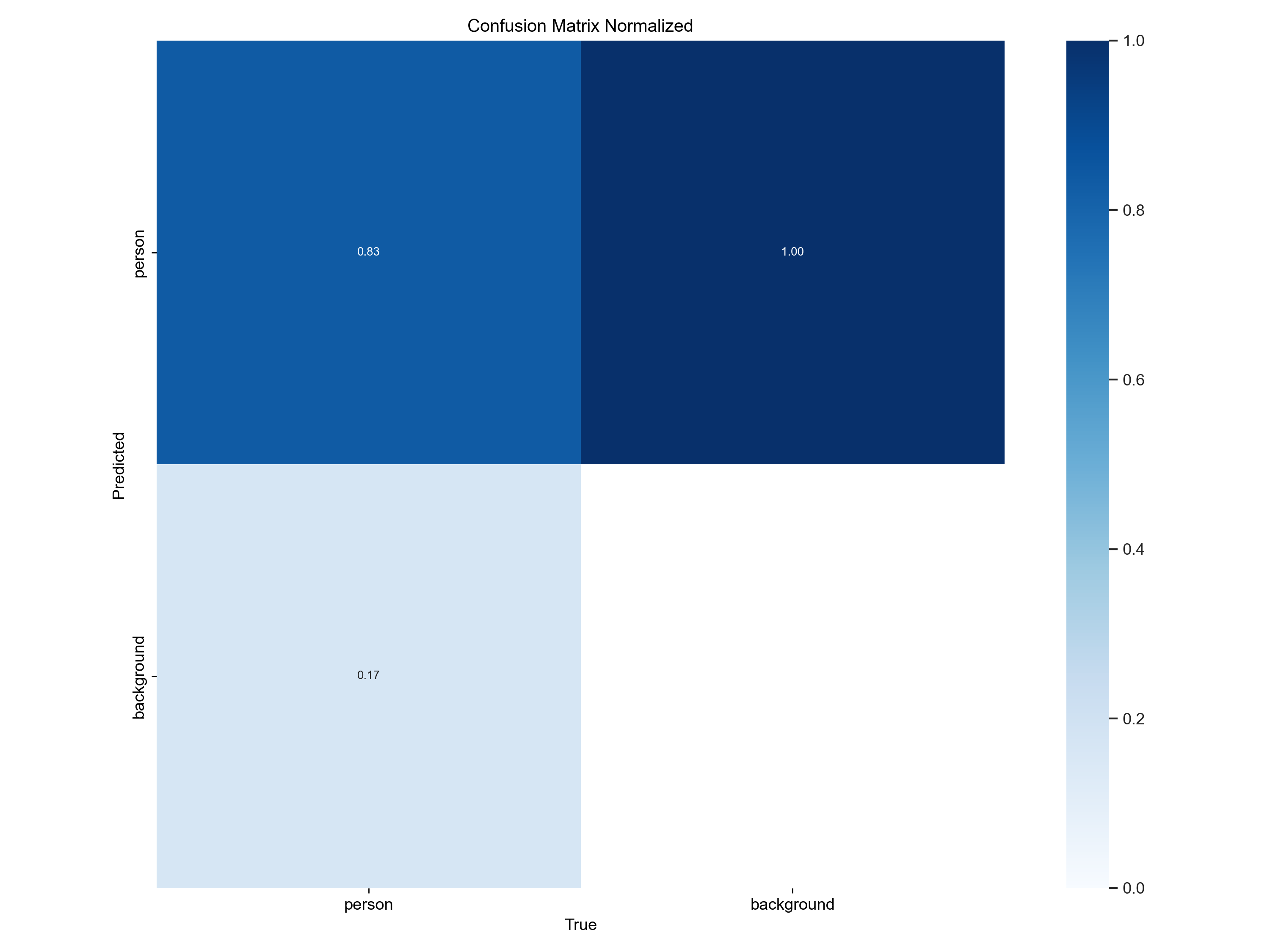

Supplement: S1 File — (ZIP) [file pone.0318578.s002.zip › suooprt information/pose/train31/confusion_matrix_normalized.png]

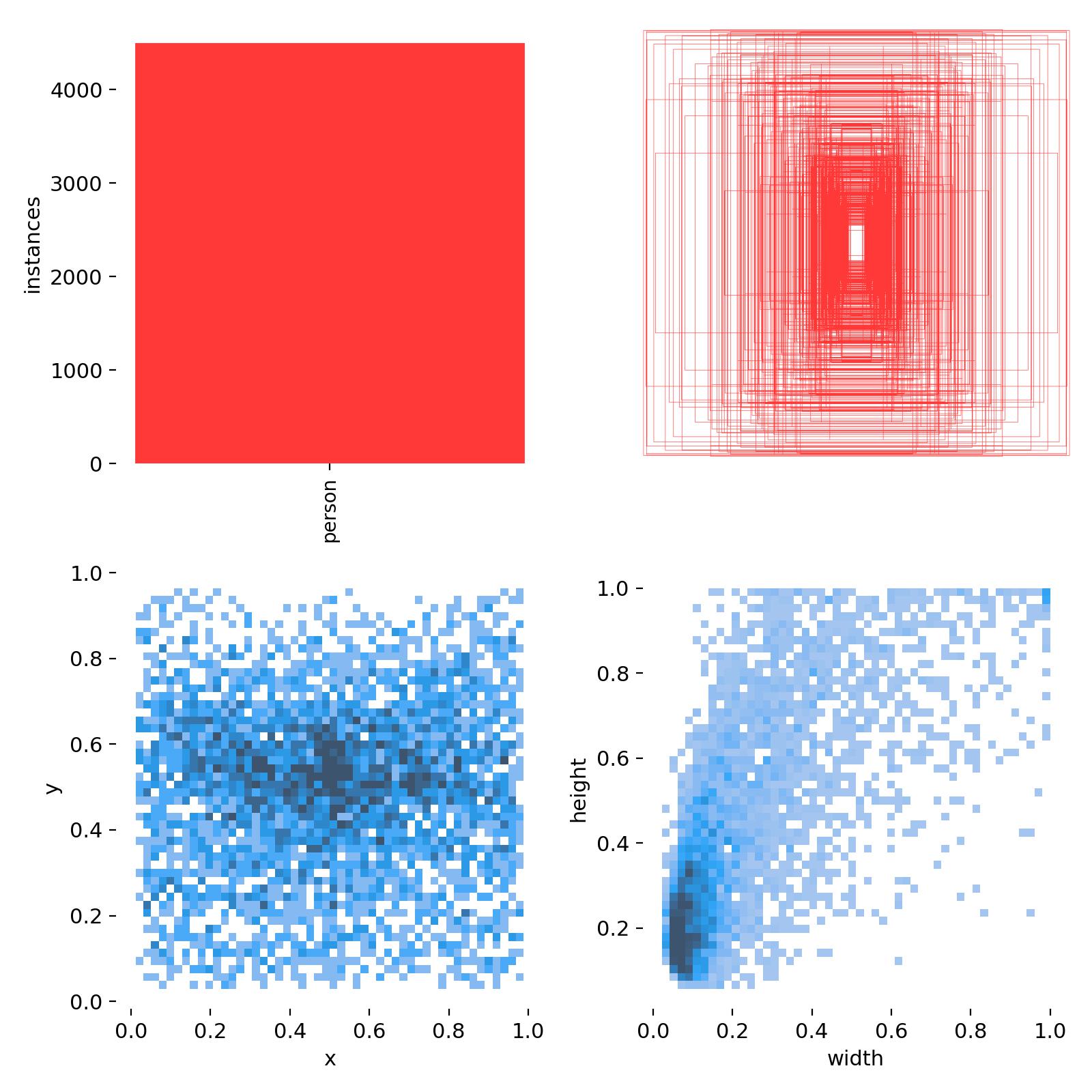

Supplement: S1 File — (ZIP) [file pone.0318578.s002.zip › suooprt information/pose/train31/labels.jpg]

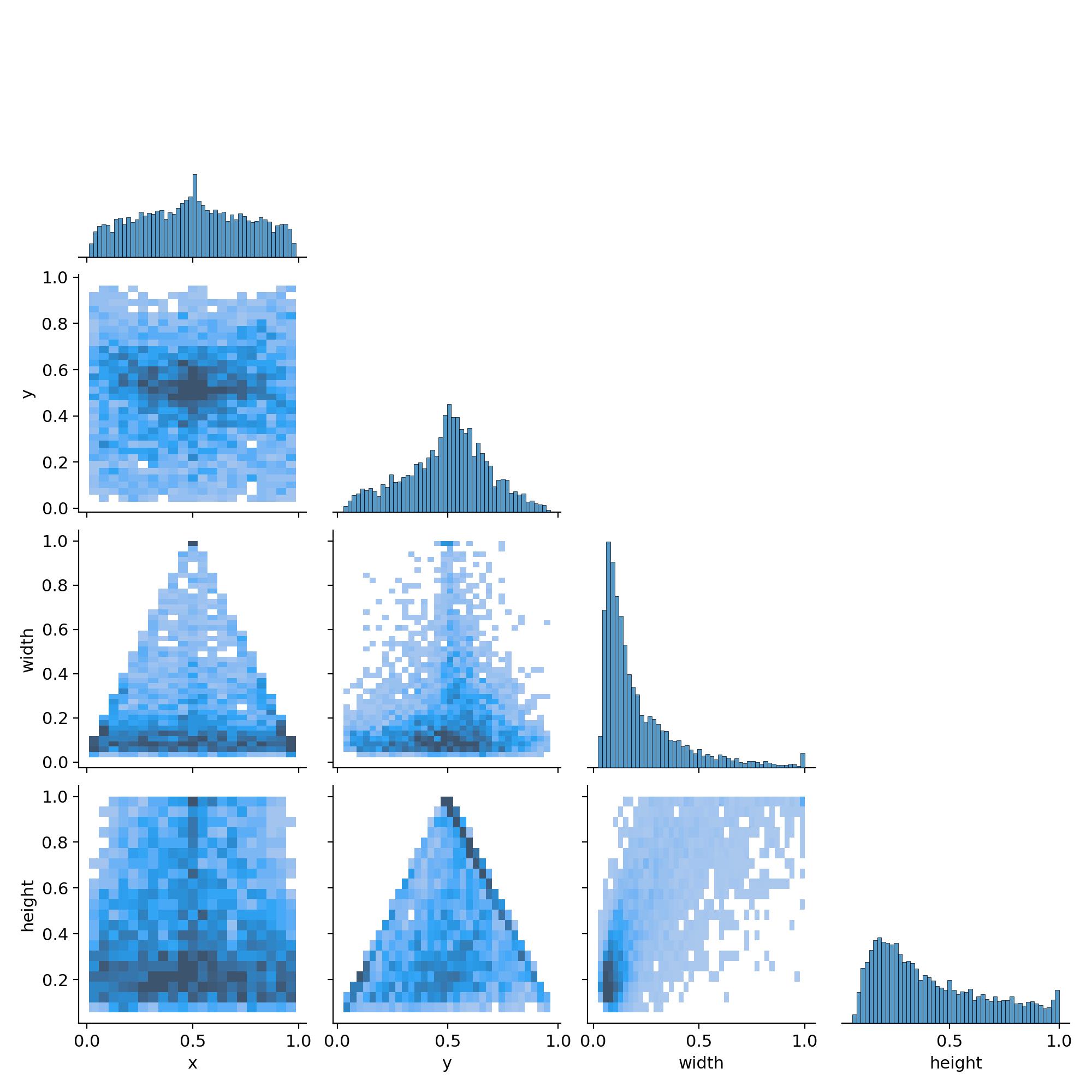

Supplement: S1 File — (ZIP) [file pone.0318578.s002.zip › suooprt information/pose/train31/labels_correlogram.jpg]

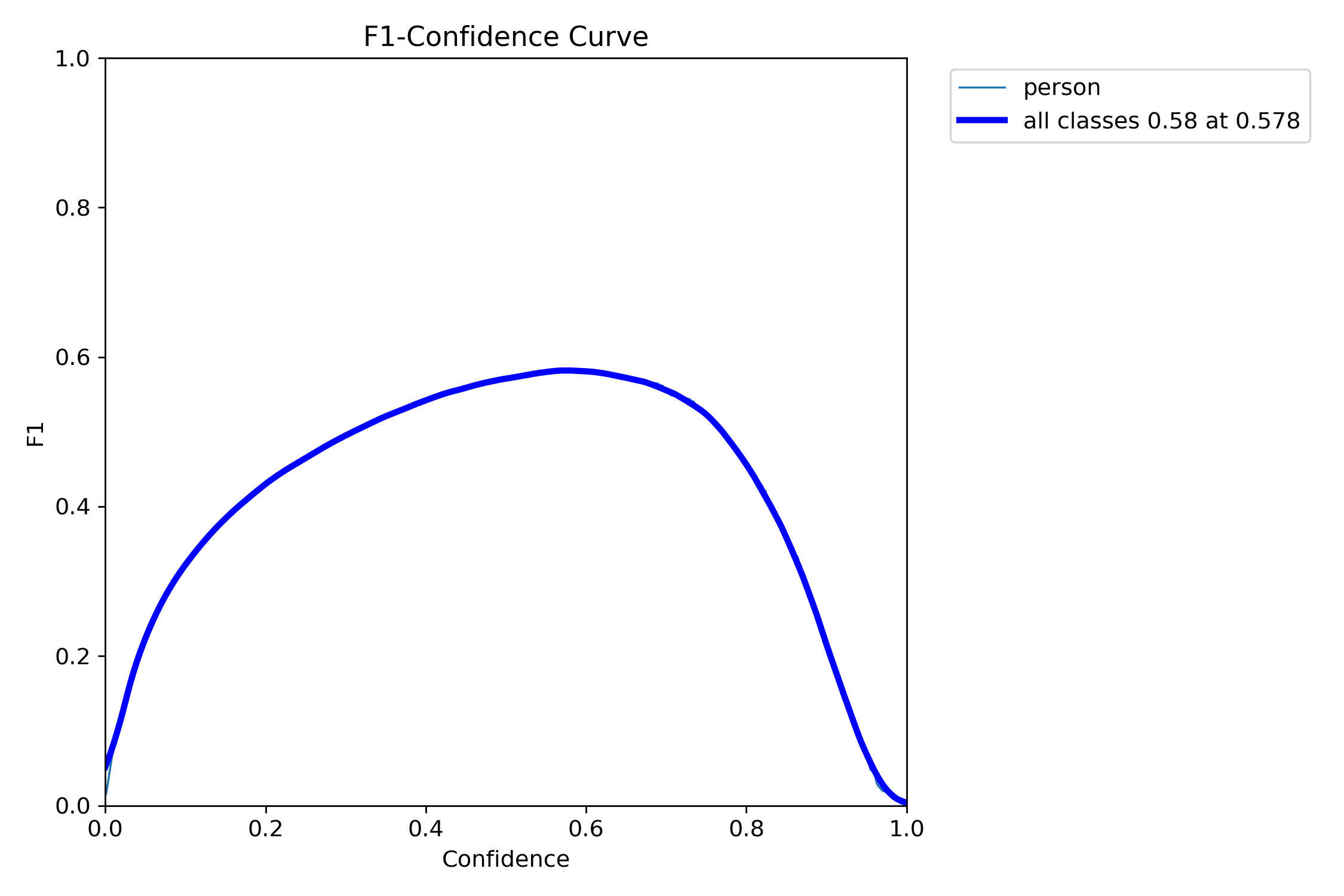

Supplement: S1 File — (ZIP) [file pone.0318578.s002.zip › suooprt information/pose/train31/PoseF1_curve.png]

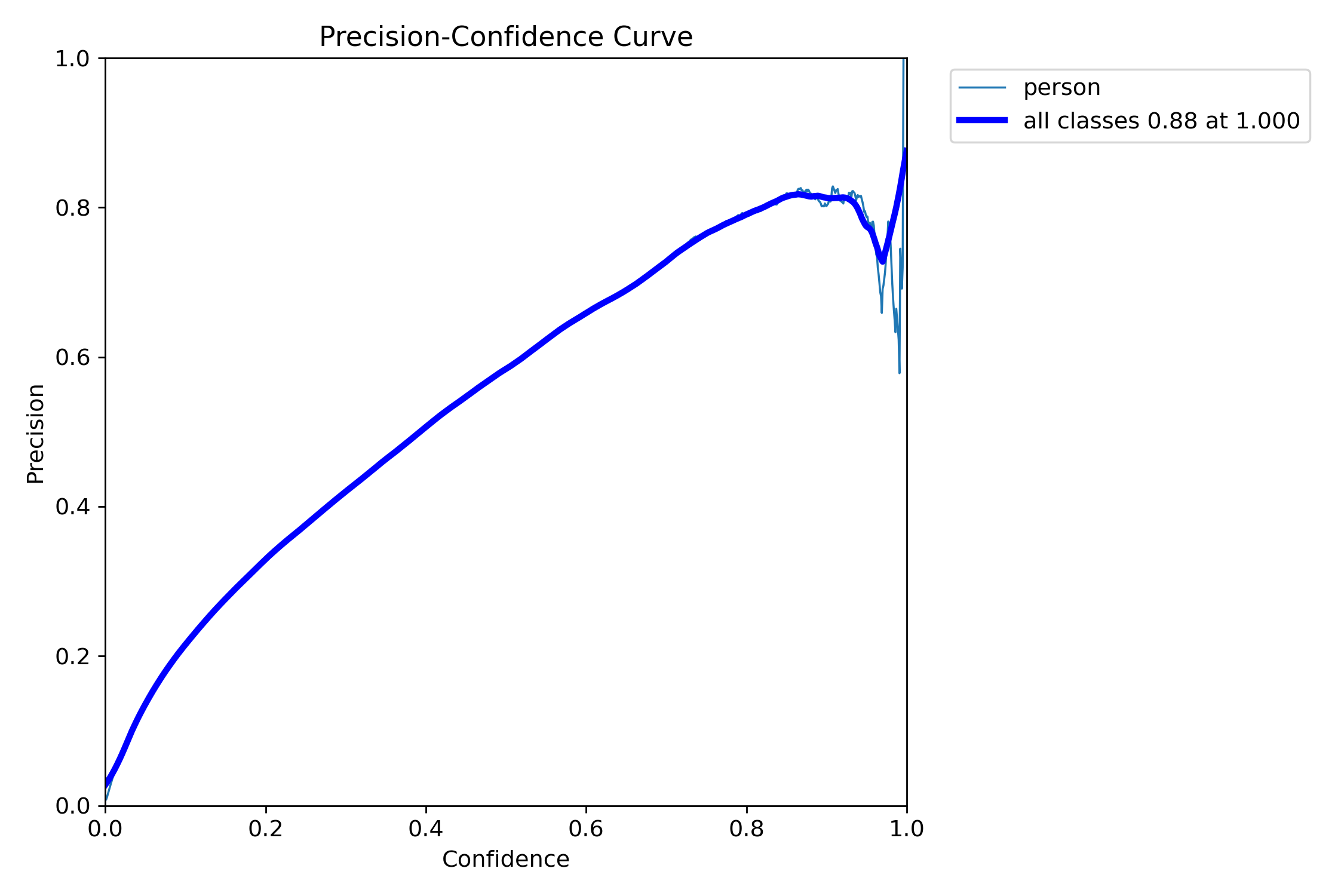

Supplement: S1 File — (ZIP) [file pone.0318578.s002.zip › suooprt information/pose/train31/PoseP_curve.png]

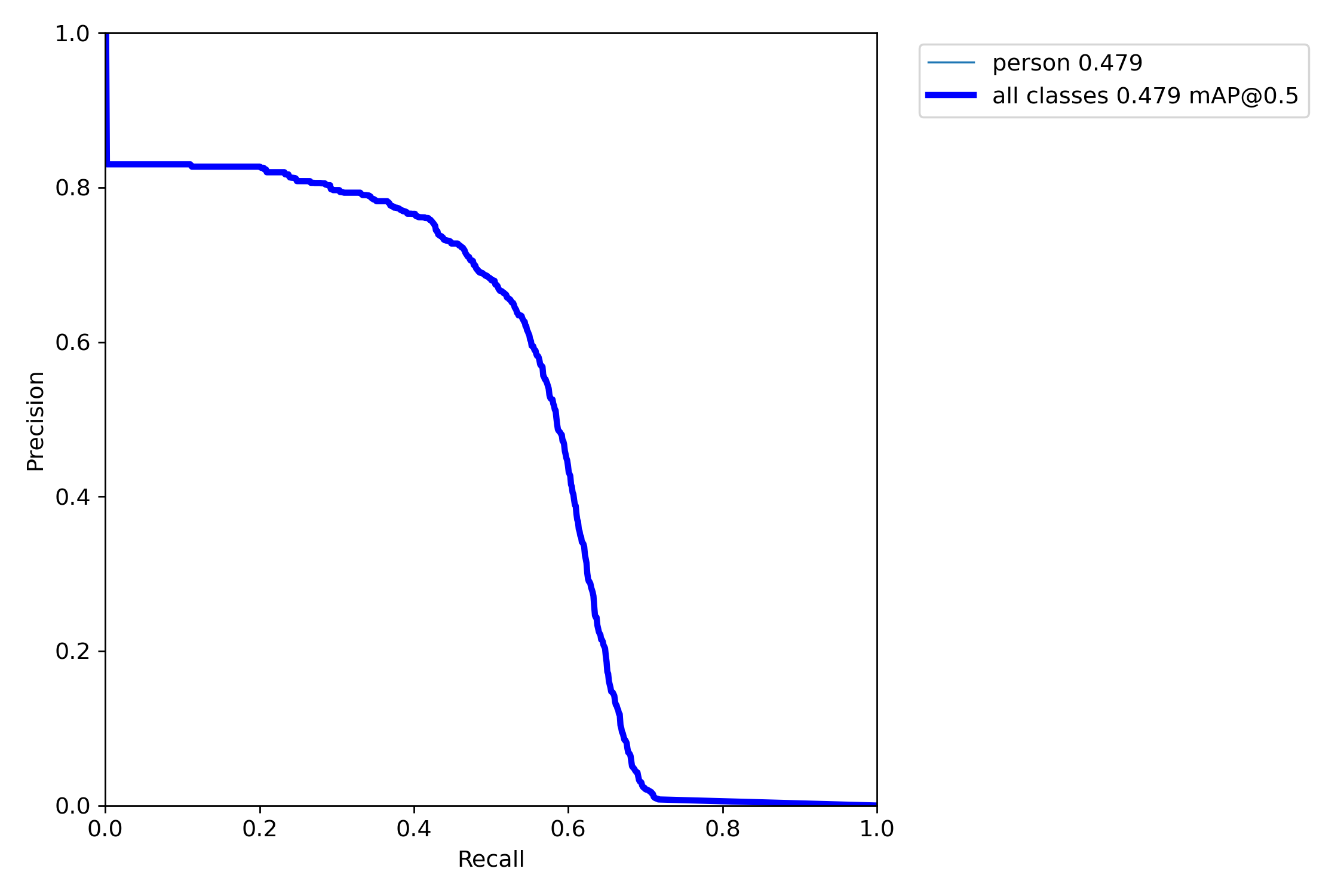

Supplement: S1 File — (ZIP) [file pone.0318578.s002.zip › suooprt information/pose/train31/PosePR_curve.png]

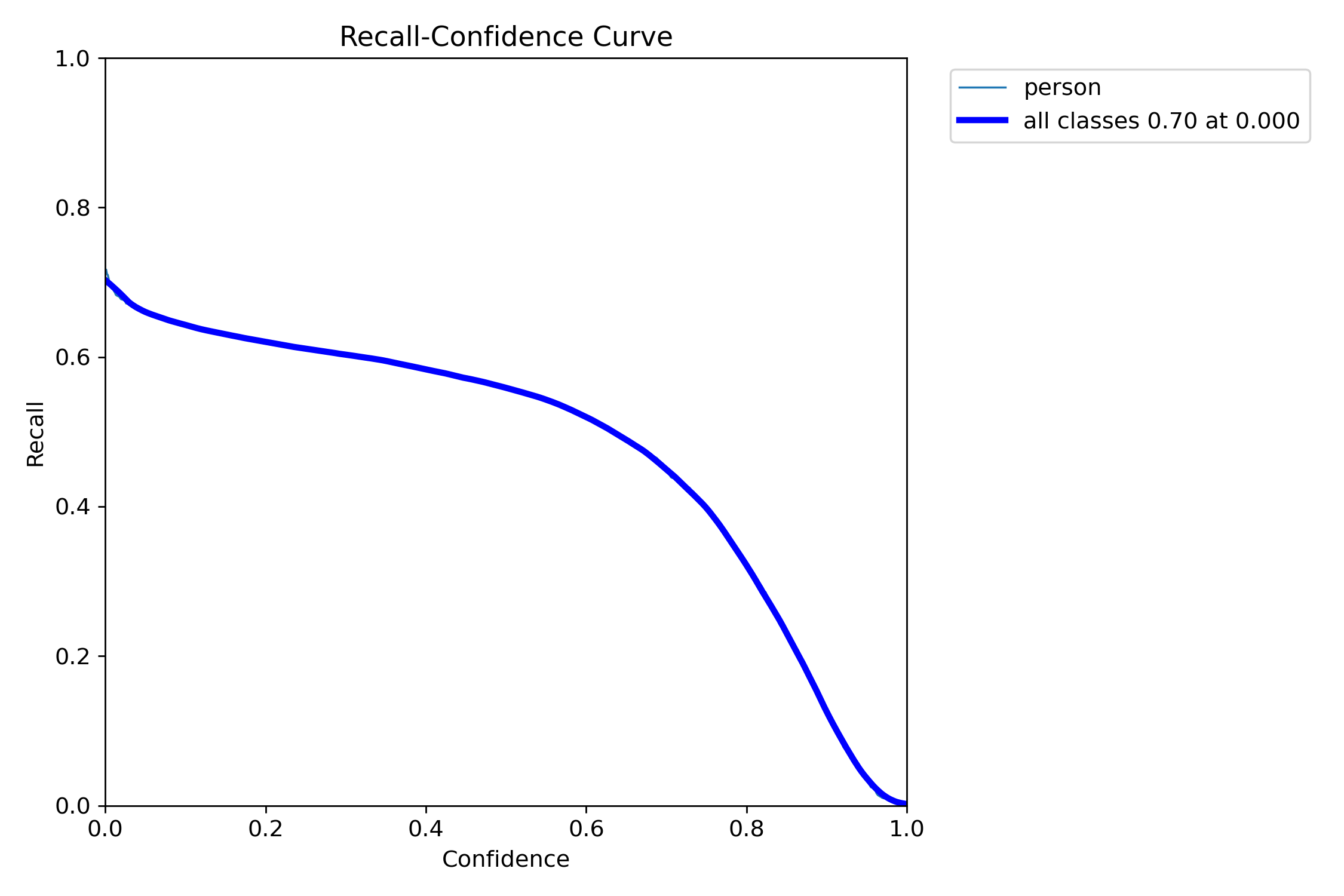

Supplement: S1 File — (ZIP) [file pone.0318578.s002.zip › suooprt information/pose/train31/PoseR_curve.png]

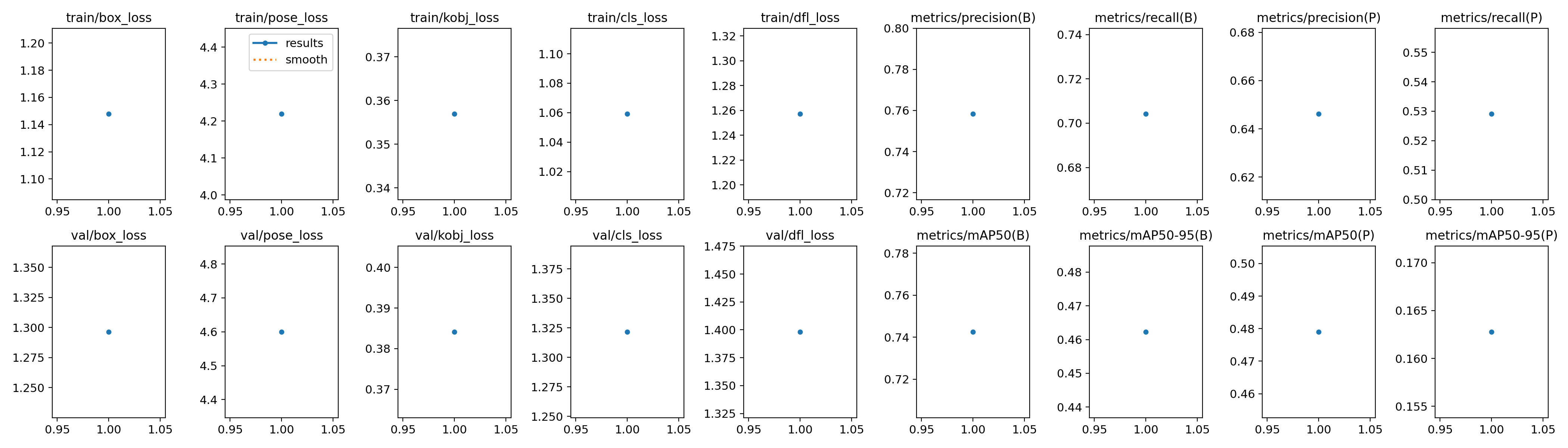

Supplement: S1 File — (ZIP) [file pone.0318578.s002.zip › suooprt information/pose/train31/results.png]

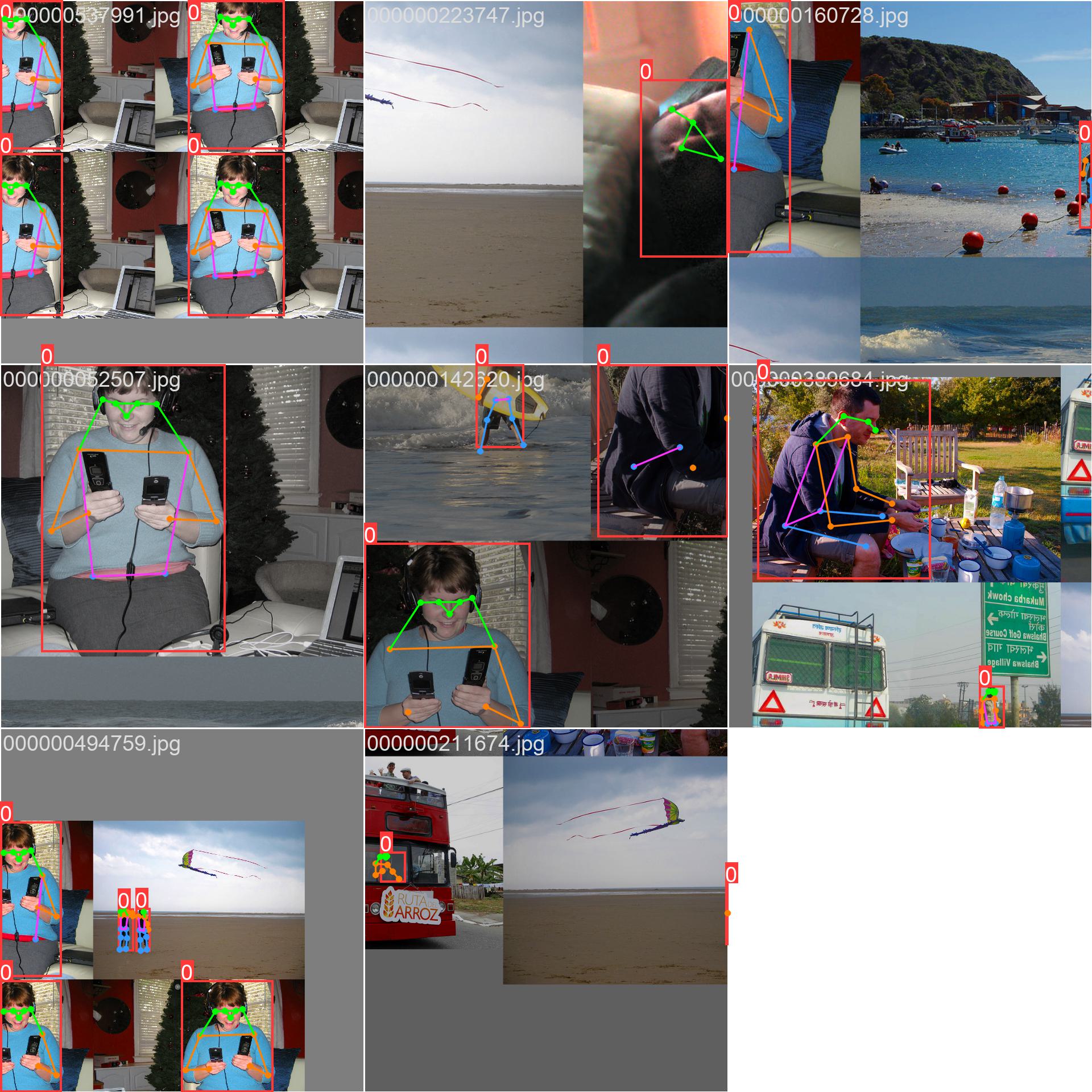

Supplement: S1 File — (ZIP) [file pone.0318578.s002.zip › suooprt information/pose/train31/train_batch0.jpg]

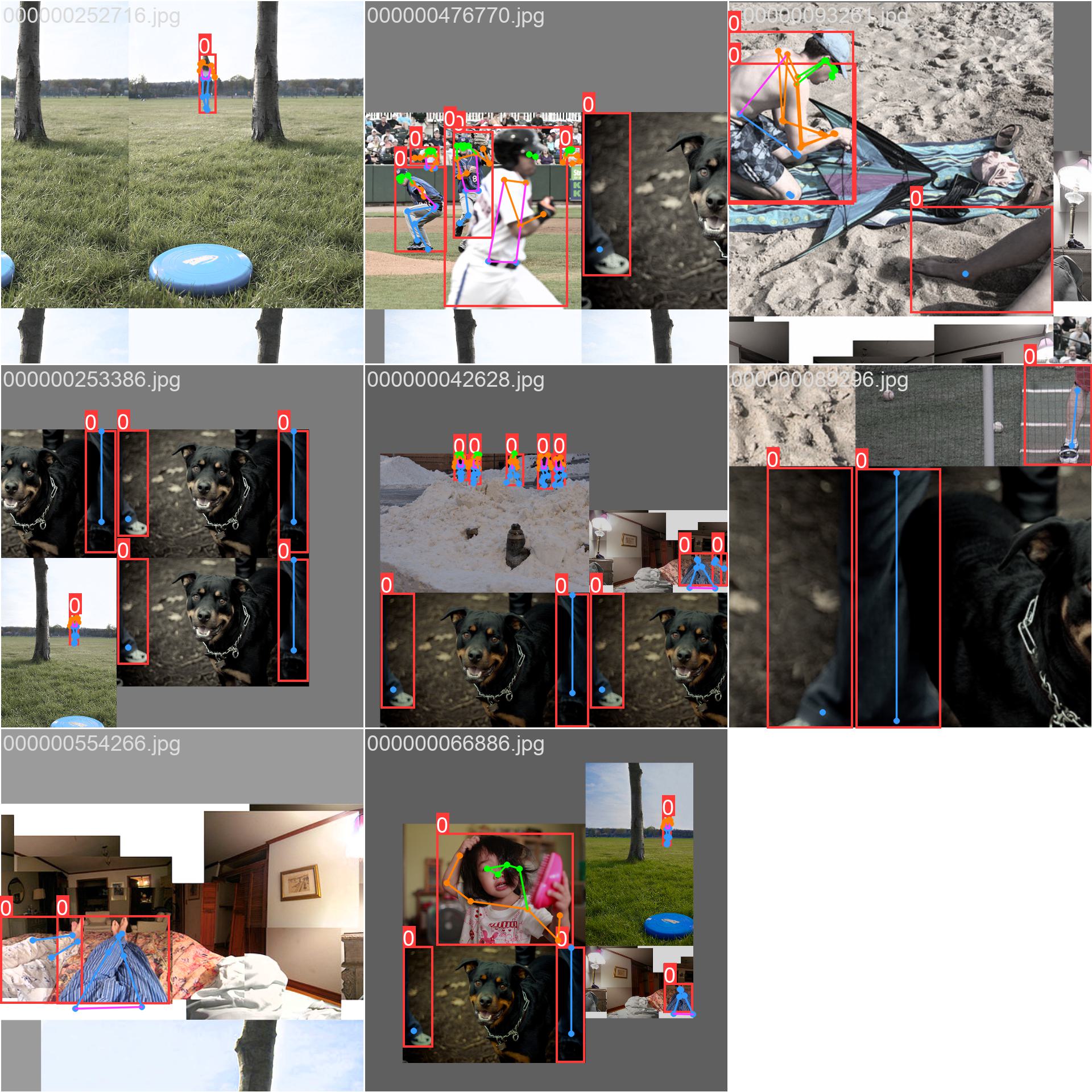

Supplement: S1 File — (ZIP) [file pone.0318578.s002.zip › suooprt information/pose/train31/train_batch1.jpg]

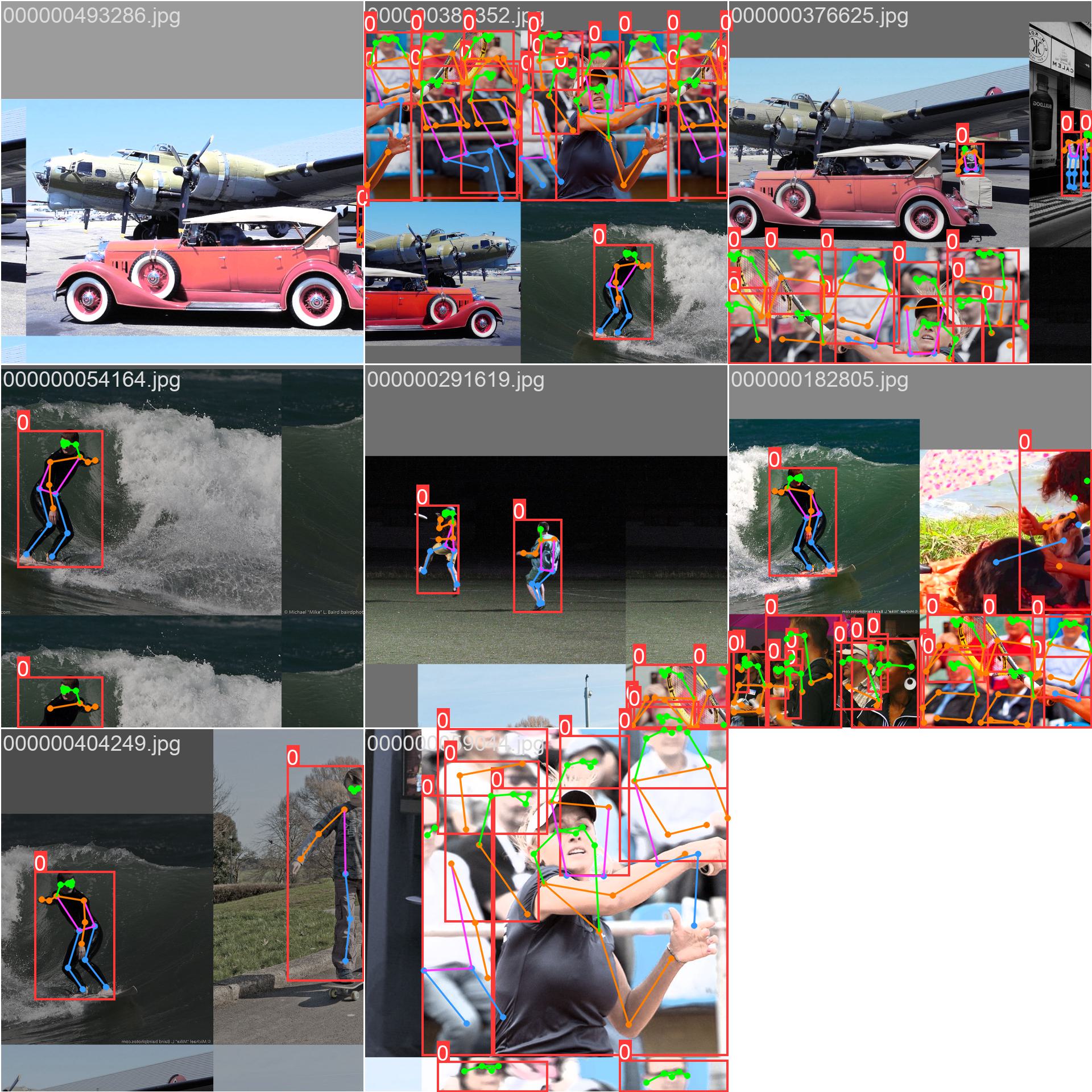

Supplement: S1 File — (ZIP) [file pone.0318578.s002.zip › suooprt information/pose/train31/train_batch2.jpg]

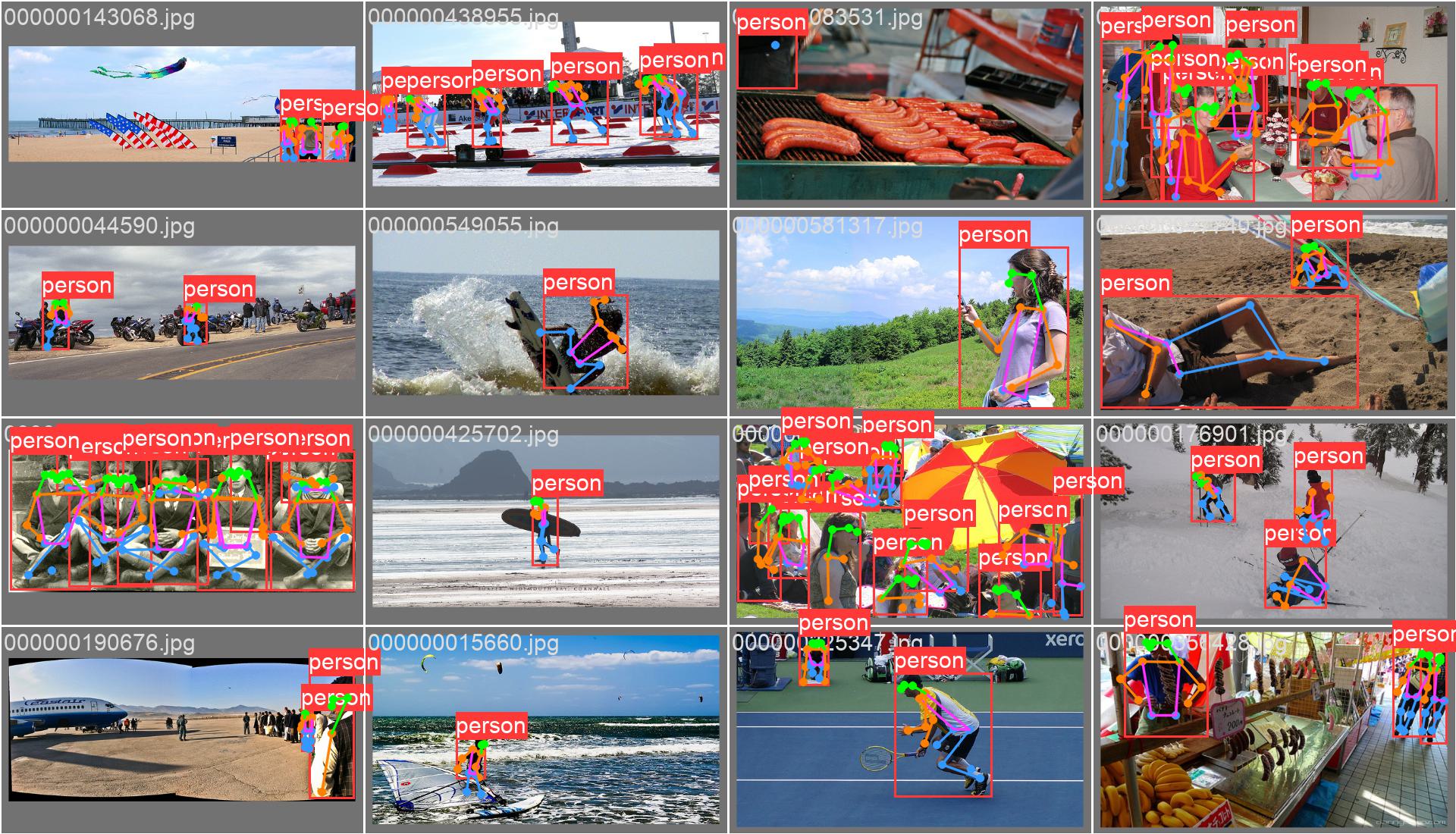

Supplement: S1 File — (ZIP) [file pone.0318578.s002.zip › suooprt information/pose/train31/val_batch0_labels.jpg]

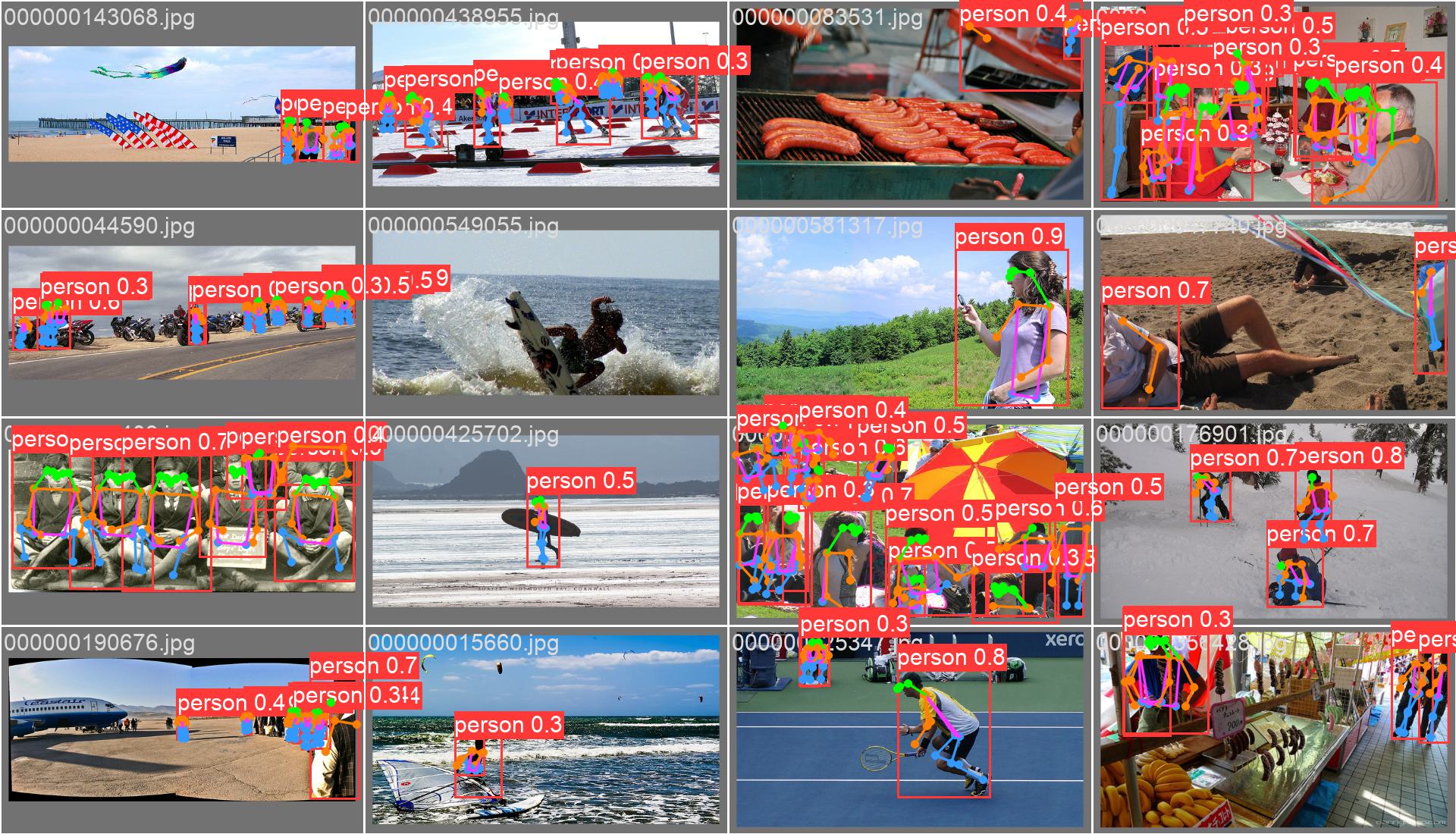

Supplement: S1 File — (ZIP) [file pone.0318578.s002.zip › suooprt information/pose/train31/val_batch0_pred.jpg]

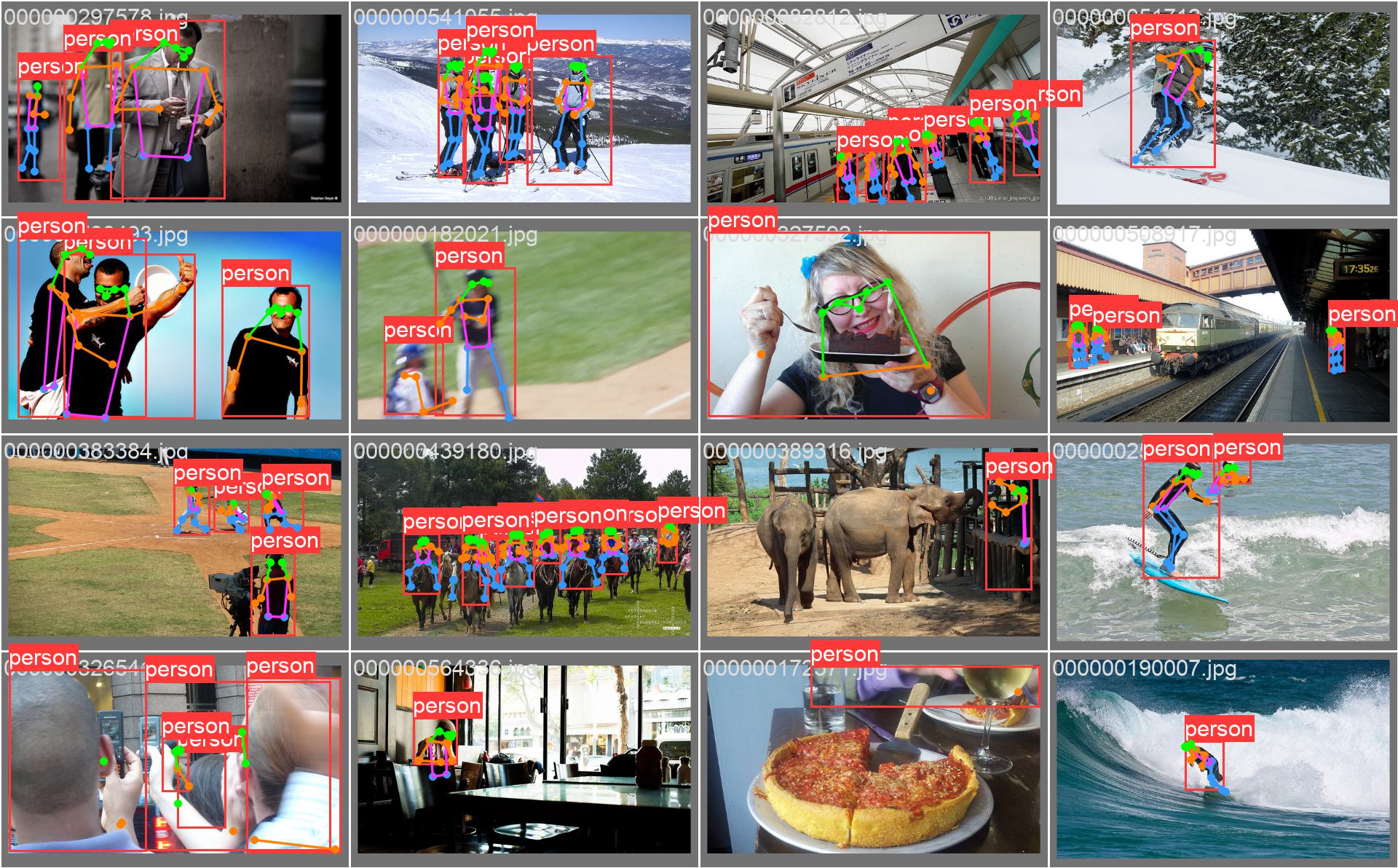

Supplement: S1 File — (ZIP) [file pone.0318578.s002.zip › suooprt information/pose/train31/val_batch1_labels.jpg]

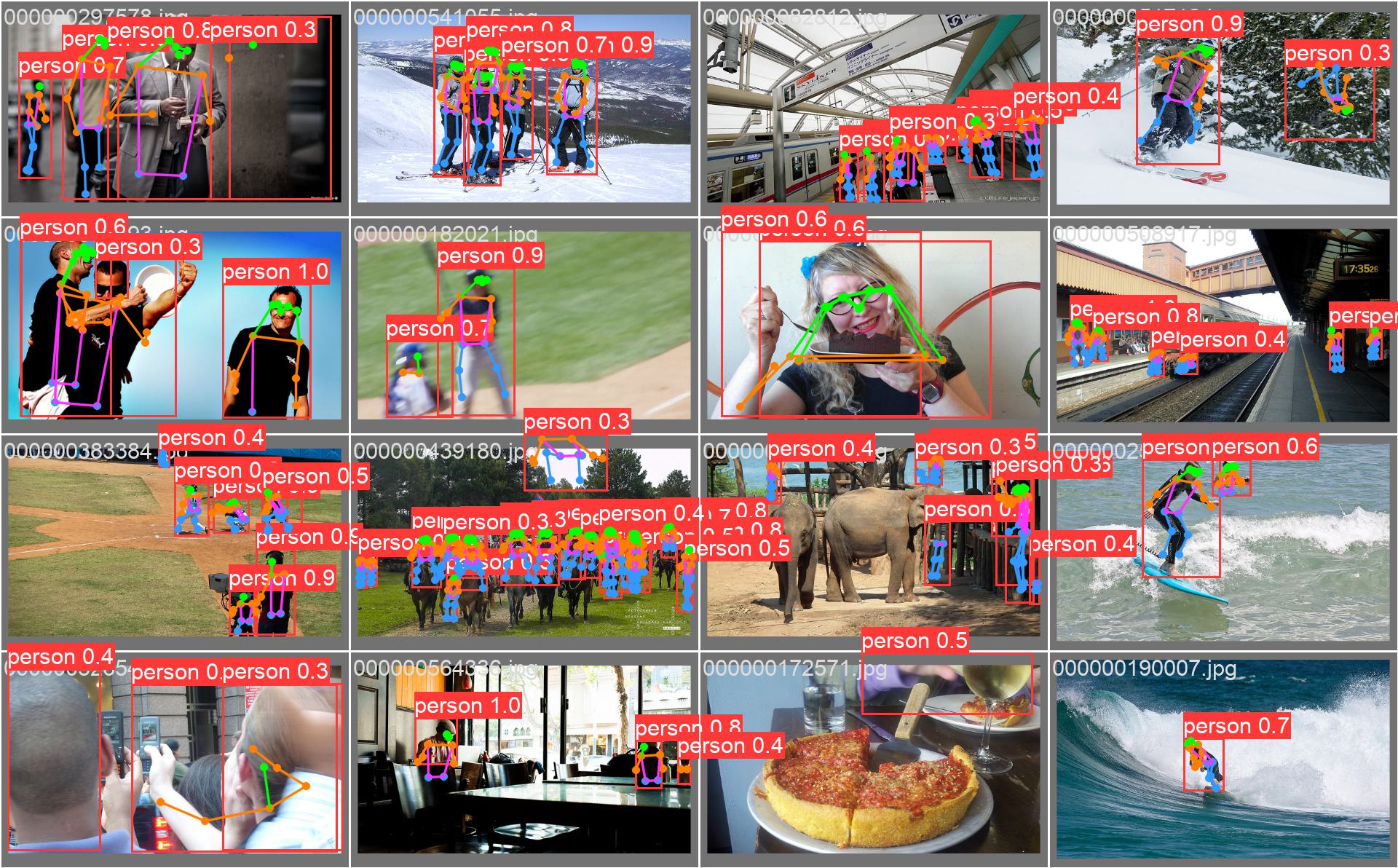

Supplement: S1 File — (ZIP) [file pone.0318578.s002.zip › suooprt information/pose/train31/val_batch1_pred.jpg]

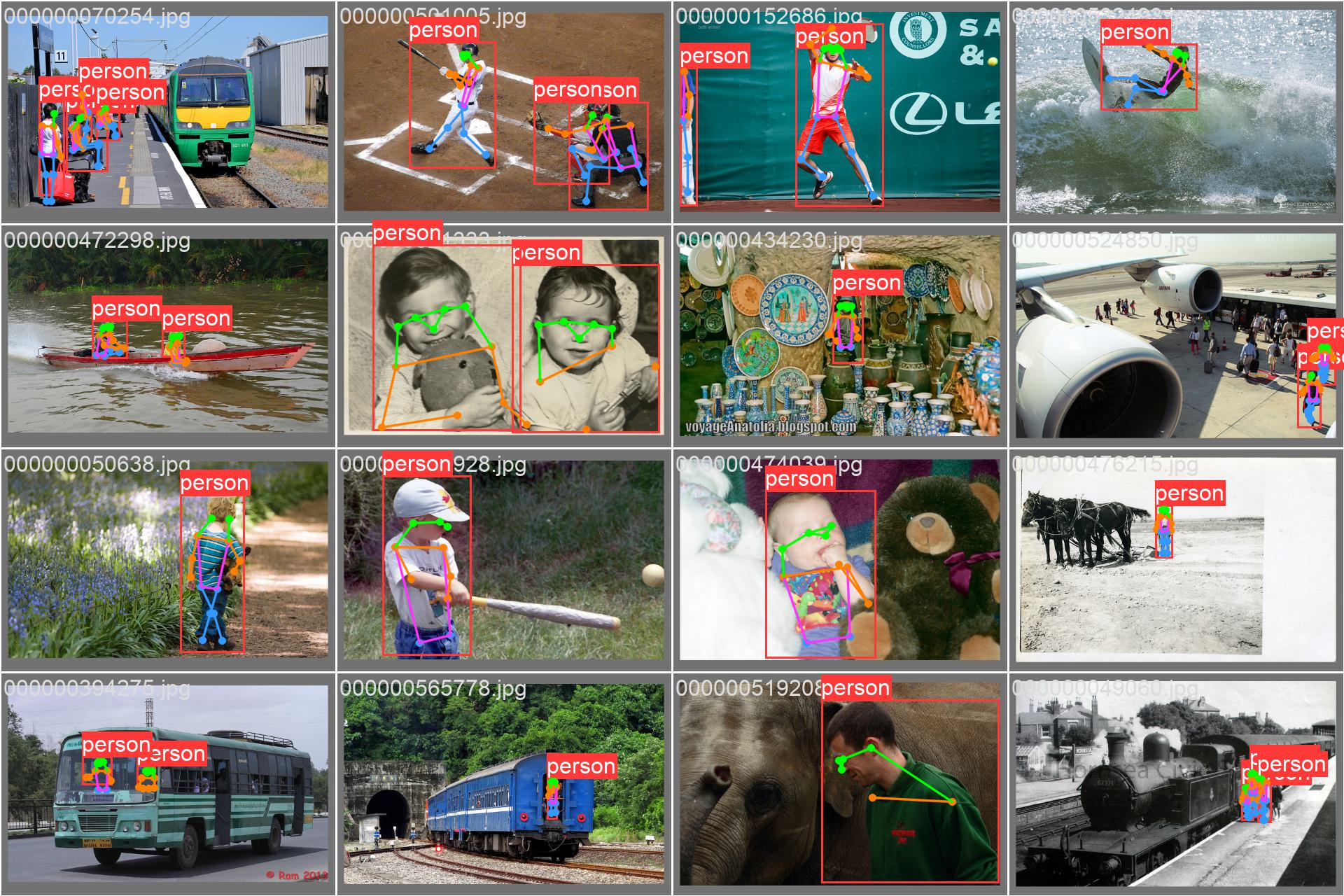

Supplement: S1 File — (ZIP) [file pone.0318578.s002.zip › suooprt information/pose/train31/val_batch2_labels.jpg]

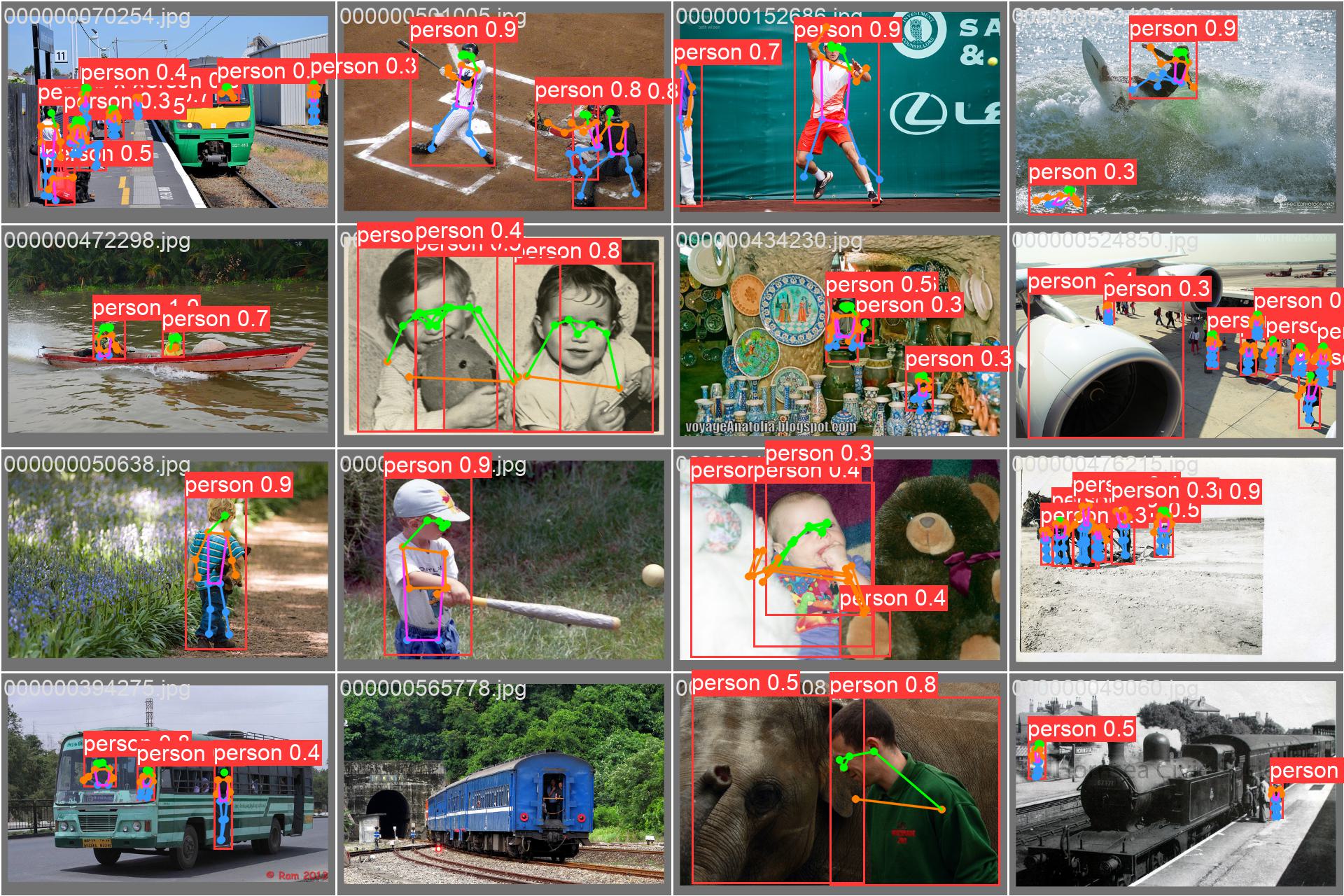

Supplement: S1 File — (ZIP) [file pone.0318578.s002.zip › suooprt information/pose/train31/val_batch2_pred.jpg]

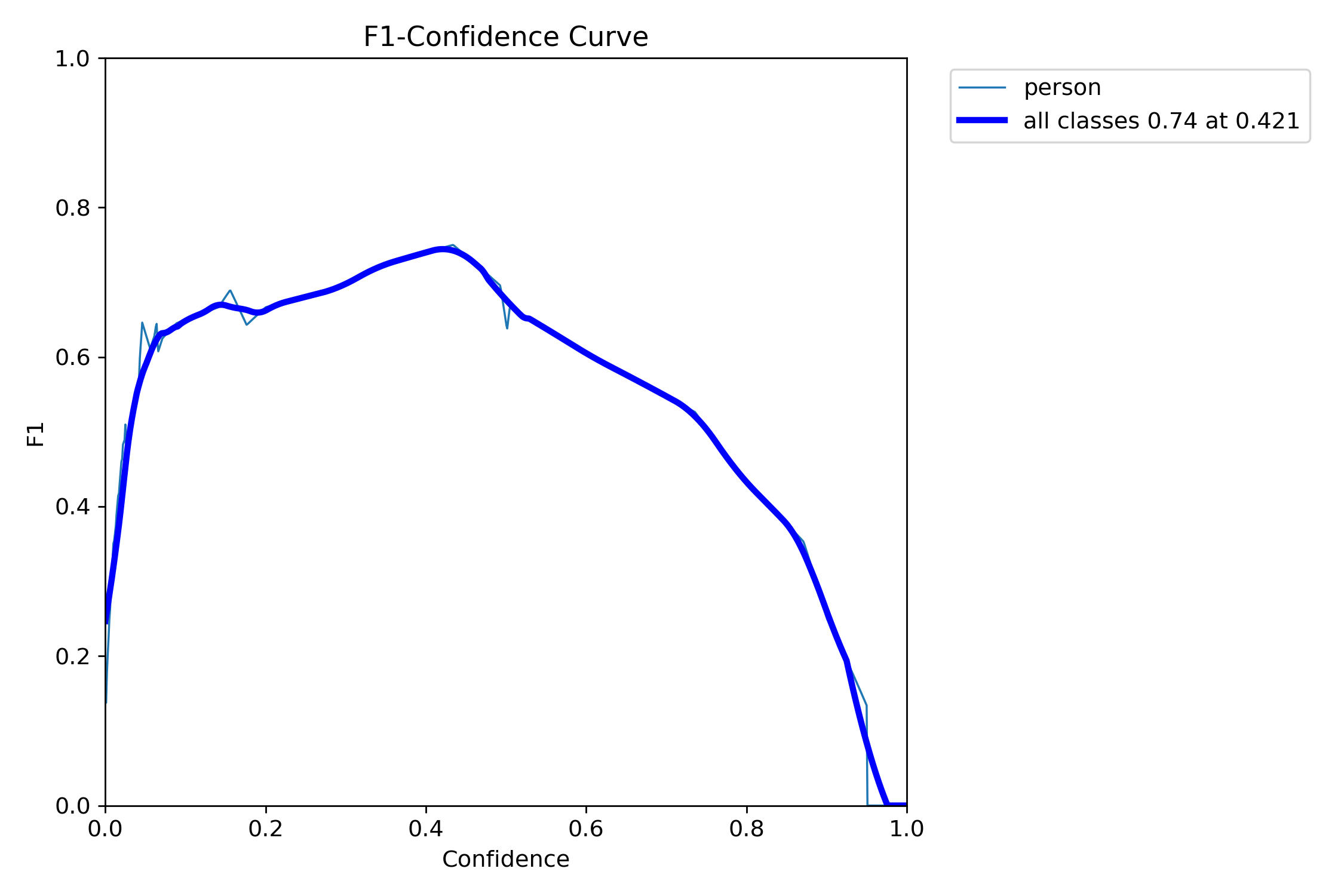

Supplement: S1 File — (ZIP) [file pone.0318578.s002.zip › suooprt information/pose/train32/BoxF1_curve.png]

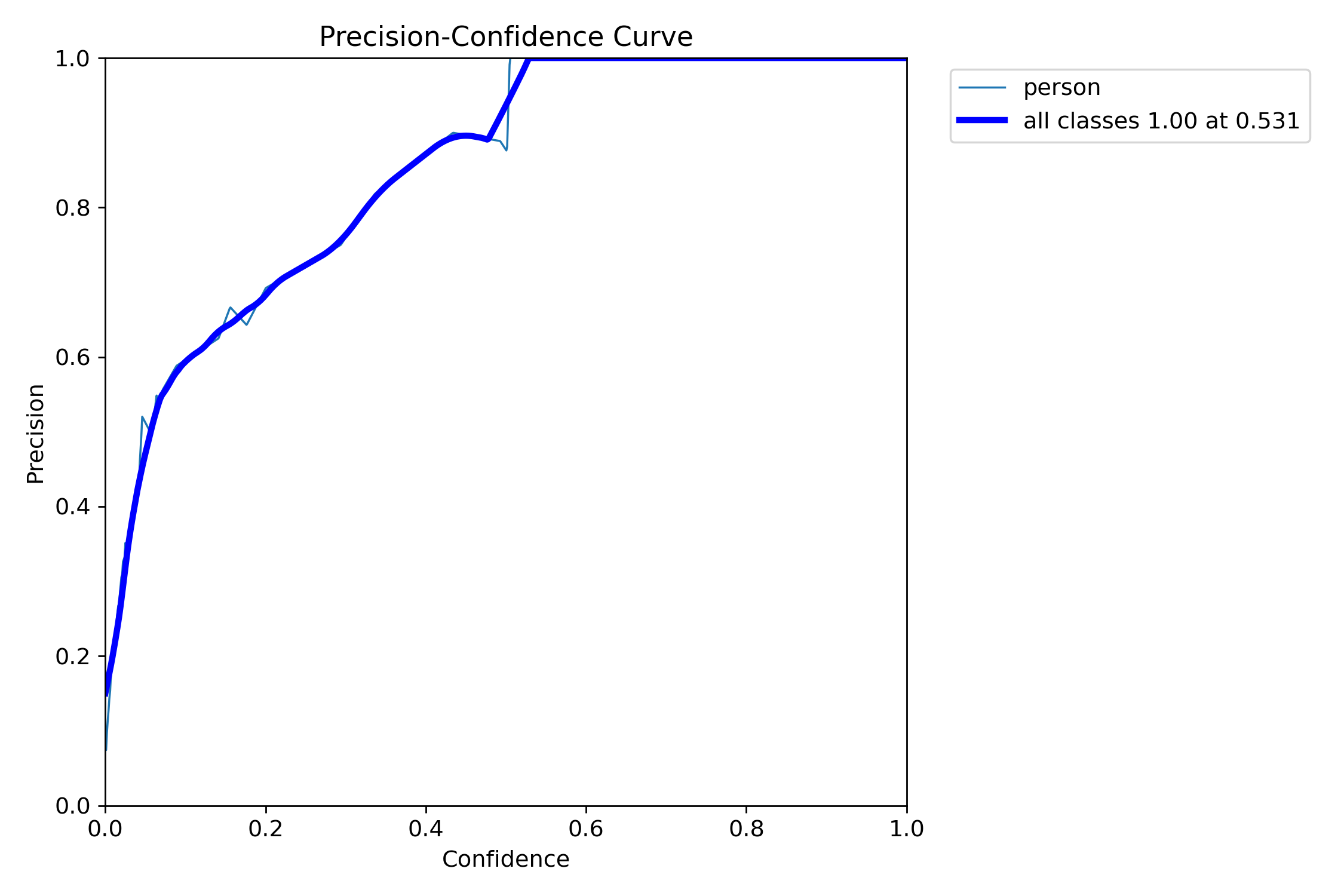

Supplement: S1 File — (ZIP) [file pone.0318578.s002.zip › suooprt information/pose/train32/BoxP_curve.png]

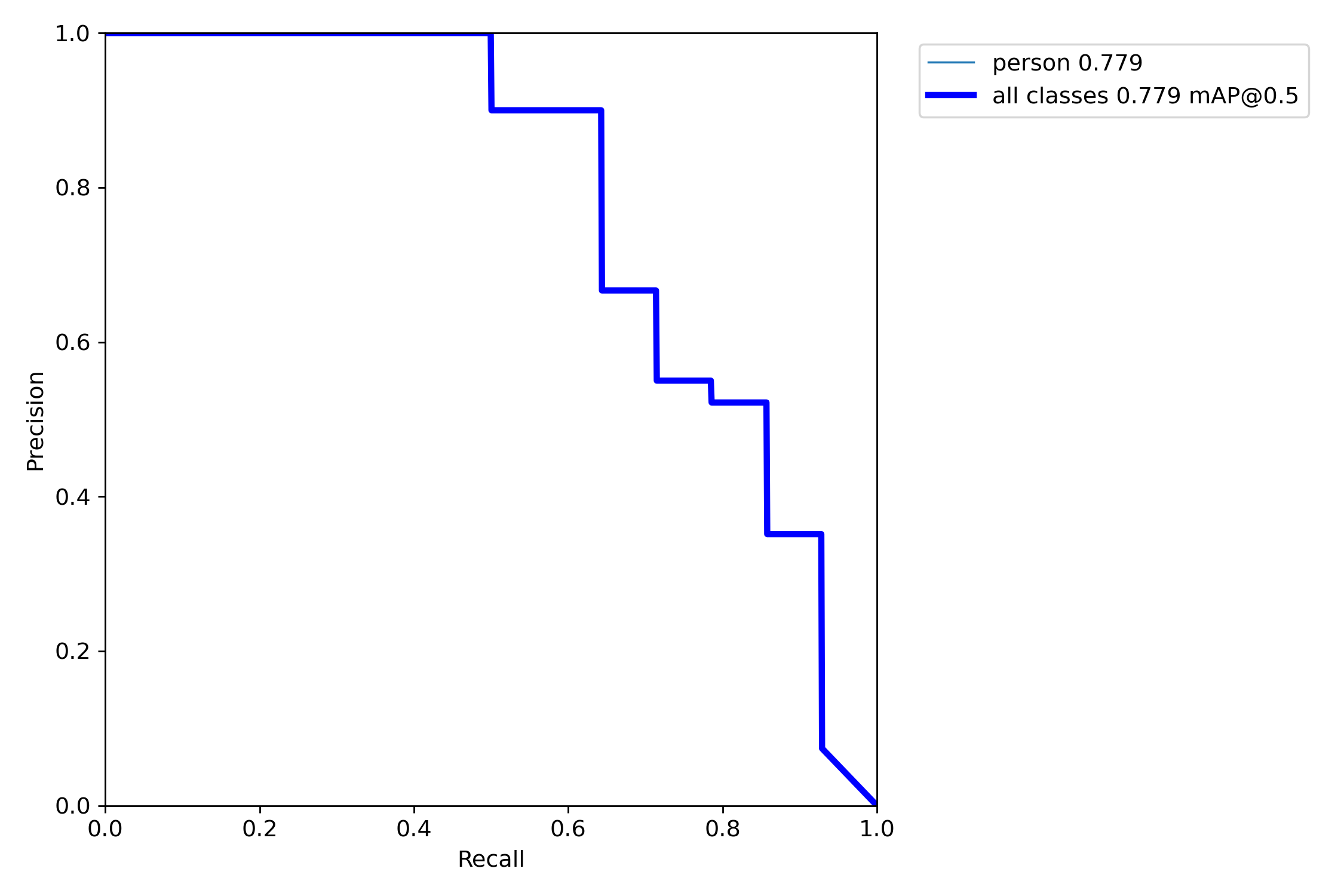

Supplement: S1 File — (ZIP) [file pone.0318578.s002.zip › suooprt information/pose/train32/BoxPR_curve.png]

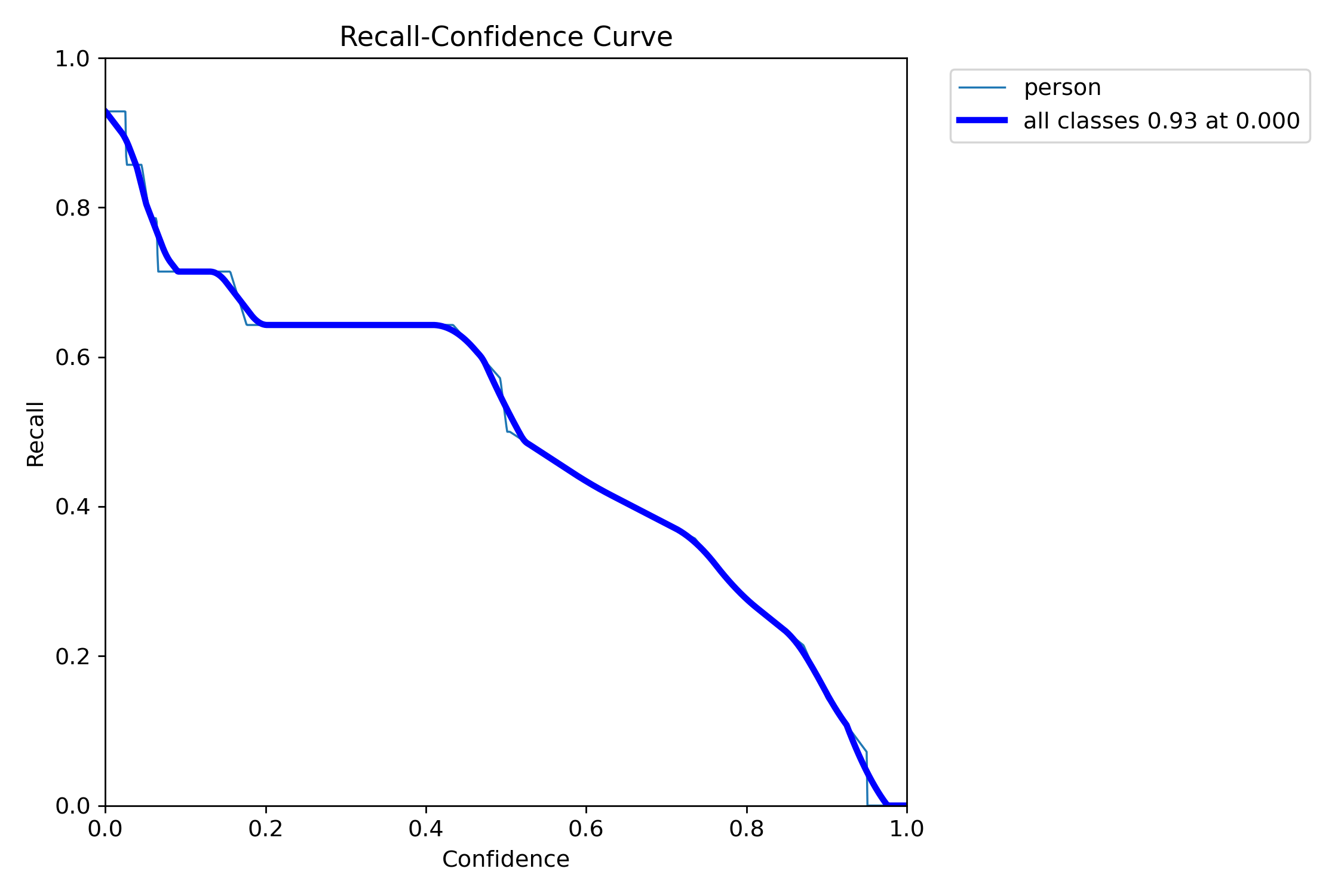

Supplement: S1 File — (ZIP) [file pone.0318578.s002.zip › suooprt information/pose/train32/BoxR_curve.png]

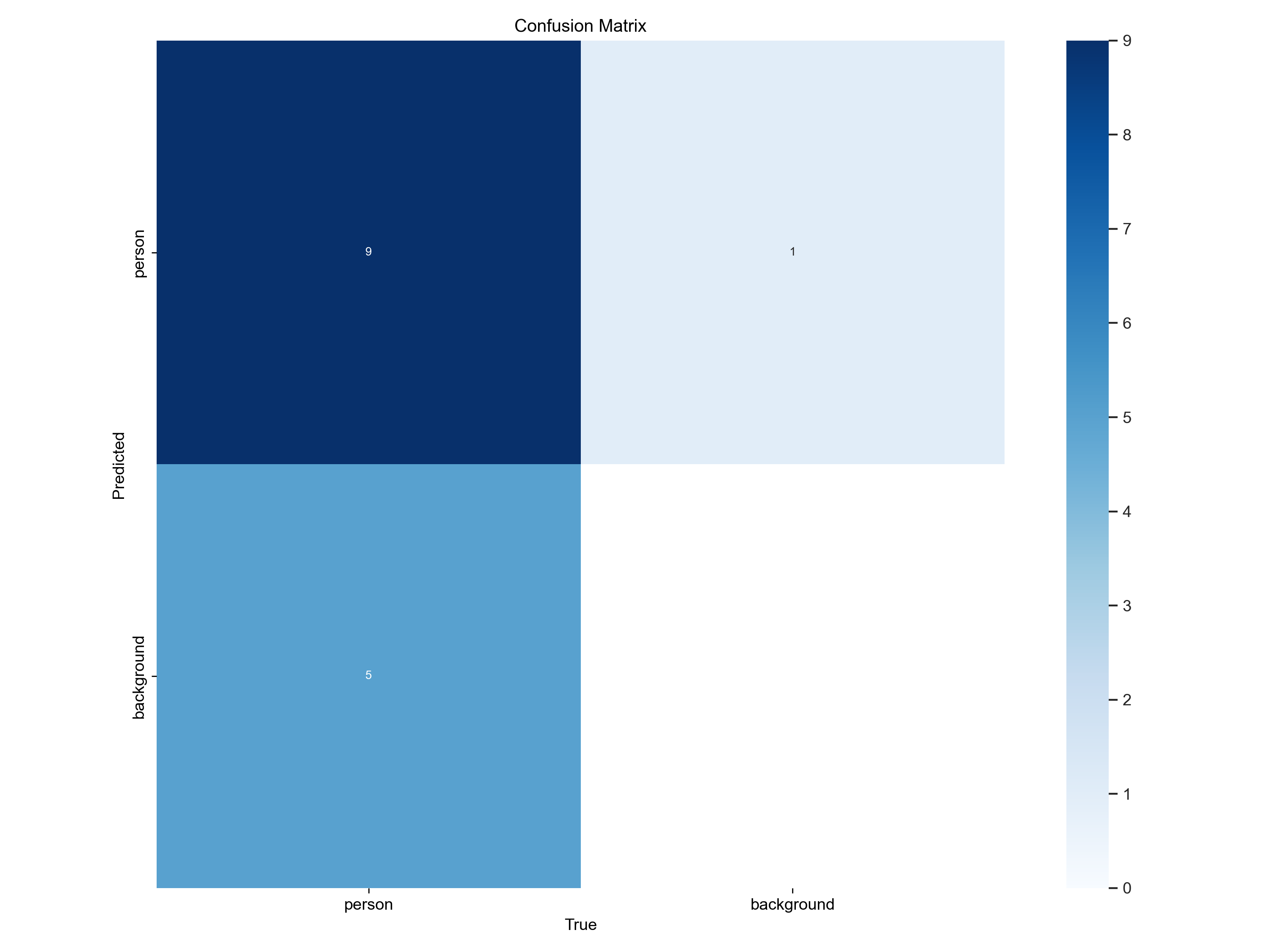

Supplement: S1 File — (ZIP) [file pone.0318578.s002.zip › suooprt information/pose/train32/confusion_matrix.png]

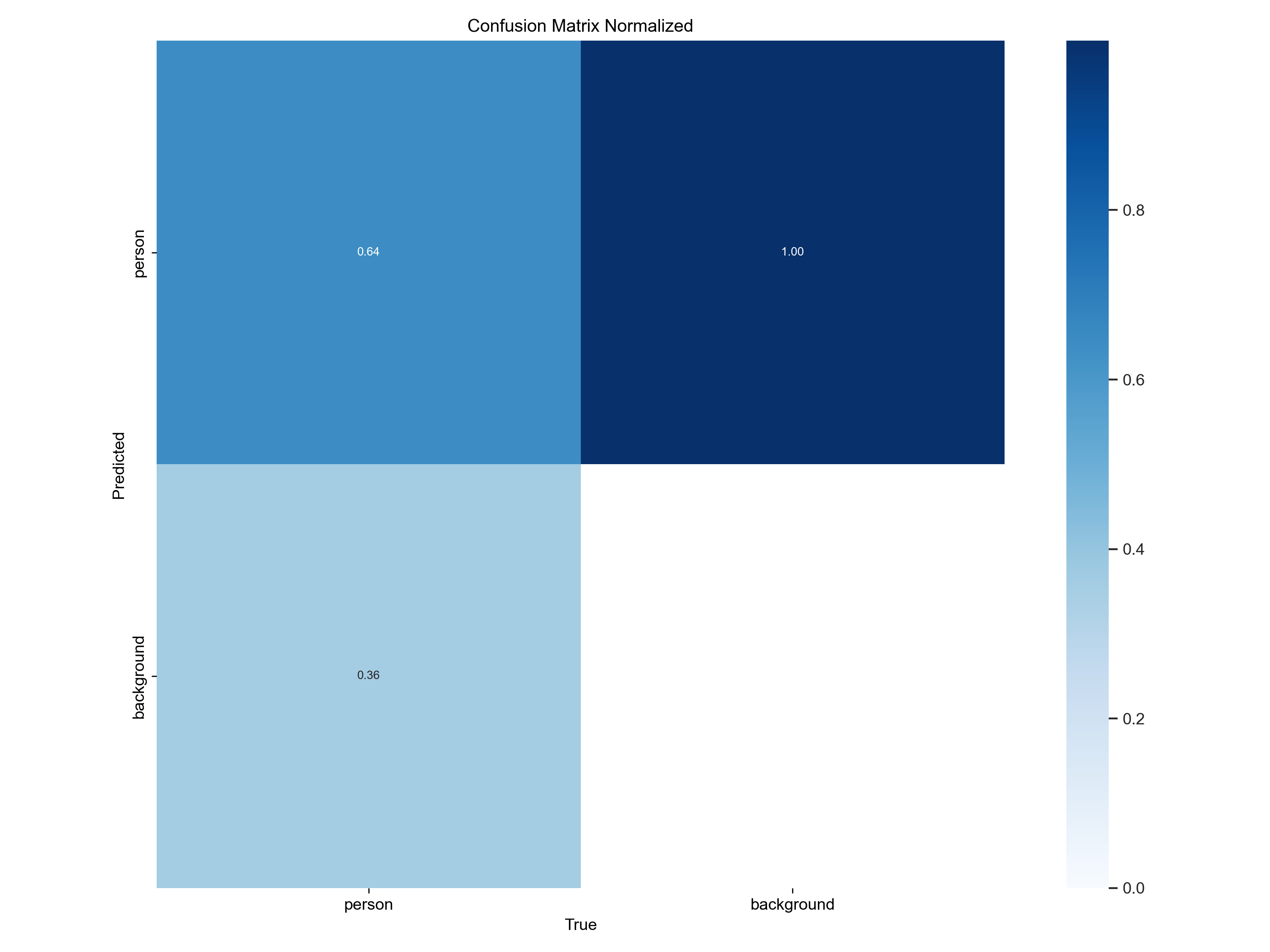

Supplement: S1 File — (ZIP) [file pone.0318578.s002.zip › suooprt information/pose/train32/confusion_matrix_normalized.png]

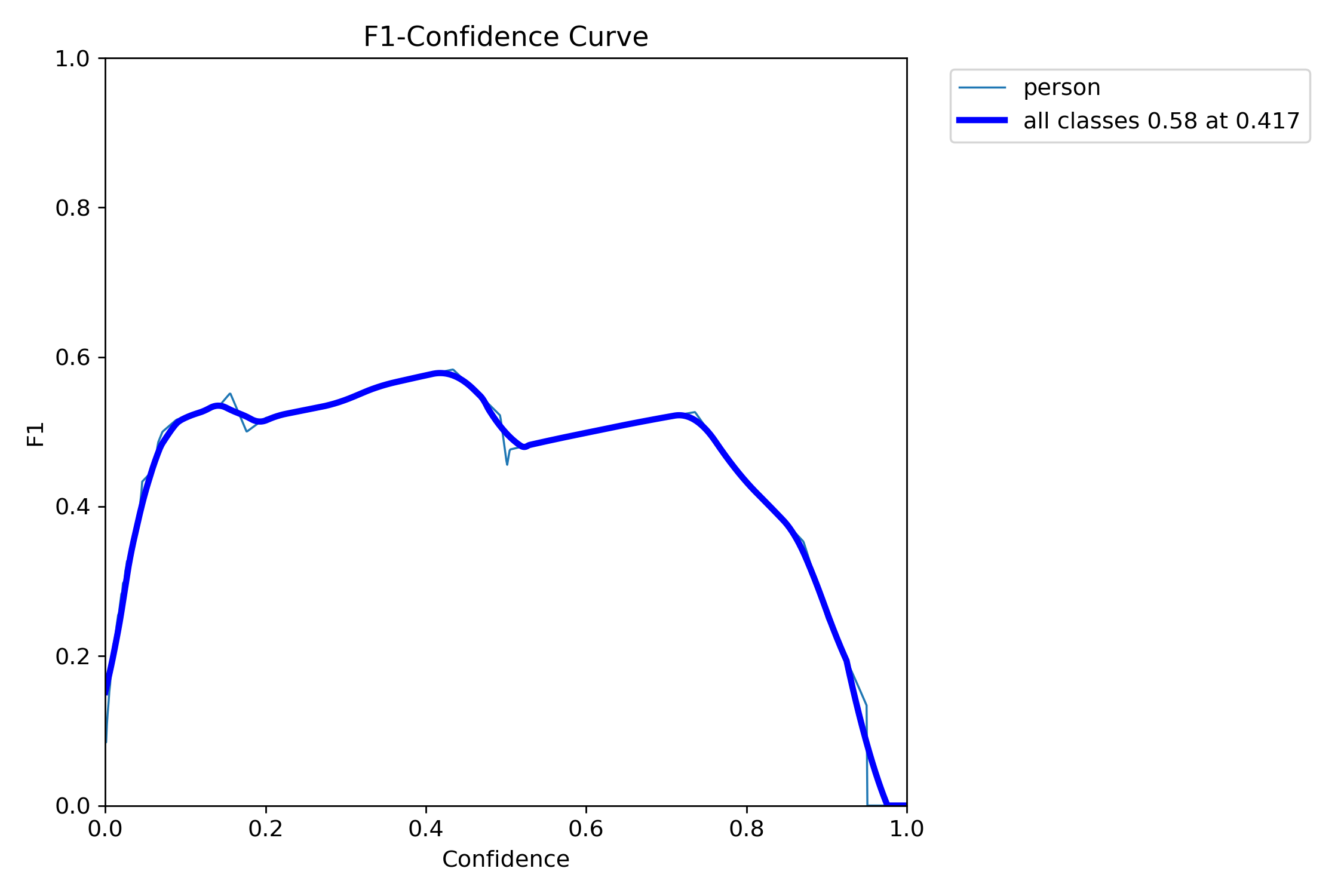

Supplement: S1 File — (ZIP) [file pone.0318578.s002.zip › suooprt information/pose/train32/PoseF1_curve.png]

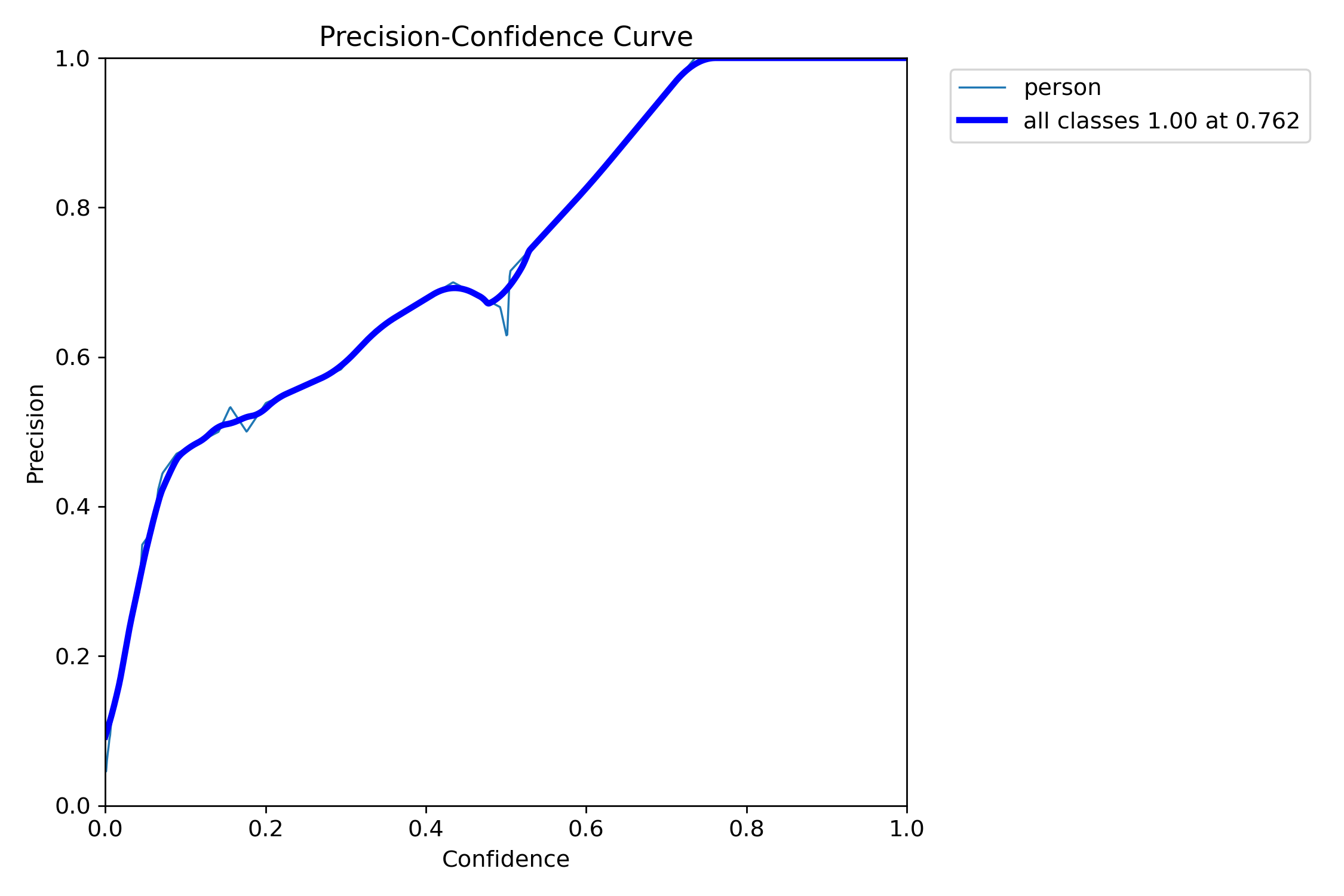

Supplement: S1 File — (ZIP) [file pone.0318578.s002.zip › suooprt information/pose/train32/PoseP_curve.png]

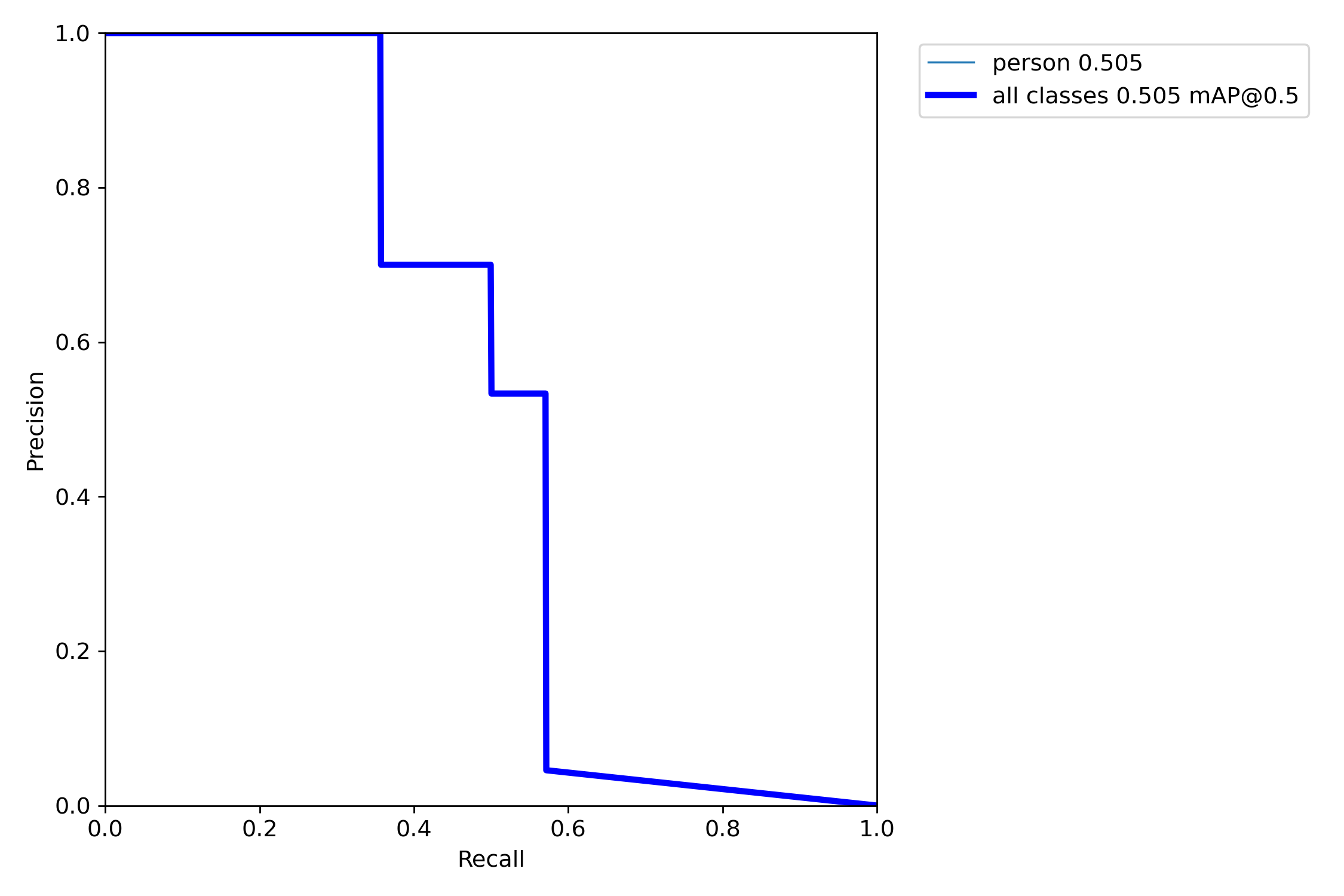

Supplement: S1 File — (ZIP) [file pone.0318578.s002.zip › suooprt information/pose/train32/PosePR_curve.png]

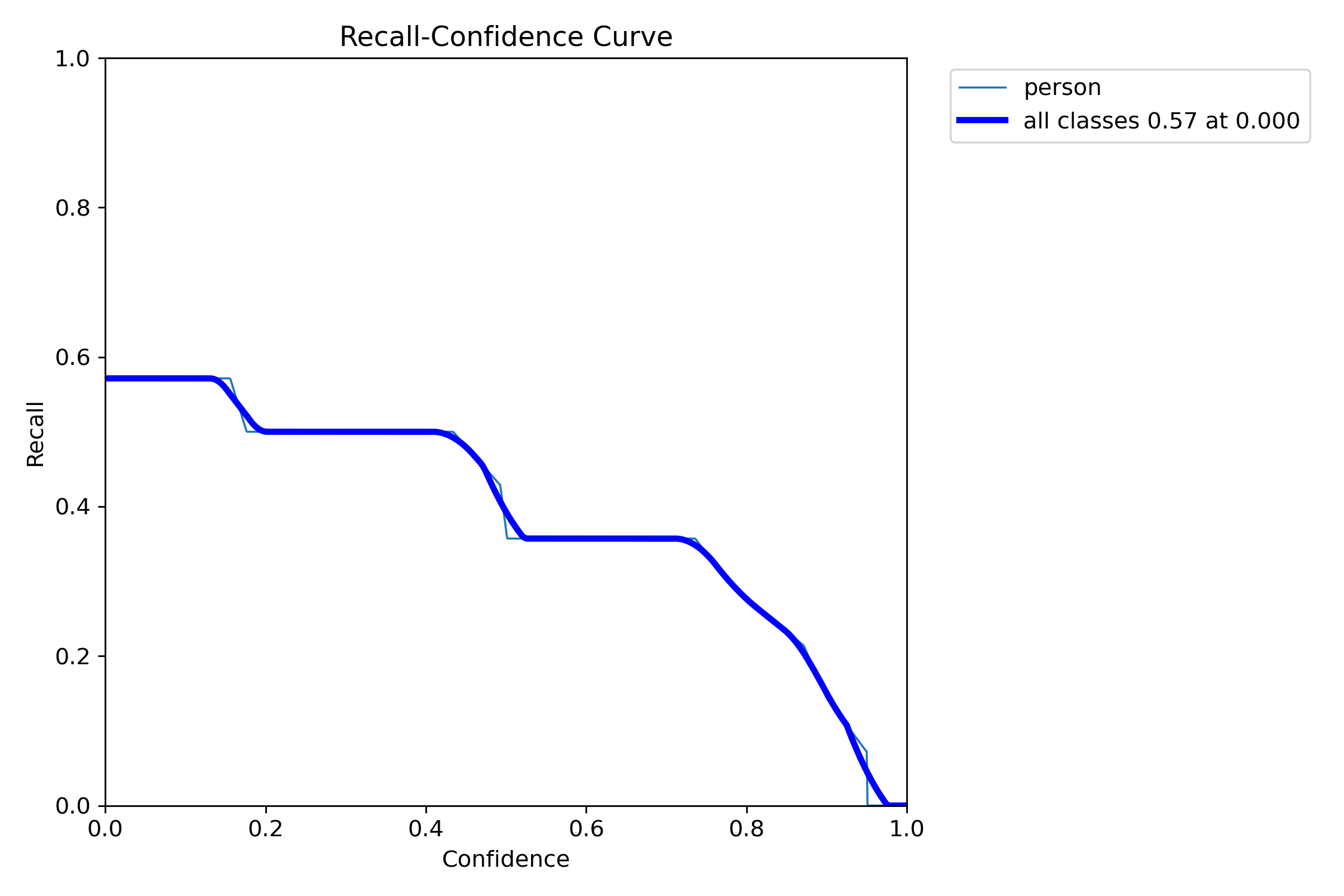

Supplement: S1 File — (ZIP) [file pone.0318578.s002.zip › suooprt information/pose/train32/PoseR_curve.png]

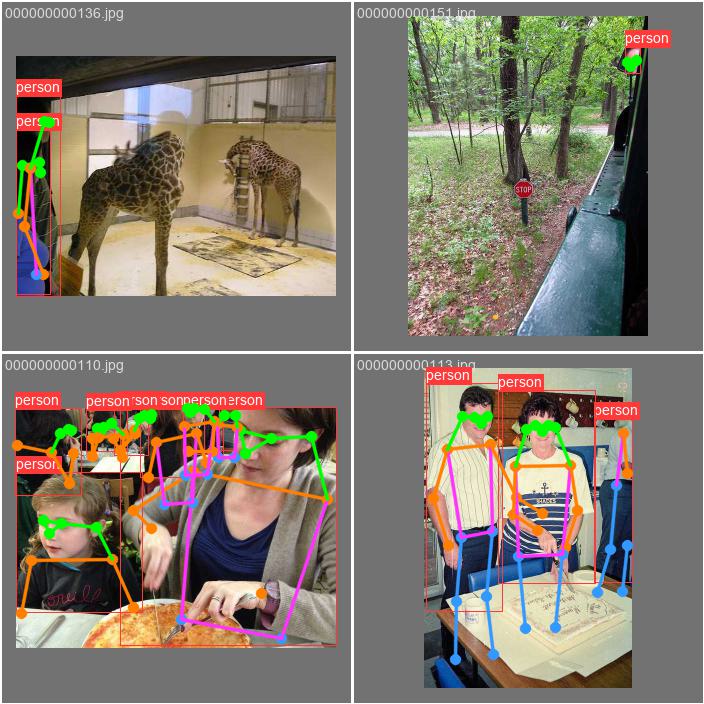

Supplement: S1 File — (ZIP) [file pone.0318578.s002.zip › suooprt information/pose/train32/val_batch0_labels.jpg]

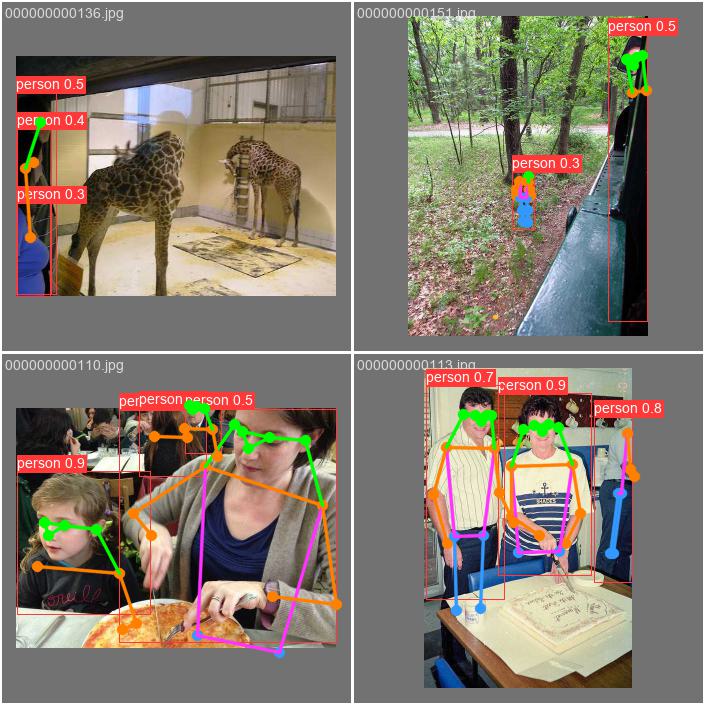

Supplement: S1 File — (ZIP) [file pone.0318578.s002.zip › suooprt information/pose/train32/val_batch0_pred.jpg]

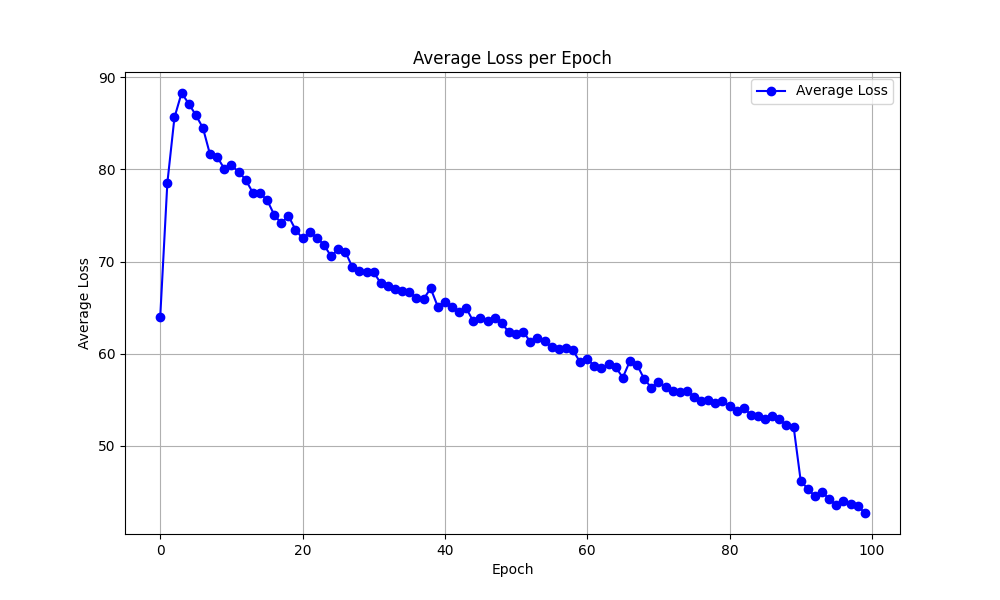

Supplement: S1 File — (ZIP) [file pone.0318578.s002.zip › suooprt information/pose/train33/avg_loss_per_epoch.png]

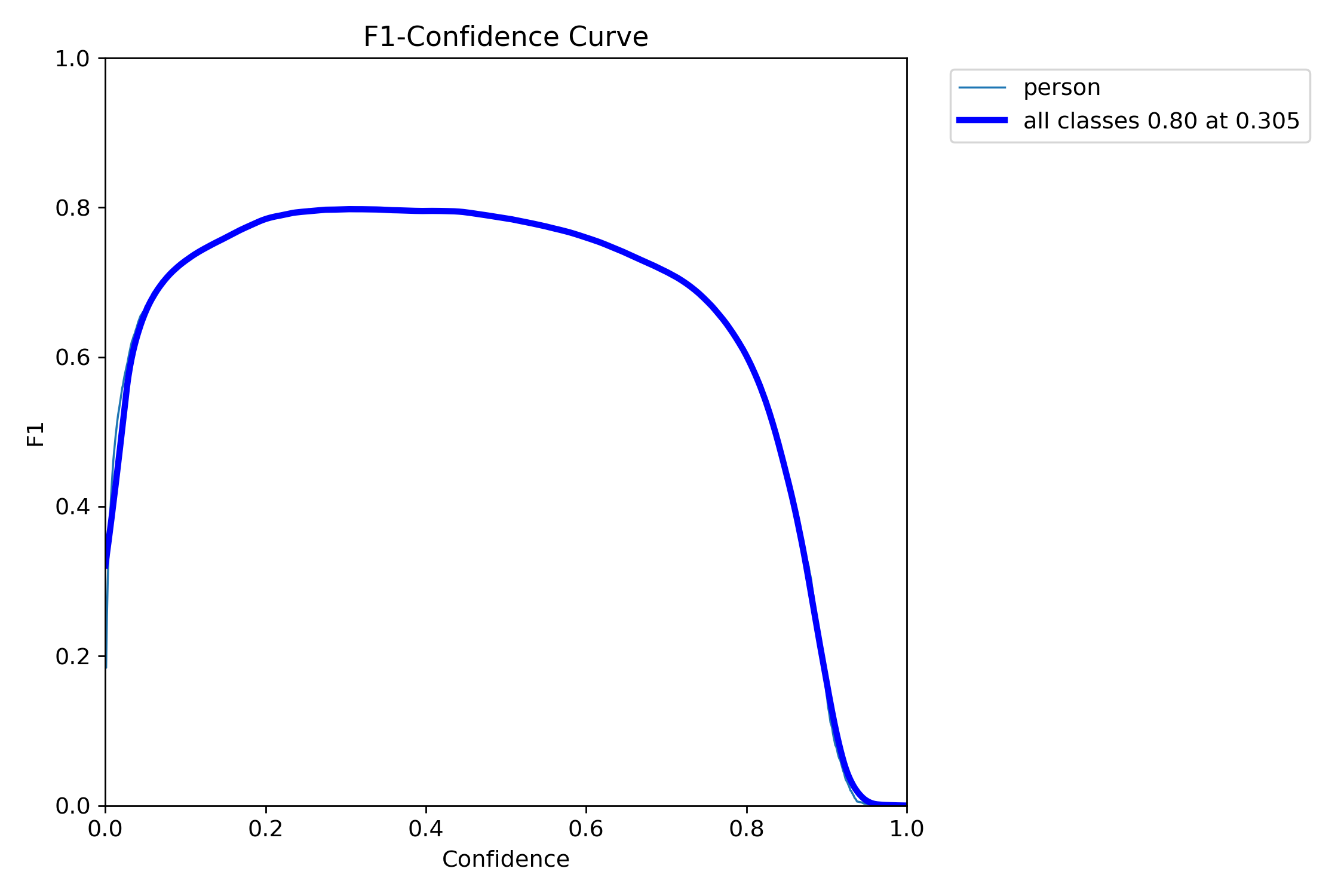

Supplement: S1 File — (ZIP) [file pone.0318578.s002.zip › suooprt information/pose/train33/BoxF1_curve.png]

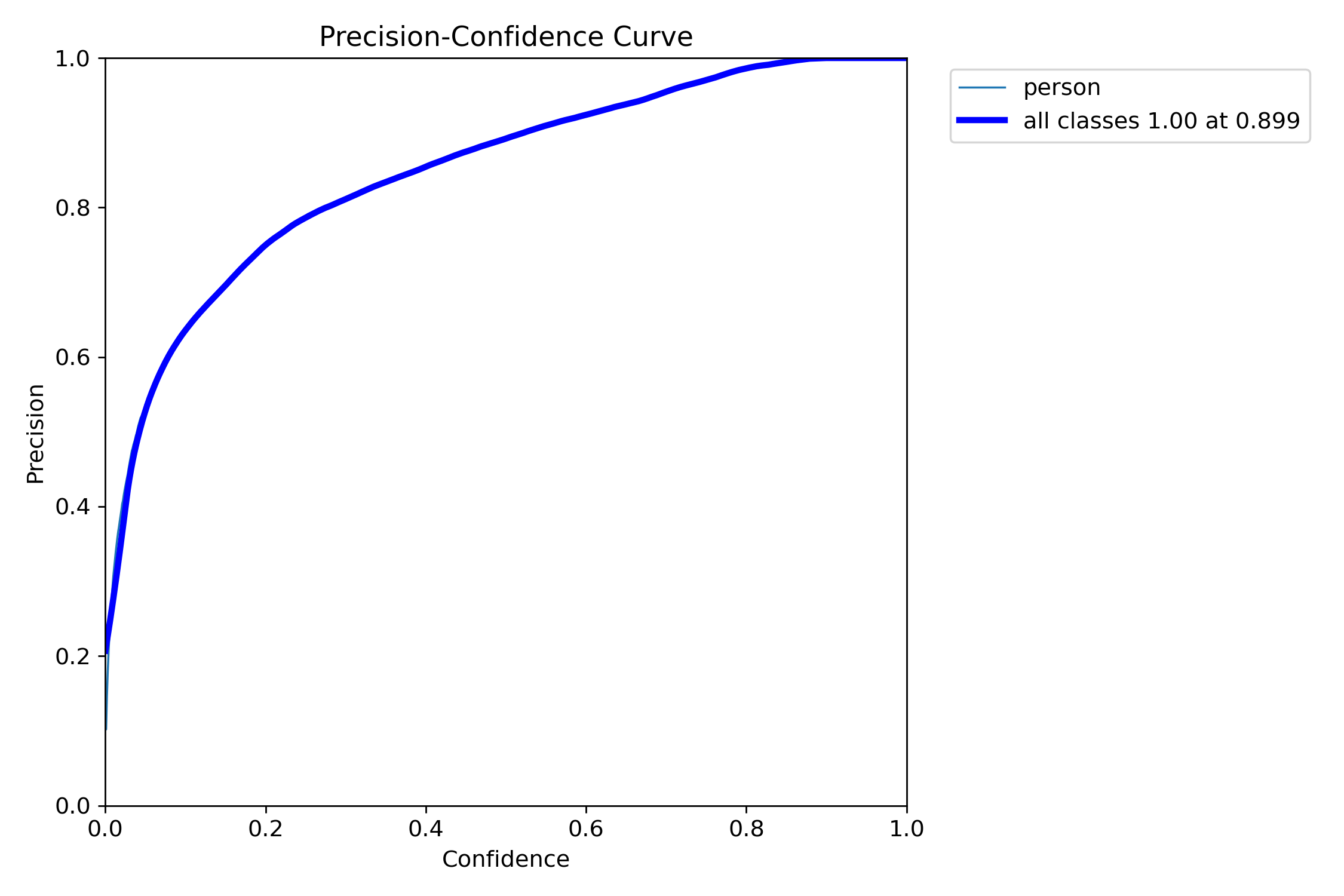

Supplement: S1 File — (ZIP) [file pone.0318578.s002.zip › suooprt information/pose/train33/BoxP_curve.png]

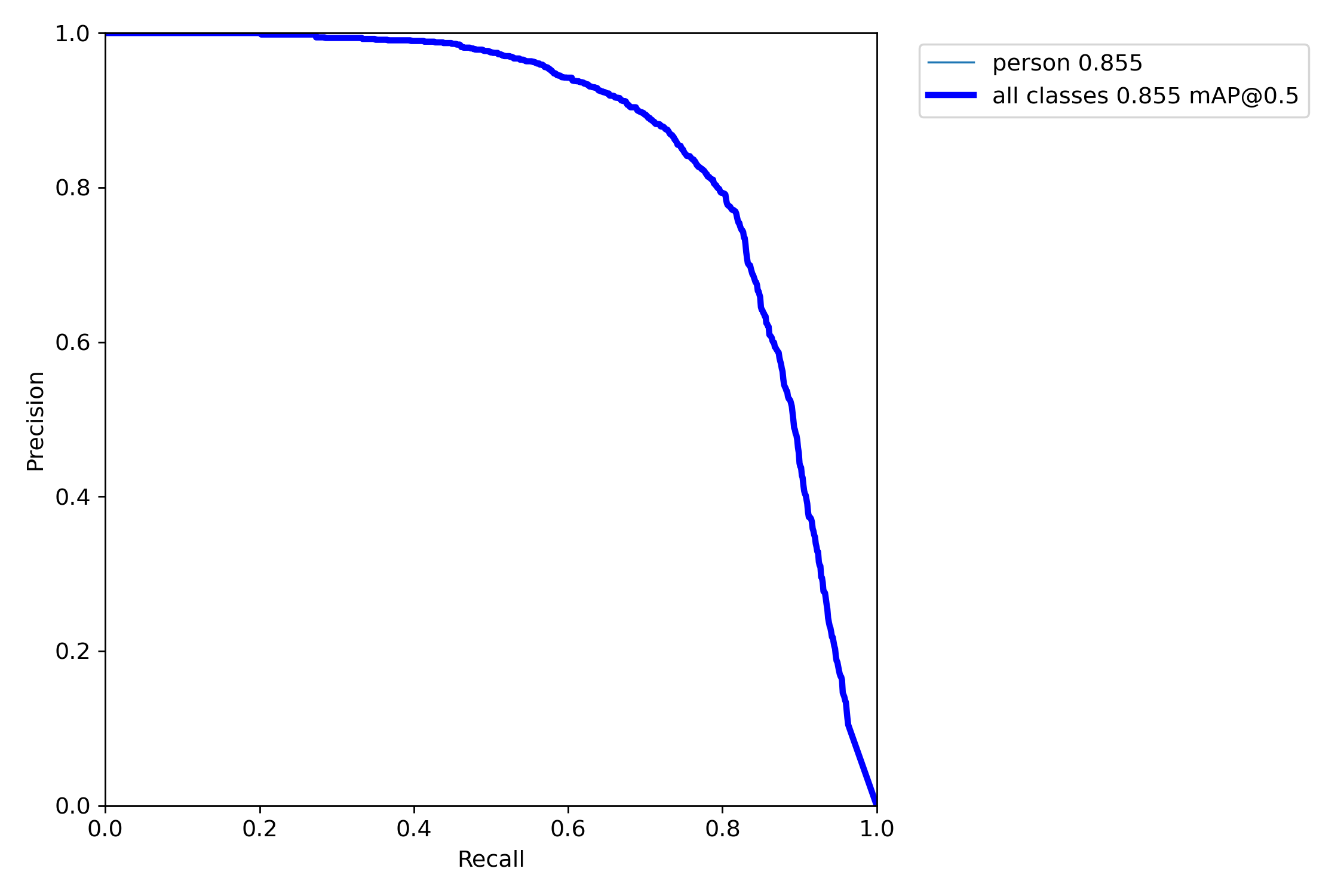

Supplement: S1 File — (ZIP) [file pone.0318578.s002.zip › suooprt information/pose/train33/BoxPR_curve.png]

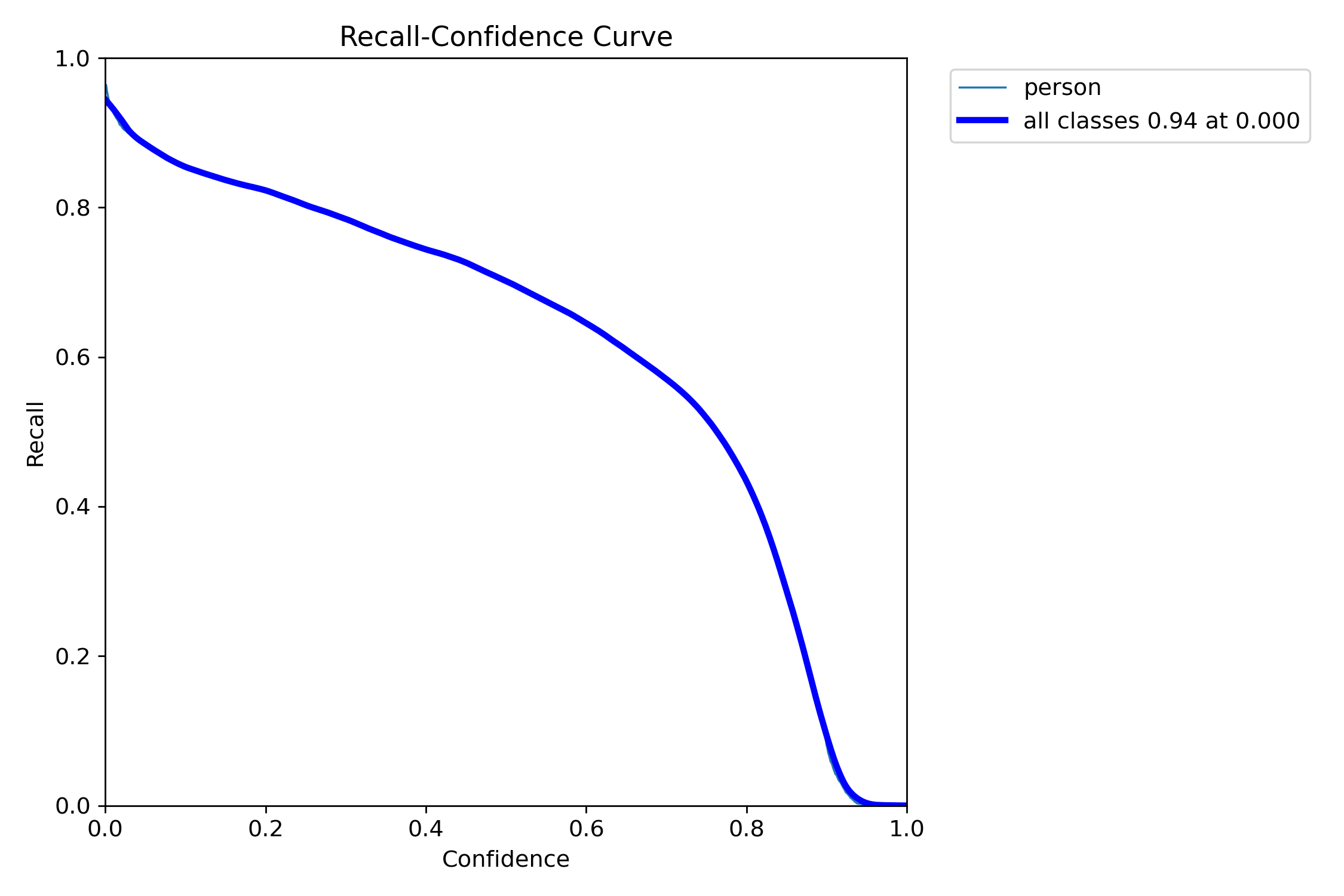

Supplement: S1 File — (ZIP) [file pone.0318578.s002.zip › suooprt information/pose/train33/BoxR_curve.png]

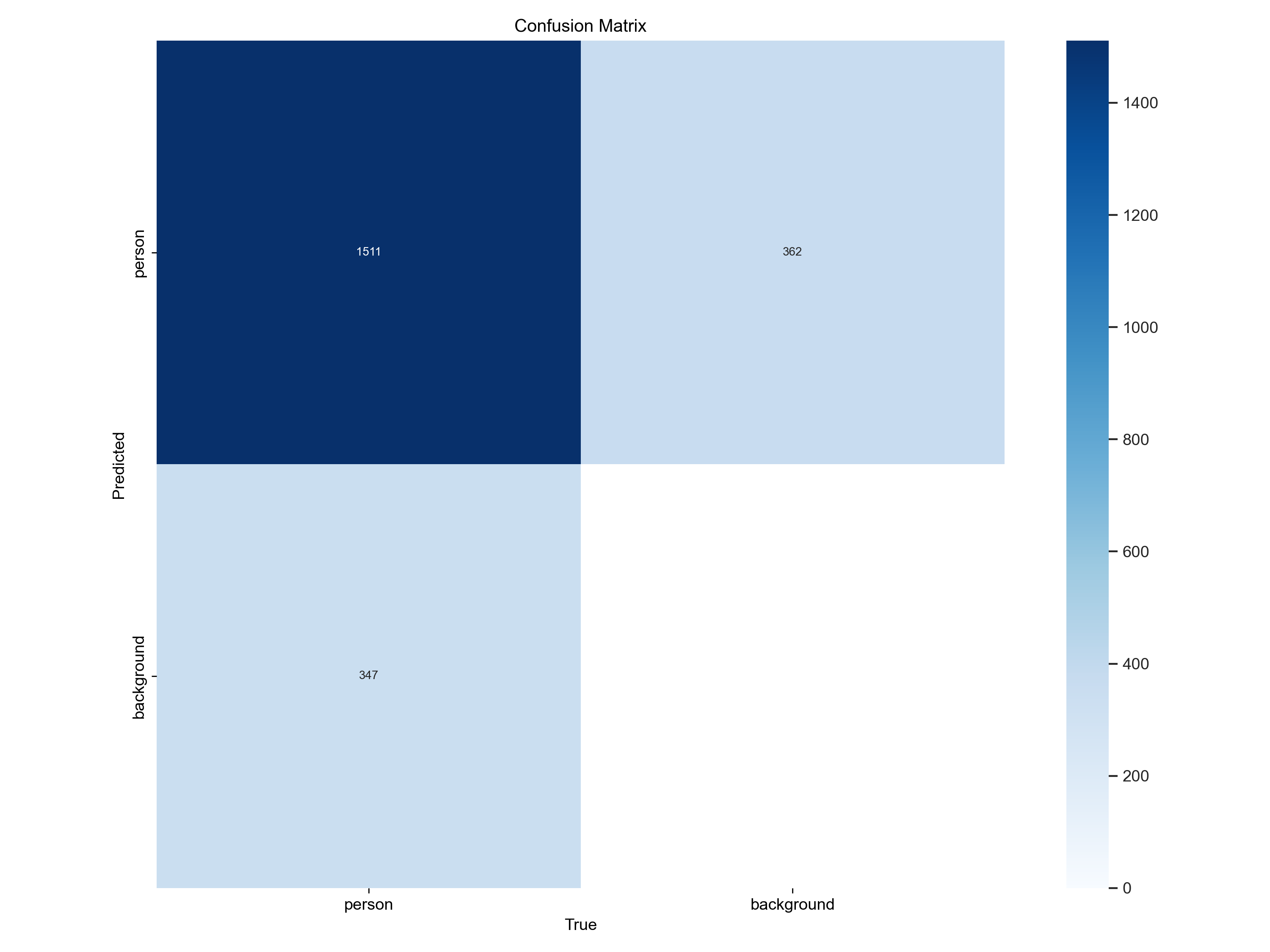

Supplement: S1 File — (ZIP) [file pone.0318578.s002.zip › suooprt information/pose/train33/confusion_matrix.png]

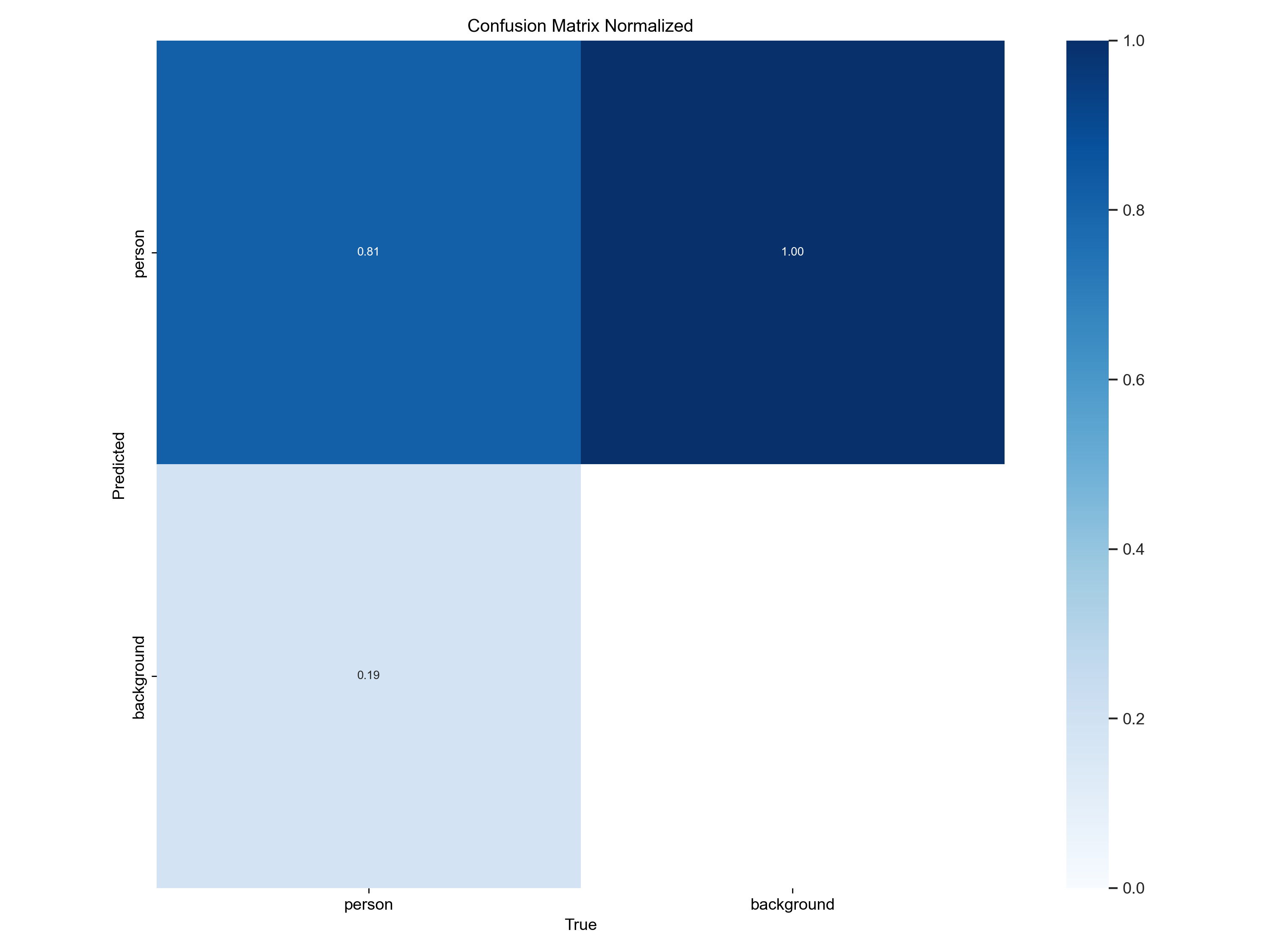

Supplement: S1 File — (ZIP) [file pone.0318578.s002.zip › suooprt information/pose/train33/confusion_matrix_normalized.png]

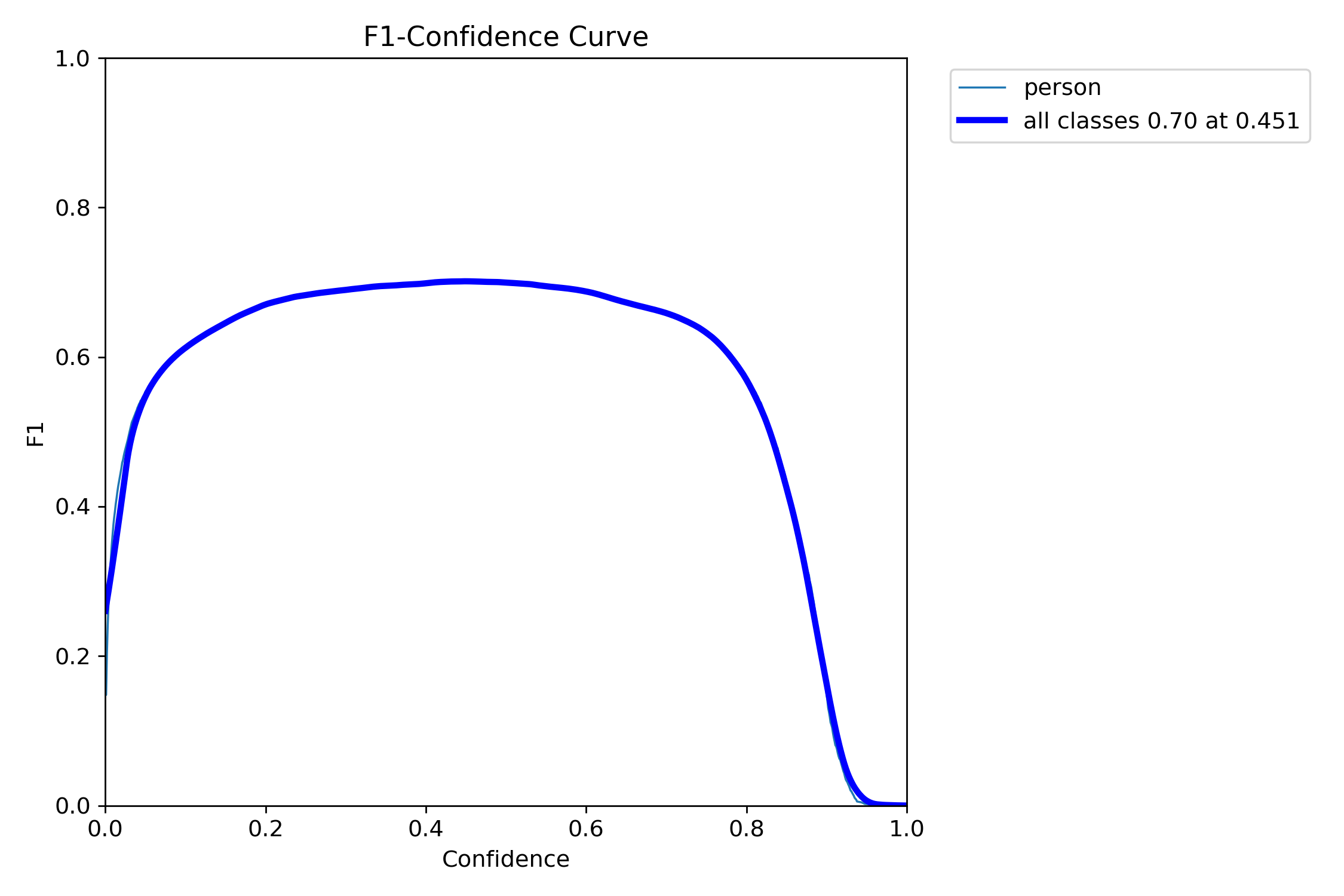

Supplement: S1 File — (ZIP) [file pone.0318578.s002.zip › suooprt information/pose/train33/PoseF1_curve.png]

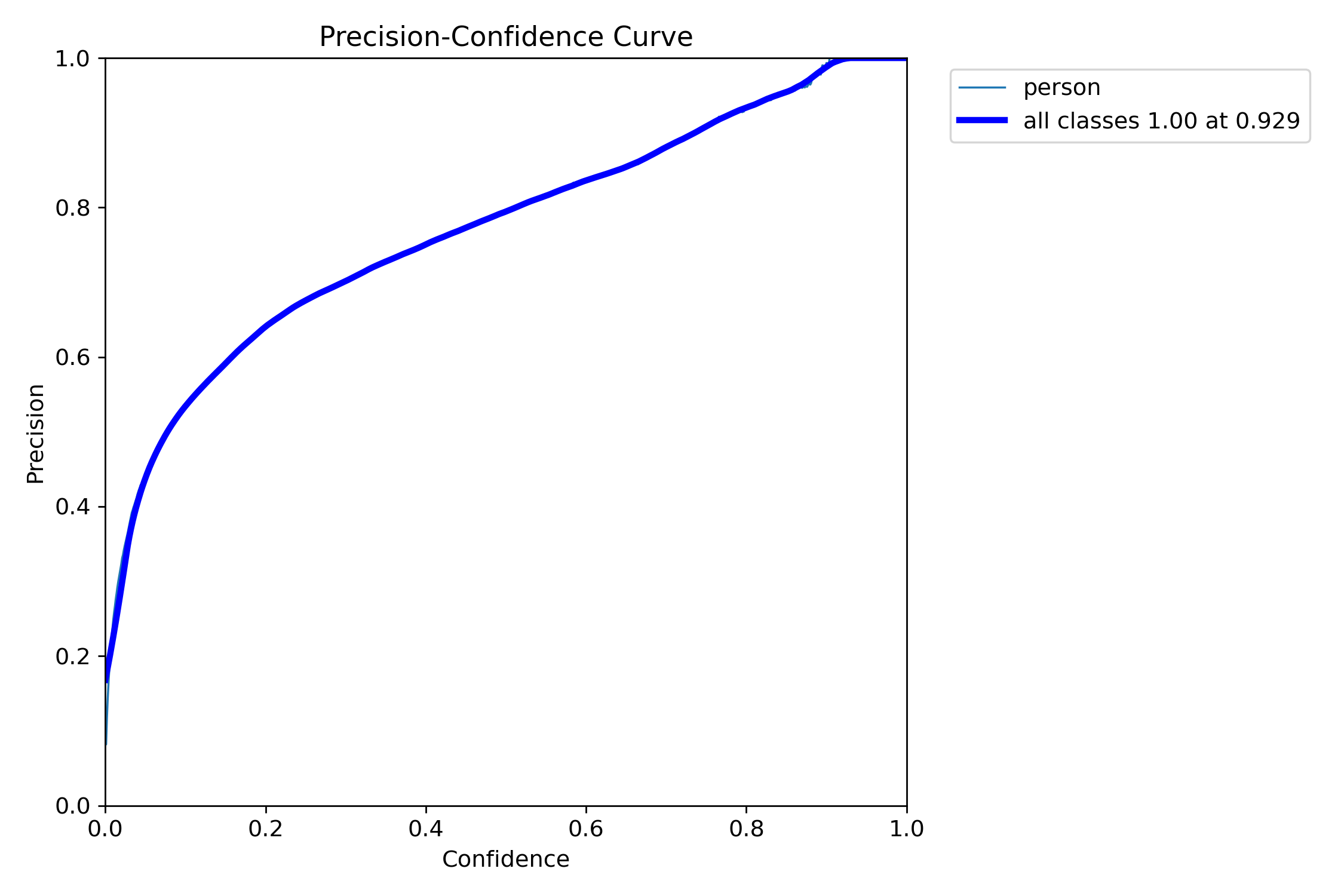

Supplement: S1 File — (ZIP) [file pone.0318578.s002.zip › suooprt information/pose/train33/PoseP_curve.png]

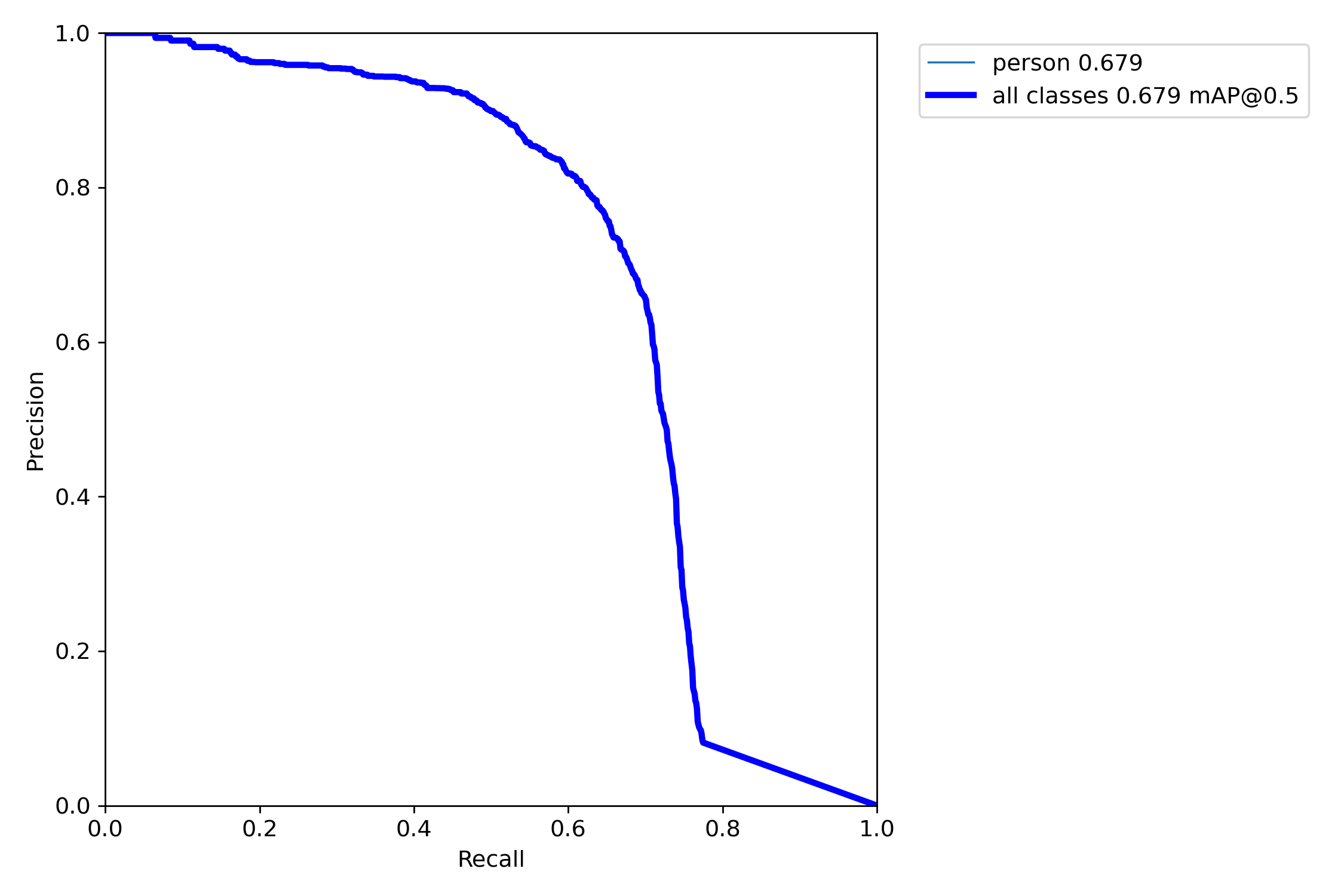

Supplement: S1 File — (ZIP) [file pone.0318578.s002.zip › suooprt information/pose/train33/PosePR_curve.png]

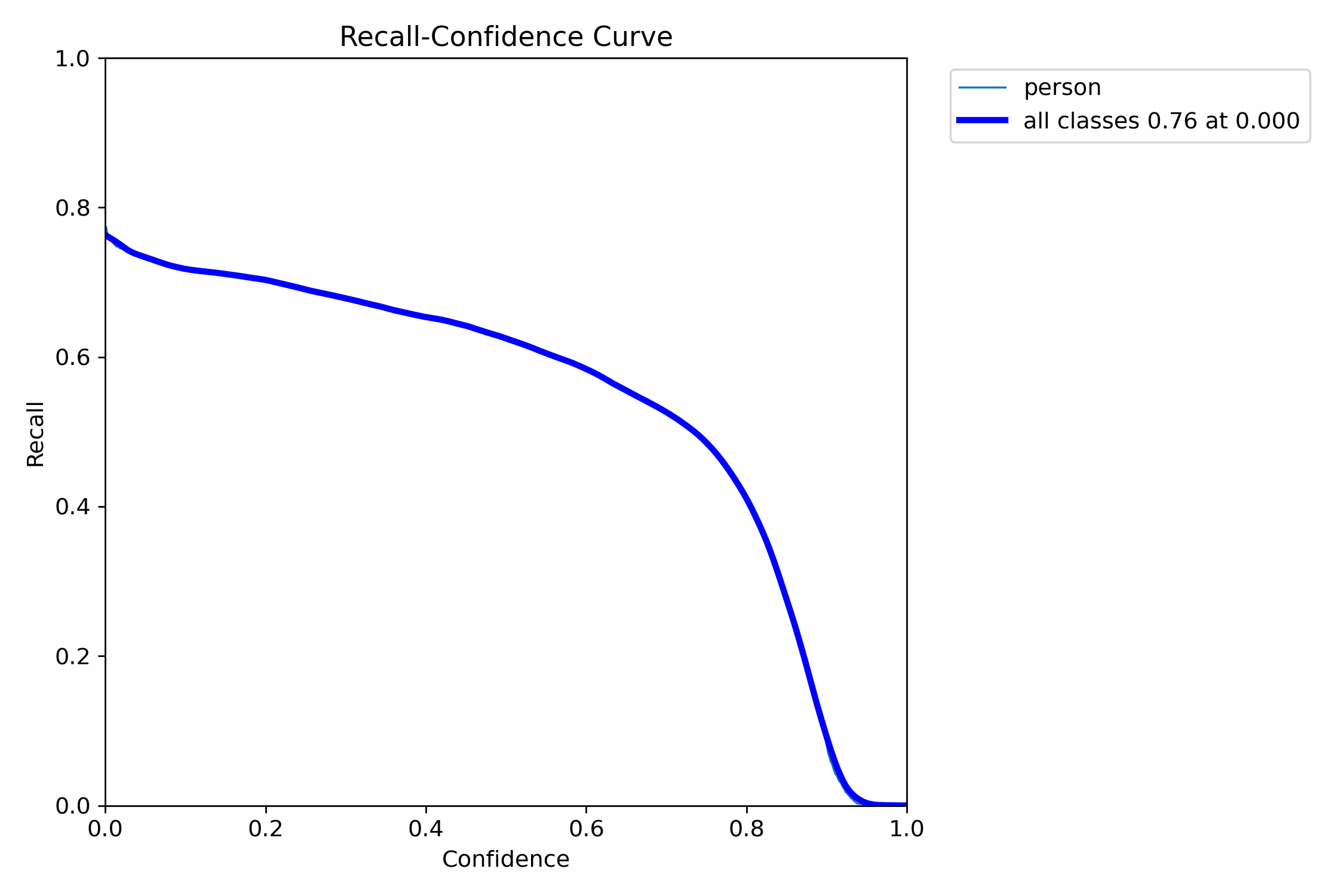

Supplement: S1 File — (ZIP) [file pone.0318578.s002.zip › suooprt information/pose/train33/PoseR_curve.png]

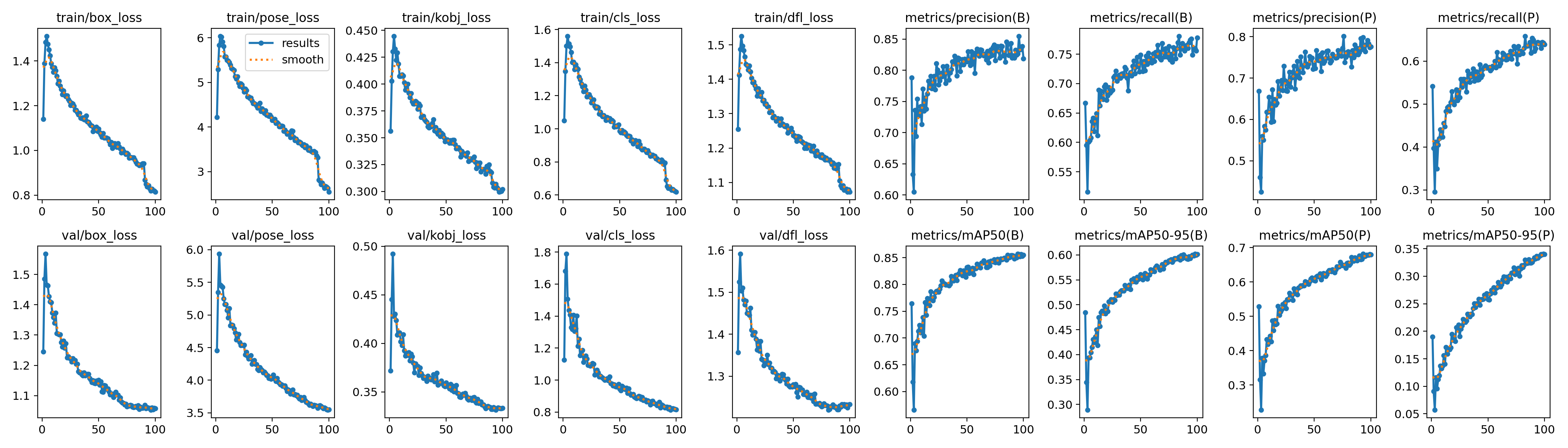

Supplement: S1 File — (ZIP) [file pone.0318578.s002.zip › suooprt information/pose/train33/results.png]

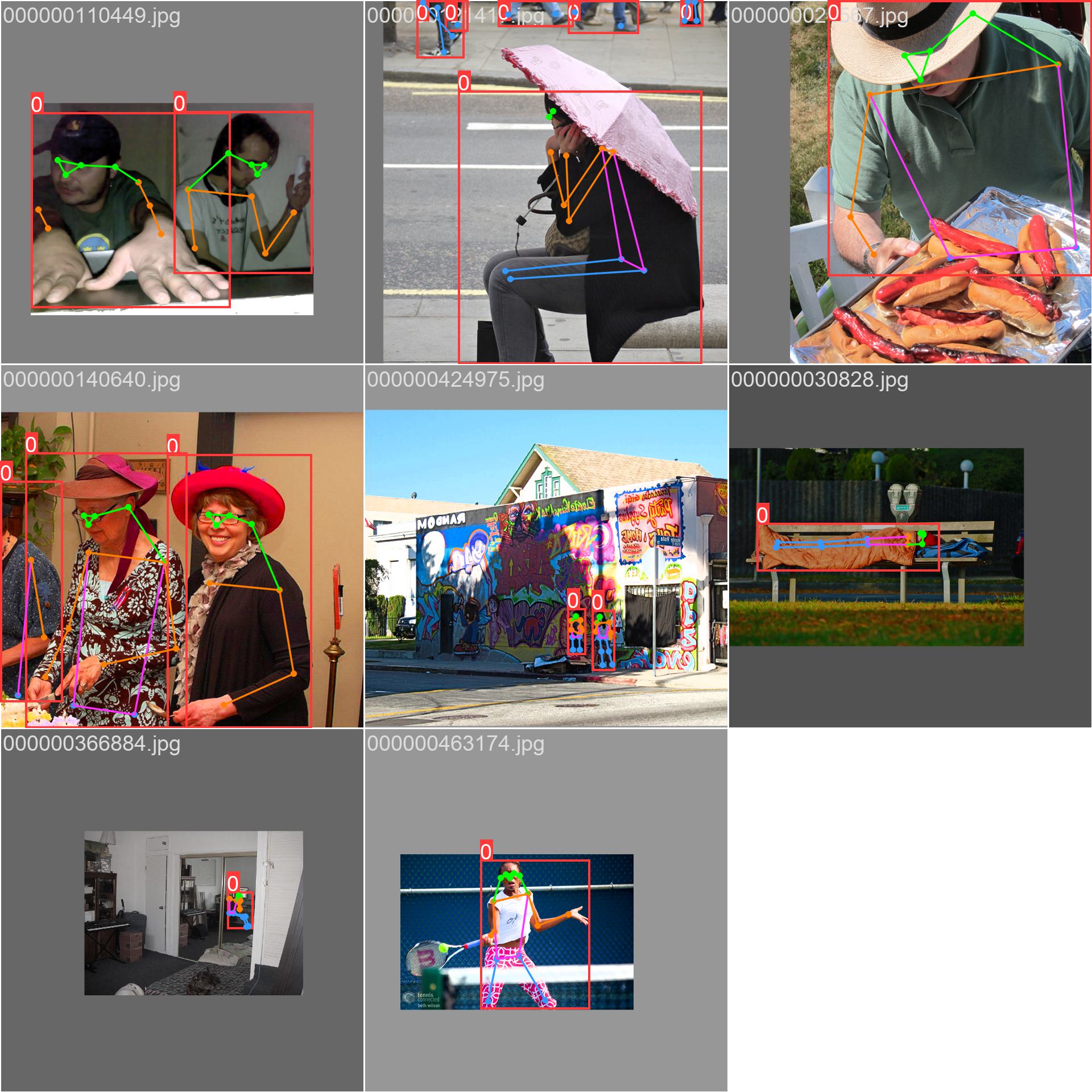

Supplement: S1 File — (ZIP) [file pone.0318578.s002.zip › suooprt information/pose/train33/train_batch18540.jpg]

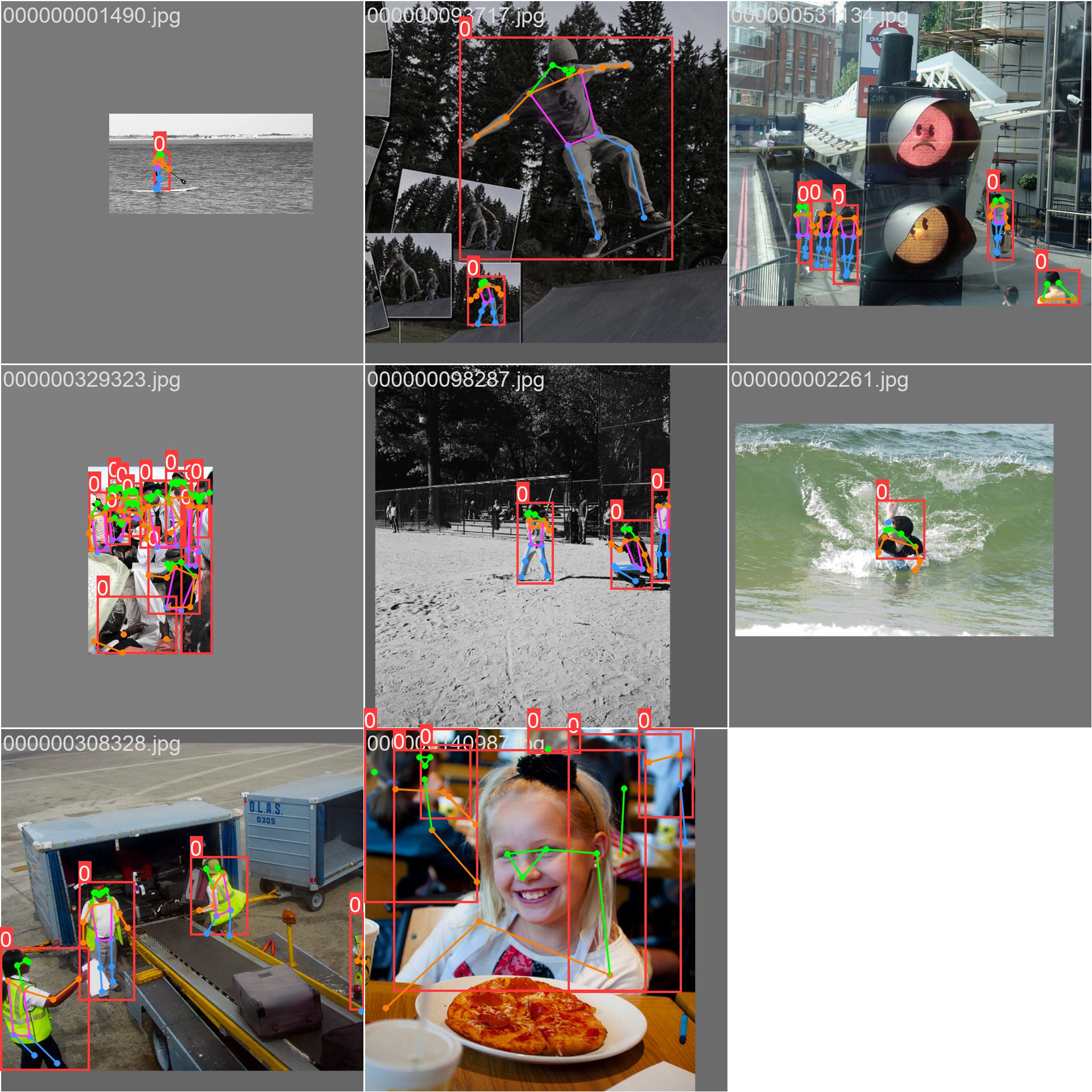

Supplement: S1 File — (ZIP) [file pone.0318578.s002.zip › suooprt information/pose/train33/train_batch18541.jpg]

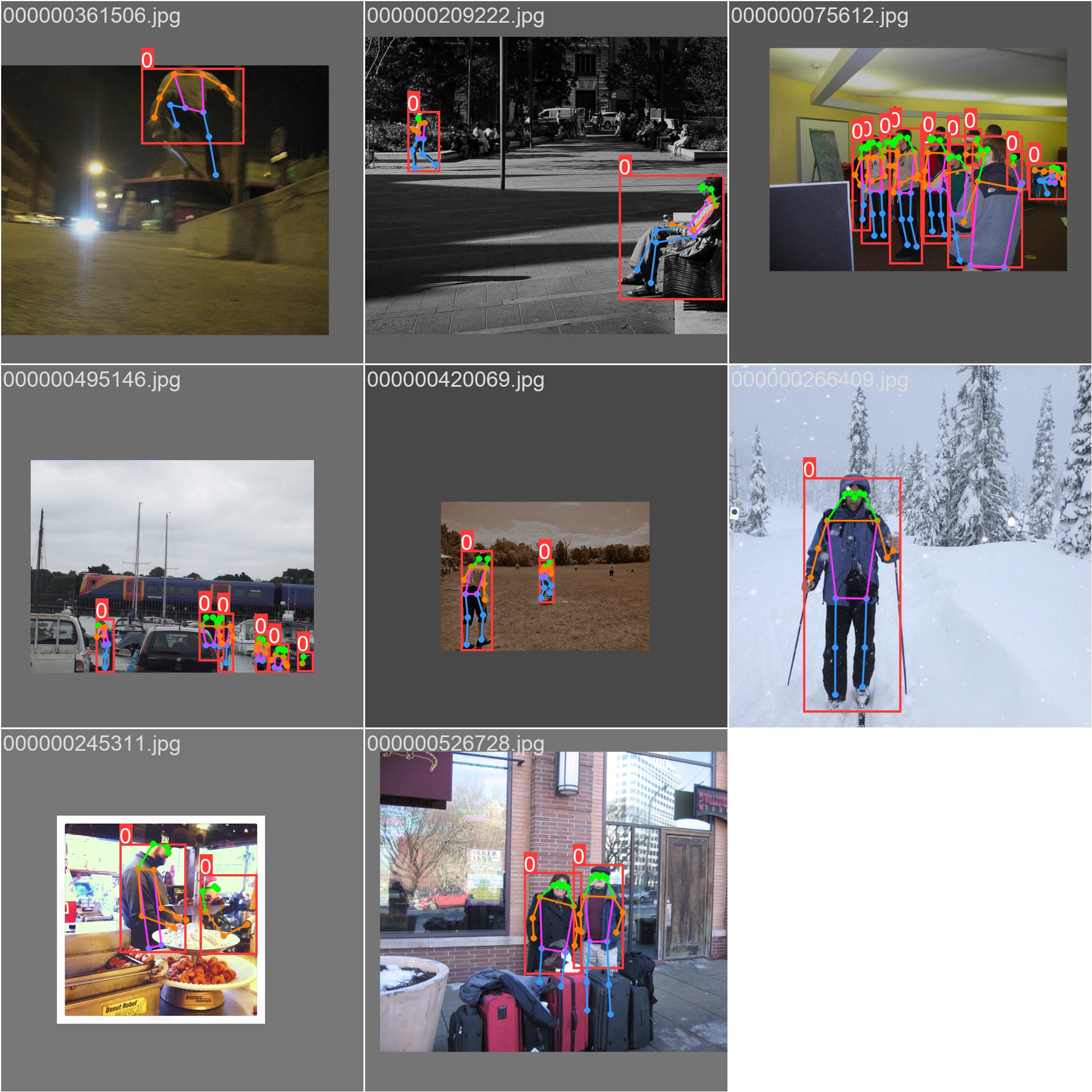

Supplement: S1 File — (ZIP) [file pone.0318578.s002.zip › suooprt information/pose/train33/train_batch18542.jpg]

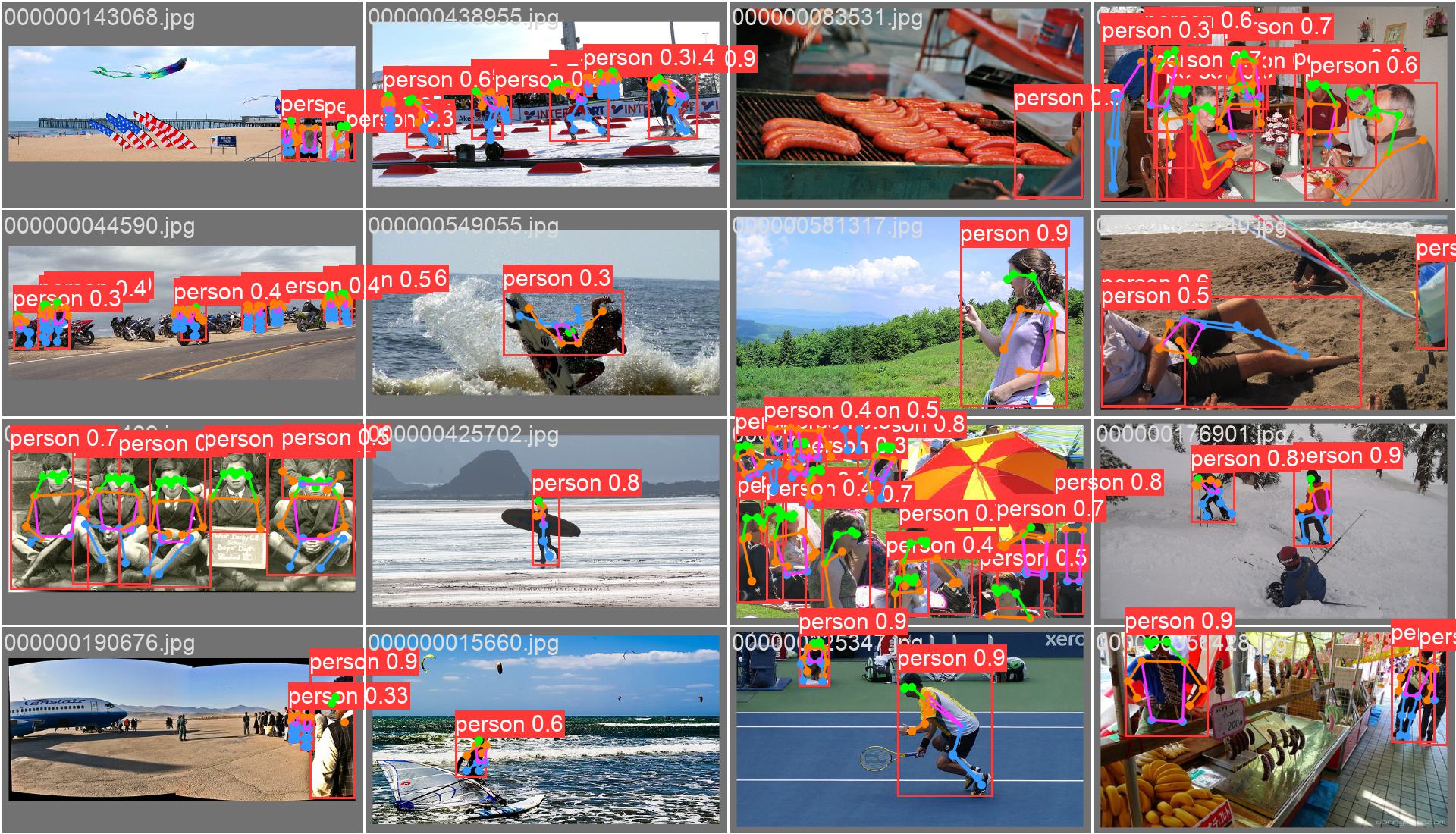

Supplement: S1 File — (ZIP) [file pone.0318578.s002.zip › suooprt information/pose/train33/val_batch0_pred.jpg]

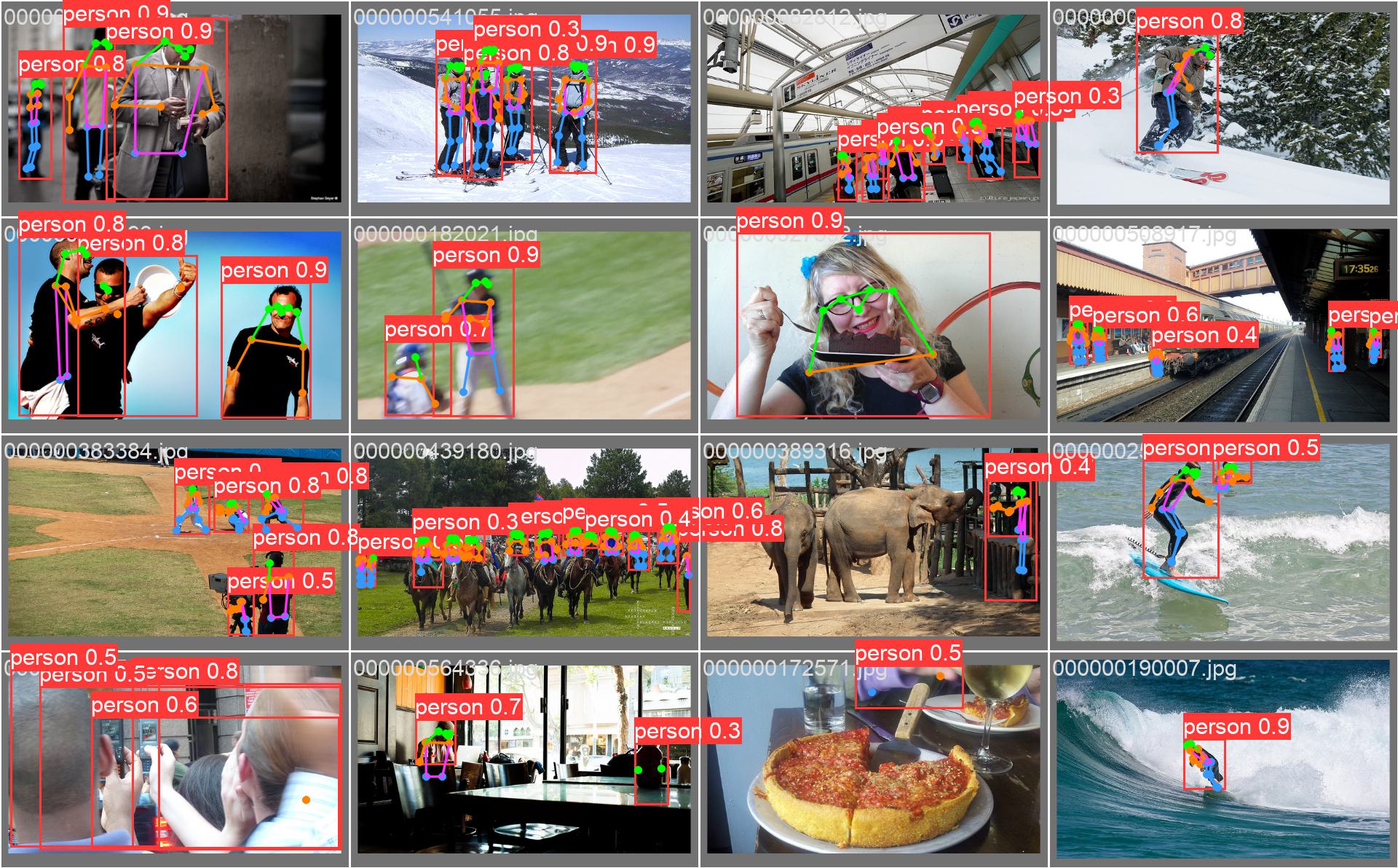

Supplement: S1 File — (ZIP) [file pone.0318578.s002.zip › suooprt information/pose/train33/val_batch1_pred.jpg]

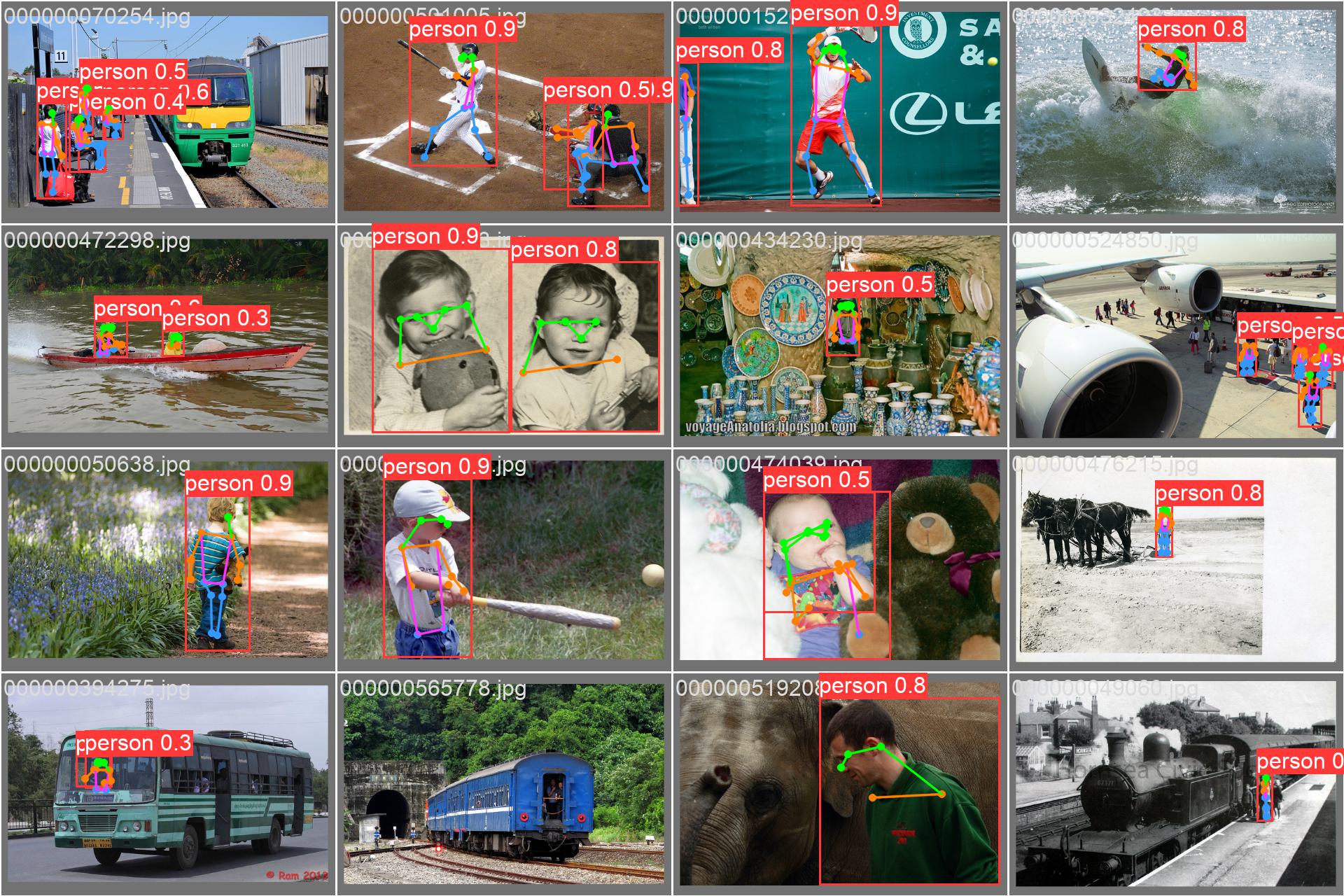

Supplement: S1 File — (ZIP) [file pone.0318578.s002.zip › suooprt information/pose/train33/val_batch2_pred.jpg]

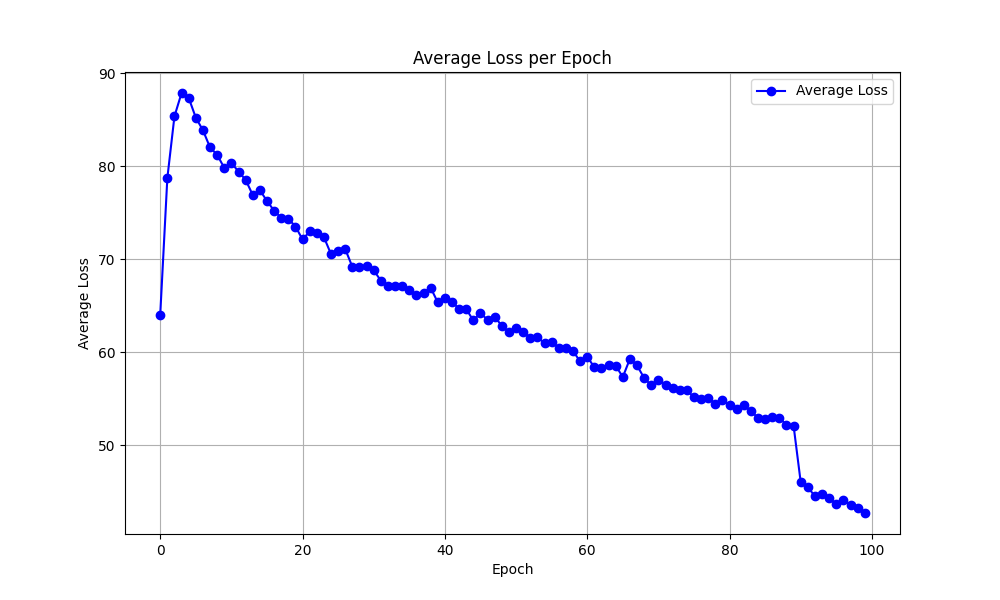

Supplement: S1 File — (ZIP) [file pone.0318578.s002.zip › suooprt information/pose/train34/avg_loss_per_epoch.png]

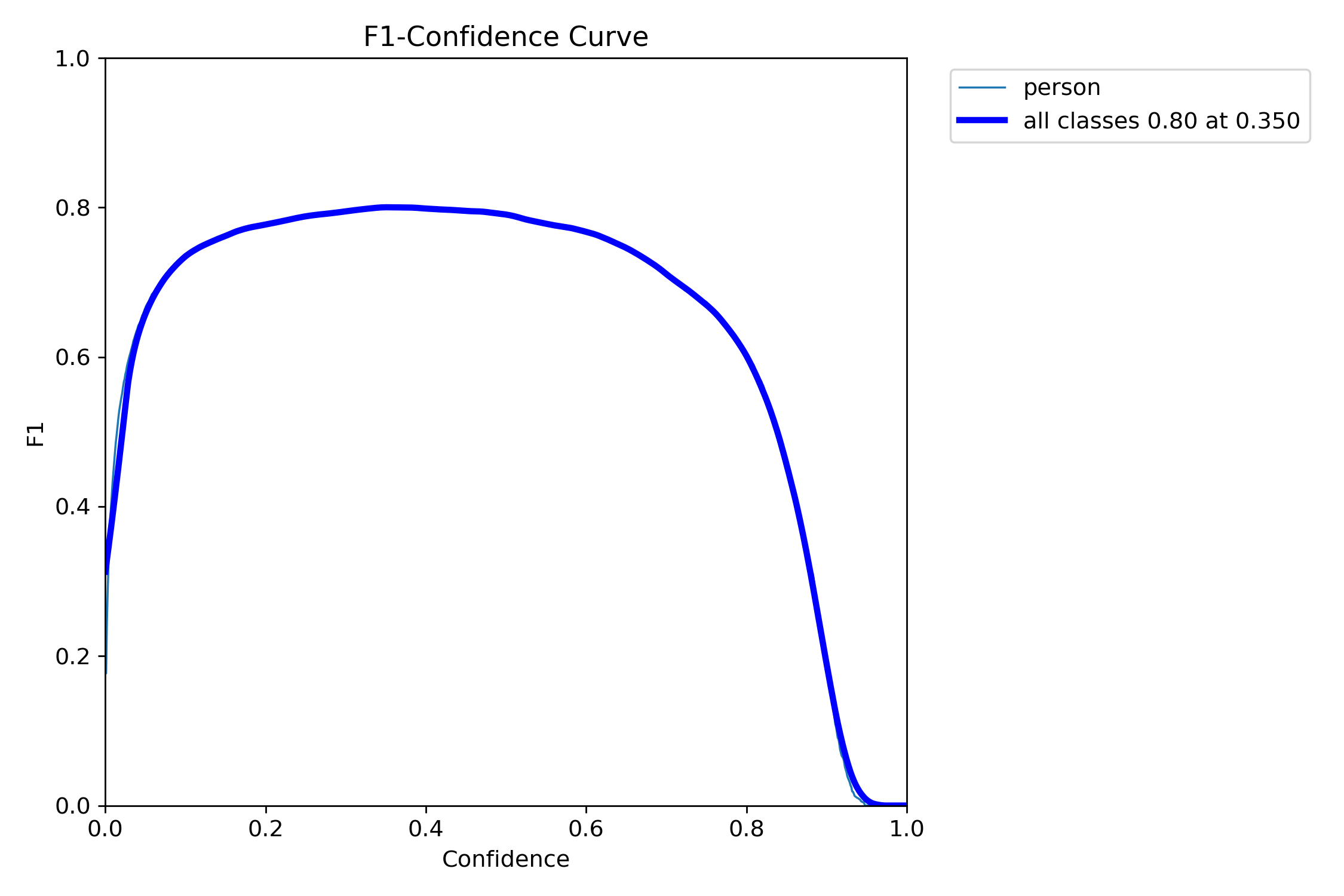

Supplement: S1 File — (ZIP) [file pone.0318578.s002.zip › suooprt information/pose/train34/BoxF1_curve.png]

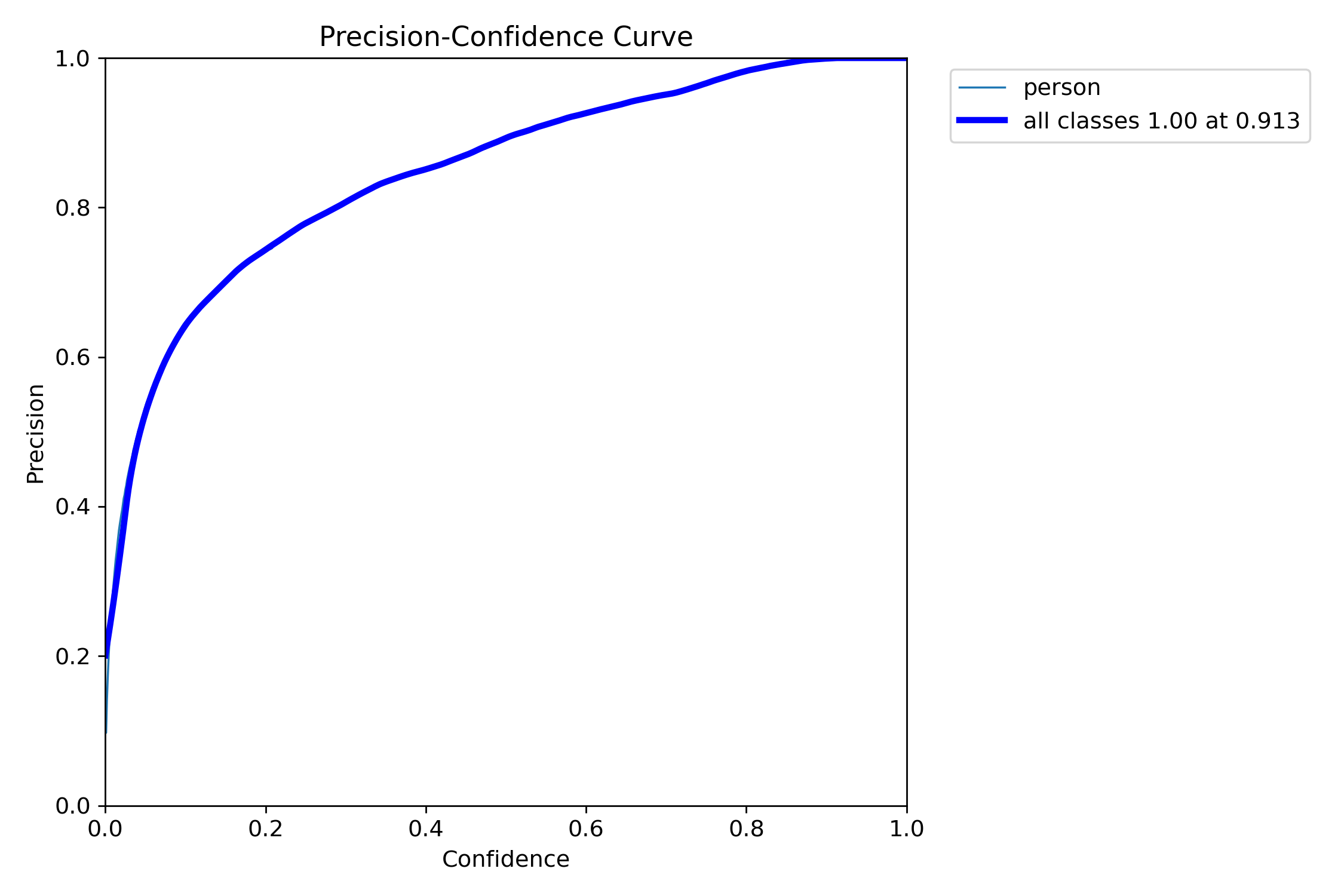

Supplement: S1 File — (ZIP) [file pone.0318578.s002.zip › suooprt information/pose/train34/BoxP_curve.png]
